# Extending universal health coverage to informal workers: A systematic review of health financing schemes in low- and middle-income countries in Southeast Asia

**DOI:** 10.1371/journal.pone.0288269

**Published:** 2023-07-11

**Authors:** Andrea Hannah Kaiser, Niccolò Rotigliano, Steffen Flessa, Björn Ekman, Jesper Sundewall

**Affiliations:** 1 Department of Clinical Sciences Malmö, Division of Social Medicine and Global Health, Lund University, Malmö, Sweden; 2 Deutsche Gesellschaft für Internationale Zusammenarbeit (GIZ) GmbH, Improving Social Protection and Health Project, Phnom Penh, Cambodia; 3 Department of Health Care Management, University of Greifswald, Greifswald, Germany; 4 HEARD, University of KwaZulu-Natal, Durban, South Africa; University of Bologna, ITALY

## Abstract

Achieving universal health coverage (UHC) is a priority of most low- and middle-income countries, reflecting governments’ commitments to improved population health. However, high levels of informal employment in many countries create challenges to progress toward UHC, with governments struggling to extend access and financial protection to informal workers. One region characterized by a high prevalence of informal employment is Southeast Asia. Focusing on this region, we systematically reviewed and synthesized published evidence of health financing schemes implemented to extend UHC to informal workers. Following PRISMA guidelines, we systematically searched for both peer-reviewed articles and reports in the grey literature. We appraised study quality using the Joanna Briggs Institute checklists for systematic reviews. We synthesized extracted data using thematic analysis based on a common conceptual framework for analyzing health financing schemes, and we categorized the effect of these schemes on progress towards UHC along the dimensions of financial protection, population coverage, and service access. Findings suggest that countries have taken a variety of approaches to extend UHC to informal workers and implemented schemes with different revenue raising, pooling, and purchasing provisions. Population coverage rates differed across health financing schemes; those with explicit political commitments toward UHC that adopted universalist approaches reached the highest coverage of informal workers. Results for financial protection indicators were mixed, though indicated overall downward trends in out-of-pocket expenditures, catastrophic health expenditure, and impoverishment. Publications generally reported increased utilization rates through the introduced health financing schemes. Overall, this review supports the existing evidence base that predominant reliance on general revenues with full subsidies for and mandatory coverage of informal workers are promising directions for reform. Importantly, the paper extends existing research by offering countries committed to progressively realizing UHC around the world a relevant updated resource, mapping evidence-informed approaches toward accelerated progress on the UHC goals.

## 1 Introduction

Since the introduction of the 2030 Agenda for Sustainable Development and the sustainable development goals (SDGs) in 2015, the global momentum for universal health coverage (UHC) has continued to grow. This was further solidified by the landmark agreement reached during the High-Level Meeting on UHC at the 74^th^ United Nations General Assembly in 2019 [1]. Governments and policymakers across countries are experimenting with different models for moving toward UHC by utilizing health financing schemes (HF schemes) to protect against financial risk, increase access to quality health services, and improve health outcomes [2]. Health financing is key to attaining the intermediate objectives and goals of UHC, yet many countries encounter difficulties in their progress due to shortcomings in their revenue raising, pooling, and purchasing arrangements [3].

The World Health Organization (WHO) recommends countries adopt compulsory prepayment mechanisms to better distribute the financial risks related to individuals’ varying health expenses across both time and population [4]. Possible prepayment mechanisms include noncontributory financing schemes using general government revenues and contributory options in which individuals pay contributions as a basis for entitlement; both mechanisms offer substantial scope to mobilize resources and allow for broad pooling of prepaid funds and effective cross-subsidization to better spread financial risks associated with the need to use and pay for health services [2, 4].

Irrespective of the choice of prepayment mechanism, many low- and middle-income countries (LMICs) are faced with the challenge of raising revenues in a context with high levels of informal employment. The latter is characterized by labor relations that are mainly based on casual employment, personal and social relations, or kinship rather than on contractual arrangements with formal guarantees: Studies suggest that most workers in informal employment face serious gaps in decent worker protections, including a lack of employment benefits and basic legal and social protection coverage [5]. Additionally, the large heterogeneity in this population with respect to age, gender, poverty, educational attainment, and industry classification, among others, renders it administratively challenging to reach these individuals and to collect public revenue from income-related taxes or regular contributions to health insurance schemes (HISs) [5–7]. One region characterized by a high level of informal employment is Southeast Asia (SEA). A 2018 report by the International Labour Organization estimates that an average of 78% of all men and women work in informal employment, which by definition includes employment in the informal sector and other forms of informal employment in the formal and the household sector [8]. For the sake of simplicity, we will use the term ‘informal worker’ (IW) as an umbrella term for the various forms of informal employment.

In many countries in SEA, achieving UHC is among the top health policy priorities, reflecting governments’ commitments to improved population health through developing and implementing policies that are responsive to individuals’ and communities’ demands and needs for affordable quality health services [9, 10]. Progress has been documented across all Southeast Asian nations, though countries are at different positions on their path to UHC, with several lagging behind. For instance, while Thailand’s and Malaysia’s UHC indices are above 70% and 80%, respectively, Cambodia’s and Myanmar’s indices are below 50% as a consequence of severe restrictions in service coverage [11].

Considerations of equity are further inherent to the pursuit of UHC, aimed at redressing the persistent inequities within and across population groups and geographic areas [12–14]. However, attaining equity is not a natural outcome of the execution of UHC policies; necessary trade-offs on the path to UHC may have adverse consequences for vulnerable groups such as IWs, resulting in a worsening of inequities [12, 15–17]. A common financing and enrollment pattern has emerged across LMICs aiming at expanding population coverage, starting with coverage of civil servants and formal sector employees and with government-subsidized coverage for the poor [6, 10]. This can lead to the creation of coverage gaps for other population groups, most notably near-poor and non-poor IWs and their families, sometimes referred to as the “missing middle” [2, 6, 10, 18]. Where social health protection schemes for IWs exist, these tend to be small and fragmented with poor coordination, leading to challenges in enhancing and sustaining coverage [6, 19, 20]. Considering the precarious situation this presents for IWs in the absence of employment benefits and basic legal and social health protection coverage, covering this gap is key to the pursuance of UHC and the attainment of its goals.

The existing literature contains several reviews on HF schemes aimed at extending UHC to IWs in LMICs, which all have been published before or around the introduction of the 2030 Agenda in 2015. Acharya et al. [20] analyzed the impact of HISs and Vilcu et al. [21] specifically looked at subsidization of HIS via government budget transfers. Bitran [6], Cotlear et al. [18] and Tangcharoensathien et al. [22] comprehensively reviewed the challenges in achieving UHC with a focus on informal employment in SEA and globally. We hypothesize that the recent political will and public support galvanized for advancing UHC in the Southeast Asian region, such as the introduction of a National Health Insurance (NHI) in the Lao People’s Democratic Republic (Lao PDR) in 2016 and the signing of the UHC Act in the Philippines in 2019 [23, 24], has led to the publication of new studies, detailing individual countries’ experiences in extending UHC to IWs and in some cases their families. To incorporate this evidence and extend existing research in this area, the objective of this study is to systematically review and synthesize the published evidence of the extent to which HF schemes aimed at extending UHC to near-poor and non-poor IWs in LMICs in SEA have improved utilization of essential health services and financial protection of IWs and to describe the implementation of the schemes. Findings are synthesized using the conceptual framework for country-level analysis of HF schemes described in McIntyre and Kutzin [25]. We thus aim to offer countries committed to advancing UHC regionally and globally with a relevant updated resource, charting evidence-informed approaches toward the UHC goals in a context of high labor force informality.

## 2 Methods

### 2.1 Protocol and registration

We conducted a systematic review on HF schemes for extending UHC to near-poor and non-poor IWs in LMICs in SEA in line with the Preferred Reporting Items for Systematic Reviews and Meta-Analyses (PRISMA) reporting guidelines (Table 1 in S1 File). The rationale and methods in this study were specified in advance and documented and registered in a protocol in the International Prospective Register of Systematic Reviews (PROSPERO). Registration was obtained on June 11, 2021 (Registration number CRD42021251910).

### 2.2 Information sources

The sources of online data covered electronic databases for articles published in peer-reviewed journals, including Medline, Web of Science, Scopus, JSTOR, Science Direct, and the Cochrane Library. We complemented these searches with additional approaches to identify further relevant publications. We searched for grey literature using the search engines Google and Google Scholar and the websites of relevant institutions (World Bank, International Labour Organization, and WHO), and used citation tracking. Moreover, we screened the reference lists of all included studies and of previous reviews for additional relevant publications (i.e. using the snowball method) [26].

### 2.3 Search strategy, study selection, and eligibility criteria

We used a combination of text words and medical subject headings related to health financing, UHC, and LMICs in SEA for the search strategy which was applied to different databases (see Tables 2–5 in S1 File). For databases that did not offer the possibility of a thesaurus system, we used free text searches with similar search terms. Publications were included in the review if they met all five criteria: (1) were randomized controlled trials (RCTs), non-randomized studies (e.g. quasi-experimental studies, cohort studies, cross-sectional studies, case-control studies), qualitative studies, case studies, policy analysis studies, or reviews, with no study design-related selection criteria applied for grey literature; (2) studied HF schemes for extending UHC to near-poor or non-poor IWs; (3) were carried out in LMICs in SEA (excluding small island states these are Cambodia, Indonesia, Lao PDR, Malaysia, Myanmar, the Philippines, Thailand, and Vietnam); (4) were written in English; and (5) were published in 2010 onwards, coincident with the publication of the World Health Report ‘Health systems financing: the path to universal coverage’ in this year. Given the lack of a clear consensus on a definition for near-poor and non-poor IWs, applied definitions vary at the country level, especially within the scope of UHC reforms [7]. We therefore relied on the definitions used by the authors of the included publications. A caveat to keep in mind in this context is that while certain HF schemes are designed specifically for covering IWs, others extend coverage to the uncovered population more broadly (e.g. the Thai Universal Coverage scheme (UCS)), which may include large numbers of persons who are not in regular work such as children and older people. As far as possible, we extracted and presented information on the implementation of the schemes and their effects for IWs in particular. While the effectiveness of both voluntary health insurance (VHI) and community-based health insurance (CBHI) as national prepayment schemes for UHC have been questioned—especially for coverage of IWs—available evidence demonstrates that both may assist in filling a coverage gap in certain local communities and in providing a smoother political transition [6, 18, 27, 28]; we therefore also considered VHI and CBHI in this systematic review. For the sake of completeness, we included literature on schemes that no longer exist, though we focused on the current schemes in the presentation of the results. For grey literature publications with multiple versions (e.g. health system reviews and/or national health policy documents), we selected the most recent version. For studies published in both peer-reviewed journals and as grey literature, we included the former. Exclusion criteria are listed in Table 6 in S1 File.

We imported the records identified through the searches into the review software Rayyan for screening and reviewing. Two independent reviewers (AHK and NR) screened all titles and abstracts of the records identified initially for potential eligibility, applying the selection criteria. The full texts were retrieved for all papers included in the first screening stage and reviewed by at least one independent reviewer. One of the reviewers (AHK) then applied the eligibility criteria to the full texts of potentially eligible papers, documenting reasons for exclusion; a second reviewer (NR) independently checked these judgements. Any disagreements between the main reviewers were resolved with the use of a third reviewer.

For ease of understanding of the review results, Table 1 illustrates general characteristics of the included Southeast Asian nations. Countries differ in the composition of their health financing system, though they share the common characteristic of a high level of informal employment (excepting Malaysia).

**Table 1:** Overview of select characteristics of the focus countries of the systematic review.

Country	Income status [29]	Population (million) [30]	Informal employment (% of total) [8]	Current health expenditure (% of GDP) [31]	Domestic government health expenditure (% of total) [31]	External health expenditure (% of total) [31]
**Cambodia**	LMIC	15.84	89.8%	6.03%	21.27%	20.54%
**Indonesia**	LMIC	272.25	80.2%	2.87%	49.33%	0.38%
**Lao PDR**	LMIC	7.37	78.5%	2.25%	38.70%	12.45%
**Malaysia**	UMIC	33.36	8.3% [32]	3.76%	51.18%	0.01%
**Myanmar**	LMIC	53.55	82.3%	4.79%	14.83%	8.72%
**Philippines**	LMIC	110.43	62.8% [33]	4.40%	32.65%	0.76%
**Thailand**	UMIC	69.95	54% [34]	3.79%	76.27%	0.33%
**Vietnam**	LMIC	98.32	57.9%	5.92%	45.56%	1.85%

Abbreviations: GDP = gross domestic product; LMIC = lower middle-income country; UMIC = upper middle-income country.

### 2.4 Data extraction and items

We followed the guidance of the Cochrane Collaboration Qualitative Methods Group and developed a standardized data extraction matrix in Microsoft Excel. Details were captured on the following variables: citation information, geographical origin and setting, study aim(s) and objective(s), study design and methods, study population(s) and target group(s), setting, description of HF schemes, policy scale, starting year and timeframe, main results, conclusions, and recommendations. One reviewer (AHK) independently extracted all data, which was cross-checked by a second reviewer (NR).

### 2.5 Quality appraisal

We used the Joanna Briggs Institute critical appraisal tools for the quality appraisal and risk of bias assessment of all included peer-reviewed articles. The tools provided a coherent set of checklists to appraise key methodological aspects of studies with various designs and to assess the trustworthiness, relevance, and results [35]. Two reviewers (AHK and NR) independently carried out the quality appraisal, with disagreements or discrepancies being resolved through consensus. To facilitate comparison across studies, we grouped quality scores in three categories: low (<60%), medium (≥60% and <80%), and high (≥80%). We intended to provide a comprehensive overview of the available evidence and its quality, and hypothesized that every article may provide valuable findings, experiences, and recommendations on the HF schemes implemented to extend UHC for IWs. Thus, notwithstanding low-scoring articles, we included all studies in data analysis and synthesis. No quality appraisal was carried out for grey literature publications.

### 2.6 Data analysis and synthesis

The heterogeneity in the countries, settings, study designs, and outcome measures, among other comparisons reported on in the included articles precluded a formal a meta-analysis. Instead, we used thematic analysis, also known as directed qualitative content analysis, to organize and synthesize the extracted data [36]. We used the conceptual framework for country-level analysis of HF schemes described in McIntyre and Kutzin (2016) to derive initial coding categories [25]. The framework illustrates the UHC goals and intermediate objectives that a country’s HF scheme(s) can influence to make progress toward UHC. The application of this framework has been shown to be useful in the analysis of health insurance and other HF schemes in preceding reviews [21, 37–39]. For our data analysis and synthesis, we focused on the three health financing functions: revenue raising, pooling of funds, and purchasing of services, including health benefit package (HBP) design as an element of purchasing. Table 2 illustrates the categories used in the thematic analysis.

**Table 2:** Conceptual framework for country-level analysis of HF schemes. Adapted from [2, 25, 40, 41].

**Revenue raising arrangements for UHC—Sources of funds, contribution methods, and mechanisms for their collection**	
Source of revenue	Direct tax revenue (e.g. income tax, payroll tax, corporate income / profit taxes, mandatory social health insurance contributions)
	Indirect tax revenues (e.g. VAT, sales taxes, excise taxes, import duties)
	Other government revenue (e.g. natural resource revenues)
	Financing from external sources
	Other compulsory contributions
	Voluntary prepaid contributions
**Pooling arrangements for UHC—Accumulation of prepaid funds on behalf of a population**	
Type of pool	Degree of fragmentation (e.g. single, multiple), size and composition of pools (e.g. population groups covered), level of cross-subsidization
**Purchasing arrangements for UHC—Transfer of funds to the provider on behalf of a population**	
Provider payment method	Allocation of resources to providers (e.g. using capitation, fee-for-service, DRG, salary, global budget) and degree of strategic purchasing
Health benefit package design and rationing	Health service entitlements and obligations of the population as well as differences across pools
	Degree of cost-sharing and mechanisms (e.g. user fees, copayment and deductible rates, referral requirements)

Abbreviations: DRG = diagnosis-related group; HF scheme = health financing scheme; UHC = universal health coverage.

To categorize the effect of the included HF schemes on progress toward UHC, we considered the three dimensions of (1) financial protection, (2) population coverage, and (3) access to health services, outlined by WHO as indicators for measuring country-level progress toward UHC [11, 42]. For the sake of consistency in the methodology of calculating financial protection indicators, we took data on out-of-pocket expenditure (OOPE), catastrophic health expenditure, and health-related impoverishment from the 2019 Global Monitoring Report on Financial Protection in Health [43]. Further, we followed the approach suggested by Shengelia et al. to use utilization rates as a proxy measure for access to health services in the absence of data disaggregated by utilization with and without true need [44]. Insofar as available, data on the effects of the analyzed HF schemes were taken from peer-reviewed, published articles that were quality-appraised; we marked data taken from grey literature with an asterisk (*). Information related to the categories and UHC indicators was synthesized and presented using text, tables, and graphs.

## 3 Results

**Fig 1:**
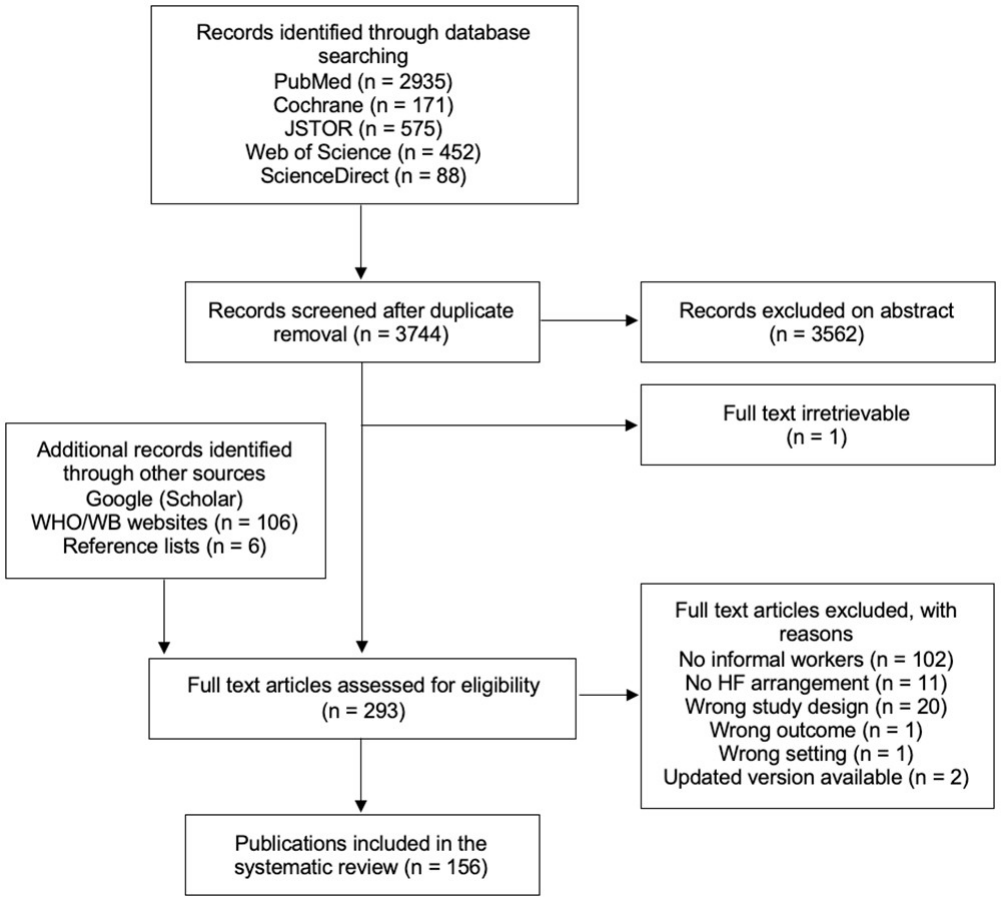
Flow diagram for selection of publications. Adapted from Tricco et al. [45]. Abbreviations: HF = health financing; WB = World Bank; WHO = World Health Organization.

The electronic database searches generated 4221 studies and we located a further 112 papers through the other search forms described earlier. Following deduplication, we excluded 3534 records as not relevant in the title- and abstract-screening stage. We then assessed the full texts of 321 publications for eligibility and found 156 to meet the eligibility criteria for this systematic review. Most publications were excluded because they did not provide information specific to IWs. The flow of selection is summarized in Fig 1.

List 1 in S1 File provides the full reference list of the 156 included publications, and List 2 in S1 File shows publications excluded at the full-text stage. Sixty-seven are peer-reviewed articles, of which the majority (n = 43) employed quantitative research methods (e.g. cross-sectional surveys or impact evaluations). An additional 14 articles utilized qualitative methods (e.g. qualitative interviews and focus group discussions), and ten reported on mixed methods studies. Table 7 in S1 File summarizes methodological details and key findings extracted from the included peer-reviewed articles. The remaining 89 publications comprise of grey literature such as health system and/or health financing reviews, national policy and strategy documents, policy analyses, reviews, and discussion/working papers. Most publications (n = 107) were published in or after 2015, suggesting that research on extending UHC to IWs has increased over time as a result of key political UHC milestones.

**Fig 2:**
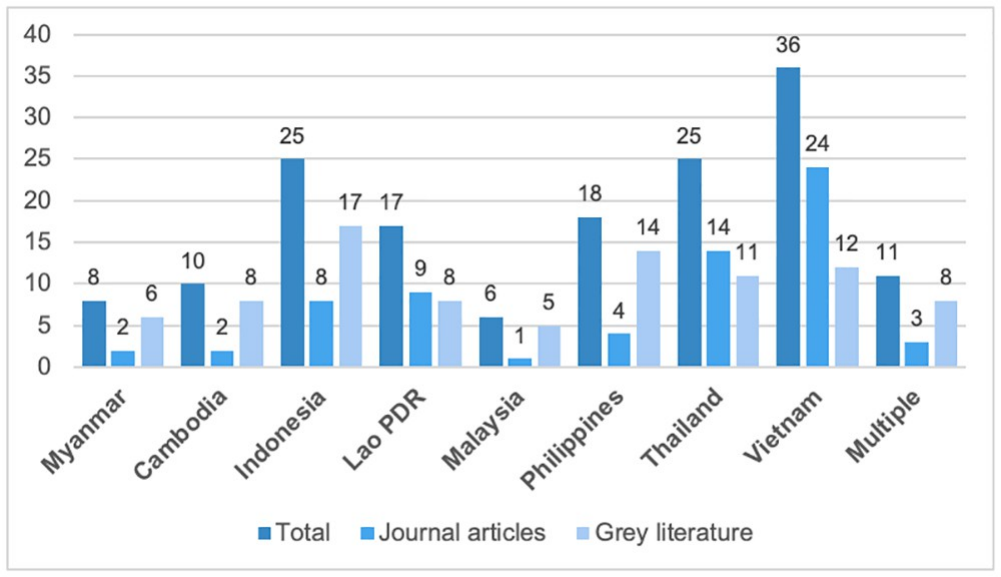
Number of included publications per country.

The literature searches yielded multiple publications for each country, enabling data triangulation of both the design of the HF schemes and changes in the three UHC dimensions. However, the number of publications indicated marked variation between countries, reaching from six in Malaysia to 36 in Vietnam (Fig 2). One explanation for the low number of relevant publications for Malaysia may be the higher formalization of its economy with a correspondingly low share of informal employment (8.3% in 2019) [32] (Table 1). Eleven publications reported on multiple countries in the Southeast Asian region.

### 3.1 Summary of the quality appraisal

Overall study quality of the peer-reviewed articles was medium. Applying the set of Joanna Briggs Institute checklists, the results indicated that a majority obtained a medium (n = 27) or high (n = 26) quality score, while the remaining 14 articles fell into the low-quality category (<60%). Only two studies were RCTs to identify causal mechanisms and both obtained a high quality rating [46, 47]. Many quantitative studies failed to address areas of potential bias, most notably potential confounding factors and strategies to deal with these within the study design or data analysis. The breakdown of articles and their quality scores are shown in Table 7 in S1 File.

### 3.2 Overview of HF schemes

#### 3.2.1 Mode of participation, entitled groups, and basis for entitlement

For the eight focus countries of the systematic review, we included eight HF schemes that are currently in place with the aim of extending UHC to near-poor and non-poor IWs. Table 3 gives an overview of these HF schemes. From a policy perspective, the main distinguishing criteria to categorize HF schemes are the mode of participation and the basis for entitlement. The mode of participation refers to the relation between the HF scheme and eligible individuals. It can be categorized as (1) compulsory/mandatory, in which population coverage is either automatic and universal (e.g. National Health Service (NHS)) or acquired against contribution payments (e.g. social health insurance (SHI)), or (2) voluntary, where coverage is at the discretion of individuals (e.g. VHI) [48]. The mode of participation affects the size and the composition of the risk pool (e.g. demographic, socioeconomic, health risks) [21, 25]. Lao PDR, Indonesia, the Philippines, Thailand, and Vietnam operate single-payer NHI or SHI schemes established by national legislation and/or enshrined in the national constitution. All five schemes offer universal eligibility for the entire IW population and their dependents [24, 49–54]. The current health financing setups in Lao PDR, Indonesia, the Philippines, and Vietnam are the result of major health financing reforms between 2014 and 2019 with strong commitments to providing UHC to the entire population and are still partially in the process of being implemented. Malaysia’s health system operates according to an NHS model, targeting the entire population inclusive of IWs and their dependents [24]. Notably, membership in these six HF schemes is mandatory by law [24, 49–52, 55–57]. However, despite that participation is *de jure* mandatory in Indonesia’s Jaminan Kesehatan Nasional (JKN) and Vietnam’s SHI, both schemes are facing challenges with a lack of enforceability since no penalties for non-participation are in place, creating *de facto* voluntary schemes [49, 56, 57].

**Table 3:** Overview of HF schemes for extending UHC to IWs in LMICs in SEA.

Country	HF scheme	Entitled IW groups	Mode of participation
**Cambodia**	HEF extension (Regulation 404), 2018	Near-poor and non-poor IWs classified as casual, seasonal, or part-time [58]. Dependents are not covered [59].	Voluntary [58].
**Indonesia**	NHI (Jaminan Kesehatan Nasional), 2014	JKN targets the entire near-poor and non-poor IW population, including dependents [49].	Mandatory [49].
**Lao PDR**	NHI, 2016	NHI targets all IWs with dependents through merger and consolidation of pre-existing schemes in 2019 (HEF, CBHI, Free MNCH services) [50, 51].	Mandatory with default enrolment of all Laotians [50, 51].
**Malaysia**	NHS, 1957^a^	NHS targets the entire population, including IWs of all income levels [52, 53].	Mandatory. No opt-out for non-users [52].
**Myanmar**	SSS, 1956 (new Social Security Law in 2012)	De jure coverage of IWs, excluding dependents [60].	Voluntary [60].
**Philippines**	NHIP, 1995 (UHC Act in 2019)	NHIP targets all IWs defined as self-earning, market vendors, street hawkers, market vendors, construction and migrant workers, pedicab/tricycle drivers, and those in home-based services; qualified dependents as declared by the principal member are included [24].	Mandatory. Automatic and default eligibility of all Filipinos under the UHC Act [24].
**Thailand**	UCS, 2002	All IWs at all income levels and their dependents are universally eligible for coverage under the UCS [54].	Mandatory. Granted through right of citizenship [55].
**Vietnam**	SHI, 1993 (law amended in 2014)	All IWs with dependents are universally eligible under the SHI [56, 57].	Mandatory [56, 57].

Abbreviations: HEF = Health Equity Fund; HF = health financing; IW = informal worker; JKN = Jaminan Kesehatan Nasional; LMICs = low- and middle-income countries; MNCH = Maternal, Neonatal, and Child Health; NHI = National health Insurance; NHIP = National Health Insurance Program; NHS = National Health Service; PLHIV = people living with HIV; SHI = Social Health Insurance; SEA = Southeast Asia; SSS = Social Security Scheme; UCS = Universal Coverage Scheme; UHC = universal health coverage.

^**a**^ The development of Malaysia’s health system has begun before independence; the year of independence was therefore assumed as inception year.

In Cambodia, the government introduced extensions of the existing Health Equity Funds (HEFs) for the poor in 2018 to provide coverage for a number of clearly defined IW groups (excluding dependents). Participation in this scheme is voluntary as per the legal provisions [58, 59]. Myanmar offers *de jure* voluntary enrolment for IWs in the Social Security Scheme (SSS). In practice, however, there are presently no mechanisms in place for IWs to register with SSS [60] and thus no HF scheme effectively covering IWs in Myanmar [60, 61]. Therefore, the narrative synthesis will mainly focus on the remaining HF schemes, though information on SSS is included in the tabular result illustration.

The basis for entitlement relates to the basic conditions for access to health services under HF schemes. This can be either contributory and require a payment of contribution on behalf of or by the individual to be covered (e.g. SHI) or noncontributory, where coverage is not linked to contribution payments but defined for citizens/residents or specified groups within the country [48]. Indonesia’s JKN, the Philippines’ National Health Insurance Program (NHIP), and Vietnam’s SHI are contributory, requiring IWs to prepay contributions as a prerequisite for gaining access to health services at facilities covered under the schemes [24, 60, 62–68]. Cambodia’s HEF extension, Lao PDR’s NHI, Malaysia’s NHS, and the Thai UCS are noncontributory systems, relying predominantly on tax-based financing [52, 54, 58, 69–72]. More information on financing sources is provided in a subsequent section on revenue raising.

#### 3.2.2 Enrolment procedures and governance structures

The enrolment process in the eight HF schemes can be broadly divided into two categories: (1) Active, where the IWs themselves are required to seek enrolment with the relevant authority, and (2) Automatic, where beneficiaries are automatically enrolled by responsible authorities once eligibility is confirmed, with little or no involvement in the process. In four of the eight included HF schemes (Cambodia’s HEF Extension, Indonesia’s NHI, and Vietnam’s SHI), beneficiaries must actively seek enrolment/registration, generally by visiting the competent authority [49, 56, 58, 62, 73, 74]. Enrolment in the Filipino NHIP and Thailand’s UCS is automatic, though registration with PhilHealth and the Thai National Health Security Office is still required [66, 75–77]. Only Lao PDR offers fully automatic enrolment in its NHI [78]. No formal enrolment is required in Malaysia’s NHS [52, 72]. (Quasi-) government organizations are responsible for beneficiary identification and enrolment/registration in as well as management and administration of all analyzed schemes, with Table 4 illustrating additional details.

**Table 4:** Enrolment process and organization(s) responsible for enrolment and management.

Country, HF scheme	Organization(s) responsible for identification and enrolment	Enrolment process	Organization(s) responsible for management and administration
**Cambodia** HEF extension	NSSF [58].	Active. IWs need to physically visit one of the NSSF offices in Phnom Penh or in the provinces to seek registration with the scheme [58].	Governance authority lies with the various HFSCs established at provincial and district levels; the HFSC in Phnom Penh is the last reference point for issues that cannot be solved at lower levels [79]. MOH holds the executive authority over the HEF. Internally authority is shared between the Department of Planning and Health Information (benefit package and provider payment) and Department of Budget and Finance [79]. The PCA (semi-autonomous under MOH) is responsible for verification and certification of claims submitted by providers [58, 79].
**Indonesia** JKN	BPJS-K [49].	Active. IWs need to seek enrolment with BPJS-K, either in-person in an office, or through a mobile device app [49, 73].	BPJS-K at the national level. At the local level, local district governments have decision-making and implementation autonomy in health service delivery [49].
**Lao PDR** NHI	NHIB under MOH [23].	Automatic. IWs are eligible when they use health services based on the rules of the NHI with no formal enrolment required [78].	Central, Provincial, Vientiane Capital, and District and Municipality NHIB. Integration of the NSSF and transfer of health schemes covering formal sector employees to NHIB is in progress [23, 51].
**Malaysia** NHS	Not applicable. All citizens are eligible for free or subsidized services at public facilities [52, 72].	No formal enrolment required. All Malaysians are entitled to access health services at public health facilities upon proof of identity and citizenship using the national identity card [52, 72].	National-level policymaking, regulation, and planning are centralized in MOH, the primary provider of public health services. Amid de-concentration to the subnational level, health departments and district offices manage district-level public health, collective health services, and critical service delivery units [71].
**Myanmar** SSS	SSB under Ministry of Labor, Employment and Social Security [80].	Not available. There are currently no mechanisms in place for IWs to register themselves with SSB [60].	SSB under Ministry of Labor, Employment and Social Security administers the health insurance function of the SSS, including benefit definition and contributions, and manages healthcare facilities accessible for registered beneficiaries [80].
**Philippines** NHIP	PhilHealth Corporation and DSWD [24, 66, 75, 76].	All Filipinos are automatically covered under the NHIP, though still need to actively seek registration with PhilHealth [66, 75, 76].	PhilHealth Corporation is mandated to implement the NHI and acts as the principal government agency that purchase health services on behalf of its members [24, 76].
**Thailand** UCS	NHSO under NHSB and SOCB [55].	Thai citizens not covered by the civil servant or the SHI schemes are automatically enrolled under the UCS, though registration is required [77].	NHSO under NHSB and SOCB. Key features of NHSO administration are design and management of the benefit package and provider payment methods, budgeting, billing, clinical auditing, quality assurance, and customer right protection [55].
**Vietnam** SHI	VSS under the Ministry of Health and Finance [56, 74].	Active. Enrollees seek enrolment in SHI with the VSS [56].	SHI policymaking resides with MOH. VSS is responsible for all necessary tasks for organizing the implementation of SHI policies, including inter alia contribution collection, fund usage and management, claims management, and provider contracting [56, 74].

Abbreviations: BPJS-K = Badan Penyelenggara Jaminan Sosial Kesehatan; CSMBS = Civil Servant Medical Benefit Scheme; DSWD = Department of Social Welfare and Development; HEF = Health Equity Fund; HFSC = Health Financing Steering Committee; JKN = Jaminan Kesehatan Nasional; MOH = Ministry of Health; NGO = non-governmental organization; NHI = National Health Insurance; NHIB = National Health Insurance Board; NHIP = National Health Insurance Program; NHS = National Health Service; NHSB = National Health Security Board; NHSO = National Health Security Office; NSSF = National Social Security Fund; PCA = Payment Certification Agency; SHI = Social Health Insurance; SSB = Social Security Board; SOCB = Standard and Quality Control Board; SSS = Social Security Scheme, UCS = Universal Coverage Scheme; VSS = Vietnam Social Security.

### 3.3 Financing arrangements, including revenue raising and level of subsidization

In addition to beneficiaries’ contributions, revenue sources for the analyzed HF schemes include mainly general government tax revenues. These comprise of direct (e.g. personal income and company taxes) and indirect taxes (e.g. sales, import/export, and excise duty taxation) (Table 4). Notably, the Philippines’ supplemental funding to NHIP includes mainly sin tax revenues, an excise tax on tobacco, alcohol, and sugar sweetened beverages [81]. OOPE paid by patients constitute another major source of revenues in all HF schemes, apart from the Thai UCS.

Given the (on average) low financial capacity of IWs, other crucial aspects are the level of subsidization and any remaining co-payment requirements (see also section on HBP design). In three out of eight schemes, IWs of all income levels receive full subsidies from public revenues (Cambodia’s HEF Extension, Lao PDR, and Thailand), with no registration fees or co-contributions [54, 58, 59, 69]. In contrast, in Myanmar, IWs are required to pay full insurance premiums [62, 75, 80]. Malaysia’s NHS health system is a special case in that it provides large government subsidies benefitting the entire population [52, 53]. The remaining three HF schemes in Indonesia, the Philippines, and Vietnam offer full or partial subsidies to near-poor IWs, while non-poor IWs are required to pay full contributions [56, 63–65, 82, 83]. The financing sources for subsidies are mainly from general, central government revenues. The Philippines also deploy part of their sin tax revenues to subsidize coverage of near-poor IWs, with rapid increases in coverage in its first four years of implementation [81, 83]. See Table 5 for additional details.

**Table 5:** Revenue raising method(s), level of subsidization for IWs, and pooling arrangements.

Country, HF scheme	Revenue raising method(s) for HF scheme and basis for benefit entitlement for IWs	Level of subsidization for IWs	Pooling arrangements
**Cambodia** HEF extension	Noncontributory social health protection scheme financed through the national budget of the Royal Government of Cambodia. The budget is separated from the HEF budget for service provision to the poor [58].	Full public subsidy for eligible IW groups [58, 59].	Multiple pools. Pools are currently separate with no cross-subsidization between population groups (formal sector, IWs, the poor, etc.) across the different HF schemes implemented in Cambodia [59].
**Indonesia** JKN	Contributory system combined with government subsidies for the poor and near-poor. Depending on their choice of inpatient ward class and payment plan (hospital beds and facilities), non-poor IWs pay nominal contributions of between Rp42,000-Rp150,000 per month; contributions for civil servants and formal workers are based on payroll tax at 5%. Also included are government tax contributions (income and tobacco tax), district-level payments and grants from development agencies [63–65].	Full subsidies paid by government revenues are in place for near-poor IWs. Non-poor IWs are required to pay regular contributions [63–65].	Single trust fund (Dana Amanat) comprised of contributions of the entire population, including subsidies. BPJS-K serves as fund manager and pools contributions received by the different types of members, including public subsidies for the poor and near-poor [84].
**Lao PDR** NHI	Predominantly tax-based financing (i.e. noncontributory for IWs) combined with remuneration- based contributions from workers in formal employment. Includes funds from bi- and multilateral aid through the government [69, 70, 87].	Large government subsidies transferred to NHI fund on behalf of IWs [69].	Single pool under NHI through merger of formal-employment sector schemes (civil servants and private sector employees previously covered by NSSF) [51].
**Malaysia** NHS	Predominantly tax-financed system through mainly un-earmarked general direct (personal income, company, and petroleum income tax) and indirect taxation (e.g. sales tax, import/export and excise duties) and non-tax revenues collected by the federal government [52, 71, 72].	Public health system heavily subsidized by government, benefitting all population groups [52, 53].	Public funds are pooled in that MOH handles the largest share of public sector health funds and pools resources across the federation before distribution to state providers, enabling geographic pooling with cross-subsidies between regions. In principle, this system also cross-subsidizes across individuals in varying age groups and of different health/income status. De facto pooling is limited. The broader pooling within the public sector contrasts the situation in the private sector (38% of THE not pooled due to the high level of OOPE) [71, 72, 86].
**Myanmar** SSS	Contributions levied of employee salaries and matched at same rate by employers (2% for workers <60 and 2.5% for workers aged 60 or older). Self-employed persons pay 4% (5% if aged 60 or older). The monthly legal minimum wage is used to calculate contributions for minimum monthly earnings; maximum monthly earnings at threshold of 300,000 kyats ($182) [80].	No subsidies are available for IWs [80].	No formal pooling arrangement [88].
**Philippines** NHIP	Contributions are paid by direct contributors (employees, self-employed, informal, and migrant workers) at currently 3.5%; the UHC law schedules a gradual increase in contribution rates up to 5% in 2025. Monthly payment for indirect contributors (indigents and special sponsored groups) are paid by national government funds; the latter includes mainly ‘sin tax revenues’ (excise tax on tobacco, alcohol and sugar sweetened beverages of which 85% are channeled to health), funds from the Philippine Charity Sweepstakes Office and the Philippine Amusement and Gaming Corporation [24, 66, 67].	Subsidies are available for indigents, sponsored members (mainly lower income segment IWs), and other special groups (means test rule). IWs above the poverty line pay contributions based on their capacity to pay within the minimum and maximum contribution thresholds [75, 83].	Resources for health are channeled into multiple pools. At the national level, PhilHealth and several national government departments and agencies hold big pools. Premium collections and infusions are pooled into one pool for PhilHealth as a source for all benefits payments. Local government units hold their own fund pools from locally generated revenues and fiscal transfers from the national level; pools are either for specific groups or for everyone [67]. To improve coordination, funds will be pooled at provincial level under the UHC Act through establishing a ring-fenced Special Health Fund [24].
**Thailand** UCS	Tax-based, noncontributory system for poor, borderline poor, and non-poor IWs. General tax revenue from direct and indirect sources is the dominant financing source, mainly based on personal income taxes with progressive tax rates with respect to population incomes [54].	IWs are fully subsidized, while members of the civil servant and the SHI scheme are required to pay contributions [54].	Segmented health insurance schemes with separate pools for UCS, SHI, and CSMBS with explicit coverage for all. There are no cross-subsidies between different pools and across populations [54].
**Vietnam** SHI	Contributory system for many different population groups with contributions based on individuals’ remuneration at 4.5%-6%. For IWs, the insurance premium of 4.5% is applied to the minimum wage, generally translating into low contribution rates. The family-based contribution for family health insurance is at 4.5% of the national minimum wage for the first member (max. VND 45,450), followed by discounted descending rates from 40% to 70% for the 2^nd^ to 5^th^ member. Contributions are supplemented by government budgetary transfers [56, 68, 82].	Contributions are partially subsidized through public transfers for family-based insurance policyholders (30–70% subsidy) and near-poor IWs (70% subsidy) with an income less than 30% above that of poor people [56, 82].	Merged into a single national fund in principle. In practice there are multiple fragmented pools (61 provincial funds retaining 90% of the funds) with limited risk-sharing and reverse cross-subsidization. Equalization of funds through central reserves is minimal and redistribution effects therefore in practice often regressive (poorer to richer regions or groups). The fragmentation is worsened by the large number of membership categories with differential contributions [56, 85].

Abbreviations: BPJS-K = Badan Penyelenggara Jaminan Sosial Kesehatan; HEF = Health Equity Fund; HF = health financing; IW = informal worker; JKN = Jaminan Kesehatan Nasional; MNCH = Maternal, Neonatal, and Child Health; NHI = National health Insurance; NHIP = National Health Insurance Program; NHS = National Health Service; NSSF = National Social Security Fund; OOPE = out-of-pocket expenditure; PLHIV = people living with HIV; Rp = Indonesian rupiah; SHI = Social Health Insurance; SSS = Social Security Scheme; THE = total health expenditure; UCS = Universal Coverage Scheme; UHC = universal health coverage, VND = Vietnamese dong.

Considering revenue raising methods and subsidies together, HF schemes can be categorized into three broad, practical approaches to extending UHC discussed in relevant literature: (1) extending coverage down from civil servants and the formal sector; (2) using a combination of prepayment and tax-based subsidies; and (3) extending coverage upward from schemes subsidizing the poor [7, 27]. Four of the schemes fall into the second category, using a combination of prepaid insurance premiums and full (Lao PDR) or partial tax-based subsidies for IWs (Indonesia, Vietnam, and the Philippines) [24, 56, 63–70, 82]. Thailand’s UCS and Cambodia’s HEF extension employ a bottom-up approach, extending full government subsidies from the poor to IWs above the poverty line [54, 58, 59]. Only Myanmar is aiming to extend coverage down from civil servants and the formal sector once enrolment will be opened to IWs [60].

### 3.4 Pooling arrangements

A variety of pooling arrangements exist in the analyzed HF schemes, which can be broadly classified into single- and multiple-pool arrangements. Only in Indonesia, IWs are included into a single national pool with contributors and/or beneficiaries of other population groups (e.g. individuals in formal employment) [84]. In the Philippines, resources are currently channeled into multiple fragmented pools at both national- and local government-level, but will be jointly pooled at provincial level through establishing a ring-fenced Special Health Fund once the 2019 UHC Act is fully implemented [24, 67]. Lao PDR will similarly move toward a single pool arrangement upon complete implementation of the NHI [51]. Vietnam has in theory a single national fund, though in practice faces challenges with a large number of pools fragmented along subnational lines [56, 85]. A similar situation has arisen in Malaysia with *de facto* limited pooling and effective cross-subsidization [71, 72, 86]. The Cambodian and Thai HF schemes operate segmented schemes for different population groups with separate pools and similarly no cross-subsidization between schemes and hence across populations [54, 59, 62]. Refer to Table 5 for country details.

### 3.5 Health benefit package design including health service coverage and co-payment requirements

Table 8 in S1 File provides an overview of the scope of the HBPs for all HF schemes covered in this review. In Malaysia’s NHS system, there is no clear-cut, defined HBP and patients have no legally enforceable right to access specific services [52, 71]. All other countries have an explicitly defined HBP within the scope of the analyzed schemes. HBPs are generally broad and comprehensive, including preventive care, curative outpatient and inpatient services at various care levels, and drugs contained in countries’ essential medicines lists. Several HBPs also comprise of promotive, rehabilitative, and palliative services. However, evidence for several schemes demonstrates a discontinuity between the defined HBP promised in theory and available resources. Moreover, five of the eight schemes further defined negative lists to specify the services not covered.

HBPs are defined by the government in all HF schemes, though based on differing national processes. Notably, Thailand’s UCS is currently the only HF scheme reviewed in this study that bases HBP decision-making on transparent procedures and systematic assessment methods for inclusion or exclusion of services and/or for price negotiations of products (e.g. economic evaluation tools in health technology assessments (HTAs)) [54, 55]. The introduction of new technologies offered in Malaysia’s NHS, as well as interventions included in the Z-Benefits package and pharmaceuticals within the Filipino NHIP are also informed by processes resembling HTA [24, 52, 66, 71, 76].

Several HF schemes have co-payment requirements (user charges) in place, requiring service users to pay nominal fees or a certain percentage of their health service expenses (Table 8 in S1 File). Such requirements exist in Indonesia, Lao PDR, Malaysia, and Vietnam, with markedly differing rates and regulations across countries; requirements generally also apply to subsidized members. The Vietnamese SHI distinguishes between different income groups, with reduced fees for near-poor IWs [56]. Moreover, while a no-balance billing policy is in place in the Filipino NHIP, facilities can stipulate their own service costs with NHIP members required to pay the balance between total cost and NHIP benefits; this leaves room for co-payments to persist, shifting the financial risk onto patients [76].

### 3.6 Purchasing arrangements

Purchasing arrangements, including the degree of strategic purchasing and the choice of provider payment mechanisms, are unique in design in the HF schemes, with country details summarized in Table 8 in S1 File. Purchasing arrangements nevertheless share several characteristics: (1) primary healthcare (PHC) is reimbursed through country-specific capitation arrangements, either with and without risk-adjustment mechanisms or performance-based elements, in five of the eight HF schemes (Indonesia, Lao PDR, Philippines, Thailand, and Vietnam) and (2) secondary care is compensated through case-based payments (diagnosis-related groups) under global budgets in the same schemes, except for Vietnam’s SHI [50, 55, 65, 66, 89–93]. In contrast, PHC in Cambodia’s HEF extension is reimbursed on the basis of fixed case-based payments and fee-for-service [59]. In Vietnam, reimbursements for curative secondary care are largely paid on a fee-for-service basis [82, 94]. Financing of Malaysia’s NHS is largely built upon global line-item-based budgets on a historical spending basis [52, 71, 72].

In terms of service delivery, the Cambodian scheme, Lao PDR, Malaysia, and Vietnam generally rely on service provision by public providers only [50, 59, 71, 82], while Indonesia, the Philippines, and Thailand rely on a mixed system with both public and private providers (Table 8 in S1 File) [76, 89, 95]. Overall, the strategic purchasing function has remained largely passive in all analyzed schemes apart from Thailand’s UCS.

### 3.7 Progress on UHC indicators

The following sections explore progress toward UHC realized under the eight HF schemes, categorized by the three indicators: population coverage, financial protection, and access to health services (utilization). For many HF schemes, the available evidence is sparse, and the account of progress made gives a broad indication of trends that should be interpreted with caution. Data on changes in CHE and impoverishment for IWs since the introduction of the HF schemes is not available for all countries. Importantly, for those countries in which civil servants and formal sector employees are covered under separate designated HF schemes (e.g. Thailand, Lao PDR), the sections on financial protection and utilization present data differentiated along income quintiles (primarily for the 2^nd^ and 3^rd^ quintiles), assuming that these largely comprise of IWs and their dependents.

#### 3.7.1 Population coverage

The share of the total population registered under the analyzed HF schemes comprises of both formal and informal workers covered as of the total population in a country, with rates differing considerably between countries and HF schemes (Table 6). Malaysia (100%, 2021) has the highest coverage rate, covering the entire population [52]. Lao PDR (94%, 2018) and Vietnam (90.9%, 2020) also have high rates at above 90% [69, 70, 96], followed by the Philippines at 85% and Thailand at 76% [97]. Cambodia and Myanmar currently have the lowest population coverage under the included HF schemes at 0.26% (HEF extension, 2020) and 5% (SSS, 2017), respectively [60, 98, 99]. In this context it is noteworthy that countries with fragmented SHP landscapes may have additional, separate HF schemes for formal workers and other groups (e.g. the National Social Security Fund in Cambodia or the Social Security Scheme in Thailand) which raise the total population coverage, though these fall outside of the scope of this systematic review.

**Table 6:** Population coverage rates and uninsured population groups under the HF schemes.

Country, HF scheme	Registered IWs as a share of eligible group	Share of total population registered under HF scheme (formal and informal)	Population groups without coverage under HF scheme
**Cambodia** HEF extension	5.12% (2020) [100]	0.26% (43,239) (2020) [100]^a^	HEF extension has so far failed to achieve significant population coverage of the designated groups. Additionally, non-poor self-employed IWs and other IWs working full-time are ineligible under the HEF extension and currently not covered under any other HF scheme.
**Indonesia** JKN	32.3 million (% share not available) (2019) [104]	83.6% (222,500,000) (2019) [69]	After initial fast membership growth of IWs (30.78%/month), the growth has slowed down to 6.55% (2015) and 2.17% (2016); dropout rates for these groups are also high (47% of members discontinued premium payments in October 2018). About 48.1% of IWs were uninsured in 2016 [112] and make up the bulk of the individuals who are currently uncovered under JKN (43.6 million, 2019); the highest number of uninsured people is part of the lower middle-income group [65, 103, 104].
**Lao PDR** NHI	No data available^^b^^	94% (2018) [69, 70]	No data available^#^
**Malaysia** NHS	100% (2021) [52, 71]	100% (2021) [52, 71]	None. All Malaysians are entitled to access public health services for free or at low nominal user fees [52, 71].
**Myanmar** SSS	Negligible [60, 101]	5% (1,089,559) (2017) [60]	No system has been established yet for IWs to register themselves directly to Myanmar’s contributory SSS. Additionally, information on the scheme is not easily available. Only a negligible share of the eligible IWs is aware of the scheme and, though entitled by law, they are thus effectively excluded [60].
**Philippines** NHIP	18,146,517 (2020, % share not available) [124]^^b^^	85% (93,372,092) (2020) [124]	Despite that every Filipino is automatically included under the NHIP since the signing of the UHC Act, about 11 million are currently not registered. The composition of this group is not known, though near-poor and self-employed IWs are expected to make up a large share [66]. Lack of awareness of and inability to navigate the health system to access their entitlements have been reported as main reasons for the coverage gaps [66, 67, 76]. This is shown, for instance, by the lower self-reported PhilHealth membership as compared to the rate claimed by PhilHealth [76].
**Thailand** UCS	Close to 100% (2020) [97]	76% (68,200,000) (2020) [97]	Persons awaiting proof of Thai nationality cannot access UCS until they are granted Thai citizenship. Undocumented migrants who do not enroll in the voluntary migrant health insurance scheme have to pay out-of-pocket [102].
**Vietnam** SHI	No disaggregated data available (SHI has 25 enrolment categories, and IWs can fall into several of them) [56]^b^	90.9% (about 88,000,000) (2020) [96]	Population coverage has remained inequitable despite compulsory enrolment. While enrolment rates of low- and high-income groups are high, coverage of the “missing middle” (i.e. the near-poor and the non-poor, particularly the self-employed and small enterprise owners) has remained low. Additionally, substantial delays in issuing health insurance cards and a lack of communication campaigns have been inhibiting citizens’ access to entitlements in mountainous areas. However, the exact group composition of the currently unenrolled is unknown because there is neither a connection between the SHI and their taxation number nor their national identity number [56, 74, 85, 94, 105].

Abbreviations: HEF = Health Equity Fund; HF = health financing; IW = informal worker; JKN = Jaminan Kesehatan Nasional; NHI = National health Insurance; NHIP = National Health Insurance Program; NHS = National Health Service; SHI = Social Health Insurance; SSS = Social Security Scheme; UCS = Universal Coverage Scheme; UHC = universal health coverage.

^a^ The Cambodian HEF extension is only aimed at covering approximately 8.3% of working-age adults.

^b^ No such data was found in the literature searches. However, such data might exist in unpublished form or published in languages other than English.

Another indicator considers the share of registered IWs compared to those who are eligible for registration, as per the legal provisions of the HF scheme, though data on this indicator is not available for all schemes. Resulting from their universalist approaches, Malaysia’s NHS and the Thai UCS are the only HF schemes with 100% registration of IWs [52, 71]. Estimations suggest that enrolment has remained low in Cambodia’s HEF extension (5.12%, 2020) and is negligible in Myanmar [60, 100, 101].

A vital question to consider is which IW groups have remained without coverage under the analyzed HF schemes, and more broadly in the eight countries. Malaysia and Thailand again have the lowest share of uncovered IW groups. In Thailand, only persons awaiting proof of Thai nationality and undocumented migrants are currently outside the purview of UCS [102]. In Cambodia, Indonesia, the Philippines, and Vietnam, the “missing middle” issue has materialized, with near-poor and non-poor IWs making up the bulk of the currently uncovered groups [56, 62, 65, 66, 74, 85, 94, 103–105]. This issue is particularly acute in Cambodia, where currently only several small IW groups are eligible for the HEF extension [58], while remaining IW groups have remained without coverage [59].

#### 3.7.2 Financial protection

Table 7 illustrates data on OOPE, catastrophic health expenditure, and impoverishment across the whole population and summarizes changes in these indicators and related equity considerations. Excepting Thailand at 11.01%, the share of OOPE of current health expenditure lies at 35% and above; in Cambodia, Myanmar, and the Philippines, over 50% of current health expenditure comprises of OOPE [43]. Data on catastrophic and impoverishing expenditure vary across countries. The percentage of households experiencing catastrophic spending (at 25% of household expenditure) ranges from 0% (Lao PDR, Malaysia, Thailand) to 5% in Cambodia and the incidence of impoverishment lies between 0% in Thailand and 3% in Cambodia [43]. The highest incidence of catastrophic expenditure tends to be found in countries with higher OOP as a share of current health expenditure.

**Table 7:** Financial protection indicators and related changes under the HF schemes.

Country, HF scheme	OOPE as % of THE (2018) [43]	Incidence of CHE (10%, 25% of THCE) [43]	Incidence of IHE ($1.9/$3.2 PPP) [43]	Change of OOPE, CHE, and/or IHE for IWs since introduction of HF scheme	Equity considerations with a focus on IWs
**Cambodia** HEF extension	57.5%	15% (2014) 5% (2014)	3% (2009) 6.2% (2009)	Data not available.^b^	The HEF extension covers only a set of specific IW groups, leaving a large share of IWs with no coverage mechanism. The latter are still required to pay health services through OOPE and thus subjected to the risk of CHE and impoverishment.
**Indonesia** JKN	34.9%	3% (2018) 1% (2018)	0.3% (2015) 0.8% (2015)	Despite overall decreases in OOPE since the introduction of JKN, OOPE remain high in all quintiles, including near-poor and non-poor IWs (mainly on drugs) [89]^a^. No significant impact was found on reductions of CHE at a 10%/25% level [114]. Another study reported poverty reductions through JKN [112]^a^.	Exclusion errors make near-poor IWs ineligible for the subsidized group, restricting service access and reducing financial protection [112]^a^. Additionally, Indonesia’s poverty dynamics are fluid, raising questions on ability-to-pay of IWs with incomes just above the poverty line, and on fairness of health financing [125]^a^.
**Lao PDR** NHI	48.5%	3% (2007) 0% (2007)	0.4% (2007) 1% (2007)	NHI was found to generally lower the cost of health service utilization borne out-of-pocket and to ease financial issues for all income quintiles [87].	Adherence to NHI co-payment policy is variable and NHI has not yet fully replaced user fees; OOPE still constitute the largest share of THE since a major part of the population still pays more than the defined co-payments [91]^a^. Presence of elderly and chronic diseases in HHs is associated with an increased likelihood of experiencing CHE [87, 126]. Lower-income quintiles and lower-educated individuals face an increased risk of CHE when accessing services in Thailand [126].
**Malaysia** NHS	35.1%	1% (2004) 0% (2004)	0.1% (2004) 0.2% (2004)	OOPE have been increasing steadily over the past years, both as a share of THE and in absolute terms [52, 71]^a^.	The general distribution of public health expenditures is pro-poor; expenditures are quite evenly distributed across the four lower quintiles and concentrated in the richest quintile. However, owing to understaffing, long waits, and other supply-side constraints, many people revert to private services, paying out-of-pocket. Thus, with public and private expenditures combined, the overall expenditure distribution is pro-rich. OOPE and CHE remain largely concentrated in higher-income groups opting out of the public system by paying privately, reflecting a lack of purchasing power to access private services among lower-income groups such as IWs [52, 71]^a^.
**Myanmar** SSS	76.4%	14% (2015) 3% (2015)	0.6% (2015) 2.9% (2015)	Data not available; enrolment of IWs negligible.^b^	The general population (largely IWs) reported significantly higher OOPE than SSS members, and greatly increased amounts when borrowing to cover health expenditure and when selling assets. Individuals using specialized care or inpatient services reported an increased likelihood to borrow money or sell assets to cover OOPE (significantly more women than men) [127].
**Philippines** NHIP	53.9%	6% (2015) 1% (2015)	0.5% (2015) 1.4% (2015)	The share of OOPE on household capacity to pay, disposable income, per capita income and expenditure, and non-food expenditure has been increasing. Non-prescription drugs and medical products account for the largest share of OOPE in all income quintiles and are the main driver of CHE [76, 106]^a^.	The share of health services utilized by IWs (22%) exceeds their amount of contributions (5% of total), while private sector members receive fewer benefits (15%) relative to their contributions (51% of total); this cross-subsidization embodies risk-sharing and solidarity from a UHC perspective [66, 81, 124]^a^. However, cross-subsidies from the premium subsidies for indigents and near-poor sponsored IW members to non-poor IWs have been recorded, indicating unintended subsidies from poorer to less poor individuals [128]^a^. Further, sponsored near-poor IWs were found to pay co-payments despite the no-balance billing policy, raising questions on the extent of de facto financial protection for this group (even if their co-payments were lower than those of other groups) [76, 129, 130]^a^.
**Thailand** UCS	11.0%	2% (2017) 0% (2017)	0% (2017) 0% (2017)	After the UCS introduction and UHC achievement, OOPE have been steadily and significantly declining, and CHE and impoverishment due to OOPE dropped markedly. This was largely driven by the coverage expansion of IWs [95, 97, 107, 108].	Health financing is progressive with the rich contributing disproportionately more than the poor [95, 97, 107, 108]. However, onset of a health issue is associated with increases in households’ THE, OOPE, and the percentage incurring CHE compared to the baseline, with effects increasing with the severity of the illness. The probability of borrowing, the amount borrowed and the probability of having a non-labor source of income also increased [131].
**Vietnam** SHI	44.9%	9% (2016) 2% (2016)	0.3% (2016) 1.2% (2016)	OOPE dropped significantly with coverage increases. Insured IWs face significantly lower risks of experiencing CHE than uninsured IWs [109–111]^a^. OOPE are mainly paid by richer quintiles at provincial and central hospitals [109]^a^.	Given the lower utilization by the poor and near-poor compared to the rich, these groups account for a greater share of revenue than expenditure and they subsidize other groups [109]^a^. Households enrolled in SHI still face significant risks of CHE (40% capacity to pay level). Risks increase for households in lower income quintiles, with more severe illness, age, unemployment, and a lower education status of the household head [111].

Abbreviations: CHE = catastrophic health expenditure; HEF = Health Equity Fund; HH = household; IW = informal worker; JKN = Jaminan Kesehatan Nasional; NHI = National Health Insurance; NHIP = National Health Insurance Program; NHS = National Health Service; OOPE = out-of-pocket expenditure; PPP = purchasing power parity; SHI = Social Health Insurance; SSB = Social Security Scheme; THCE = total household consumption expenditure; THE = total health expenditure; UCS = Universal Coverage Scheme.

^a^ Data was partly taken from grey literature publications, the quality of which was not appraised.

^b^ No such data was found in the literature searches. However, such data might exist in unpublished form or published in languages other than English.

Studies examining the impact of HF schemes on financial protection for IWs were relatively scarce with mixed but overall positive results. Most used OOPE or the indicators catastrophic expenditure and impoverishment based on OOPE as main outcome measures. Several studies extended their analyses beyond OOPE and analyzed, for example, coping strategies (e.g. incidences of borrowing, selling assets, or reduction in essential consumption). Excepting Malaysia and the Philippines, where OOPE have been continuously increasing over the past years [52, 71, 76, 106], OOPE have overall decreased through the introduction of the HF schemes for which such data were available (Indonesia, Lao PDR, Thailand, Vietnam) (Table 7). This generally coincided with declines in the incidence of catastrophic and impoverishing spending in these countries, subject to data availability [62, 87, 89, 95, 97, 107–112]. The most pronounced and unambiguous reductions in OOPE and related financial protection indicators were documented for members in the Thai UCS, largely driven by coverage expansion of IWs [95, 97, 107, 108].

#### 3.7.3 Health service utilization

Most articles reported on measures of utilization of outpatient and inpatient health services in general, with only one study looking at indicators for specific health conditions [113]. Outcome measures differed, with analyses conducted using, inter alia, the probability of using care and/or accessibility to care in case of need, utilization within the past one to twelve months, likelihood of accessing different types of providers, and utilization rates (frequency of medical contacts at different care levels). Overall, studies reported mostly favorable results of the HF schemes’ effects on utilization rates: increases in per capita service utilization rates by near-poor and non-poor IWs were observed after the introduction of the schemes in Indonesia, Lao PDR, the Philippines, Thailand, and Vietnam; this generally applied to outpatient and inpatient care [65, 70, 76, 82, 91, 95, 103, 108, 109, 114–118]. Additionally, shifts from uncovered/unregulated providers and from private to public sources of care were observed in Thailand [95, 108]. Notwithstanding these positive effects, closer consideration of IWs’ utilization rates of health services shows inequities for several HF schemes and/or countries. In Indonesia and the Philippines, utilization rates of non-poor IWs are higher compared to other member categories, indicating risks of adverse selection in both schemes [66, 81, 103, 104]. Further, in Lao PDR and Vietnam, utilization rates among IWs continue to lag behind, suggesting that root causes for inequities in access to health services have persisted despite the introduction of new HF schemes [70, 85, 109, 119]. See Table 9 in S1 File for additional details on utilization and related equity considerations.

### 3.8 Factors for enrolment/registration

Various articles analyzed factors influencing IWs’ enrolment/registration and retention (i.e. sustainability of insurance premium payments) in the HF schemes, more specifically HISs. While the exact factors differed to some degree between articles as a function of differing country contexts and HF scheme characteristics, several recurring factors positively influencing enrolment and retention were identified. Among these were older age and improved education of the insured person and the household head, living in urban areas, smaller household size, poorer health status and increased likelihood of being ill a greater number of times in the past year, higher household income and income stability, perception of premiums as acceptable, perceived supply-side readiness of public facilities (e.g. higher perception of quality, reasonable and effectively delivered HBP, availability of public facilities in proximity), enhanced insurance awareness and literacy, and increased convenience of enrolment/registration, payment, claims management, and referral procedures [68, 104, 114, 117, 120–123]. Lastly, one study reported technical issues such as payment channels, payment system challenges, and the flexibility of payment arrangements to be factors impacting regular premium payment by IWs in Indonesia [104]. Further details are given in Table 7 in S1 File.

## 4 Discussion

In this systematic review, we assessed and synthesized peer-reviewed and grey literature publications reporting details on the HF arrangements LMICs in SEA implemented with the aim of extending UHC to near-poor and non-poor IWs. Results from the included publications suggest that explicit commitments to UHC have strong positive effects on population coverage, while voluntary modes of participation and reliance on active enrolment and/or registration of specific population groups seems unfavorable if rapid coverage expansions are to be achieved. Regarding data on progress of the UHC indicators financial protection and access to health services (utilization), available evidence showed overall positive effects since the introduction of the analyzed HF schemes with reductions in OOPE, catastrophic health expenditure and impoverishment, and increases in utilization rates for IWs. Notwithstanding these positive results for several of the analyzed HF schemes, there is relatively scant evidence that provides a broad indication of trends that warrant a conservative interpretation.

The following sections explore the available evidence in more detail and provide reflections on possible reasons for and the further significance of the review findings for the extension of UHC to IWs in LMICs both regionally and globally. We also consider the influence of specific institutional features of the analyzed HF schemes on UHC progress and challenges in the context of high labor force informality.

### 4.1 Relevance of findings

Overall, country evidence suggests that schemes—and countries—with explicit commitments to achieving UHC that adopted universalist approaches have reached the highest population coverage of IWs. Coverage rates varied across HF schemes and coverage data disaggregated by population groups were not available for all schemes. In schemes reaching below 100% coverage, the composition of the unenrolled groups was generally unknown, though IWs in lower middle-income groups were expected to make up the bulk of the uncovered people in several countries (e.g. Philippines, Indonesia) [103, 112, 124]. One explanation identifies the difficulty many countries are facing in identifying IWs, which complicates enrolment and registration under HF schemes [5]. In Thailand, mandatory computerized registration of births and deaths and assigning a unique citizen identifier number greatly facilitated the identification of IWs and the swift implementation of the UCS [95]. Establishing and/or strengthening similar systems in the remaining countries hence appears advisable to accelerate the extension of UHC to uncovered IWs and their families.

The included evidence indicates that enrolment and registration procedures bear influence on both speed and percentage of population coverage under any HF scheme. The highest rate of currently uncovered population groups was found in HF schemes that rely on active enrolment and/or registration of specific population groups, rather than employing a universalist approach. Active enrolment and registration by individuals requires awareness of the HF scheme and their eligibility status, as well as the literacy and competence to navigate the administrative processes, which might not be the case for IWs [21]. However, automatic procedures do not necessarily mean that individuals are cognizant of their coverage status and service entitlements, and do not necessarily translate into service use as indicated by evidence from Lao PDR, the Philippines, and Vietnam [66, 67, 69, 76, 85, 91, 94]. Automatic enrolment and, ideally, registration procedures thus seem to facilitate rapid increases in coverage of previously uninsured groups. This confirms the findings of a previous review in Asian countries [21] and is also consistent with the fact that increased convenience of enrolment and registration and awareness of the HF scheme were two of the most frequently recurring factors for enrolment/registration and retention suggested by the studies included in this review. Indonesia and Thailand have implemented compromise solutions that may be of interest to other countries. Indonesia launched a mobile app in 2017 for IWs and other key population groups to register with JKN and has seen promising increases in the number of monthly registration transactions [73]. In Thailand, beneficiaries can register at health facilities while seeking health services [77].

Furthermore, in schemes with (*de jure* or *de facto)* voluntary enrolment due to enforcement challenges, adverse selection may result as evidenced in Indonesia, the Philippines, and Vietnam; disease history reportedly influenced beneficiaries to enroll and maintain an active enrolment status with regular contribution payments, while fewer illness episodes and low insurance utilization increased premium dropout rates [49, 56, 57, 66, 81]. To address this issue, a possibility for countries aiming to extend UHC to IWs is to design improved policies that enforce enrolment and retention compliance through administrative penalties [132]. Importantly, the technical issues found to influence retention and contribution payment sustainability in Indonesia point to the importance of developing more diversified policies for IWs to cater for their frequently irregular incomes and other special needs [104].

Publications reported mixed results regarding the analyzed financial protection indicators, though with overall significantly positive trends. Most studies reported decreases in OOPE through the introduction of the HF schemes, generally associated with reductions in the incidence of catastrophic and impoverishing spending. Considering the available financial protection data, OOPE have remained high as a share of current health expenditure in most countries as a combined effect of gaps in the scope, breadth, and depth of coverage, with values clearly exceeding the recommended maximum range of 15 to 20% of current health expenditure [42, 133]. This confirms global evidence showing strong correlations between the share of OOPE of current health expenditure and the proportion of households experiencing financial catastrophe [134]. OOPE are also generally considered a suboptimal and inefficient source of health financing since they do not allow for consumption smoothing or risk pooling and redistribution in healthcare [25, 71]. Hence, even in countries where OOPE are currently concentrated among richer quintiles and do not seem to severely jeopardize financial protection (e.g. Vietnam), reducing such expenditures and transitioning to more sustainable and increasingly prepaid, pooled financing sources provides a major opportunity for further health financing reform in countries aiming to make progress toward UHC while grappling with high levels of informality. In this context, it is important to note that the low OOPE among poorer quintiles could also be a result of low service access [109].

The design of the reimbursement system is another essential aspect that should be considered by countries aiming to extend coverage to IWs since it may influence financial protection and create or reduce financial barriers to care for this group. The review showed that the extent of subsidization and the amount of co-payments IWs are obligated to pay are directly connected to the share of OOPE, and thus the level of financial protection. The Thai UCS, which was found to improve financial protection for IWs most effectively among the analyzed HF schemes, provides full subsidies to IWs and a comprehensive set of services that patients can access free of charge [95]. In most of the remaining HF schemes, IWs are not subsidized or only partially subsidized and required to co-pay for the care they receive. This may be particularly difficult for IWs in lower-income groups who are both most in need of care and prone to forego treatment [21]. In the Philippines patients need to advance treatment costs and get reimbursed based on insurance claims submitted to PhilHealth [76, 106], while in the remaining HF schemes, health services are provided without direct payment (besides co-payments if applicable). IWs, with often low and insecure incomes, may be unable to advance costs, and an average lower education level may also impact their ability to submit claims [135]. This type of reimbursement system may therefore constitute an access barrier and help explain the fact that overall PhilHealth benefit utilization has remained low among lower socioeconomic quintiles [76].

Furthermore, applying uniform levels of subsidization and co-payments to the generally heterogeneous group of IWs may not reflect within-group differences in affordability and introduce or exacerbate inequity in health financing [136]. Poverty is fluid and dynamic with IWs generally experiencing high levels of vulnerability due to episodes of poverty at certain points in their lives; income gradients between IW groups in terms of their capacity to pay are often shallow [8]. Additionally, poverty and impoverishment indicators react sensitively to the choice of poverty lines [137]. There are therefore no static and easily identifiable population groups such as ‘the poor’ and ‘the non-poor’, or in some countries the trichotomy with ‘the near-poor’ as a third group, reducing targeting effectiveness and increasing chances for inclusion and exclusion errors [138]. Recognizing and addressing this ambiguity in national, regional, and global discussions and around extending UHC to IWs is vital.

The health benefits package is directly connected to the depth of service coverage and affects the degree of financial protection a HF scheme offers to the covered population. In several of the analyzed HF schemes there are differences in what services the HBP sets out to provide to covered IWs (i.e. *de jure*) and the services received by the population in practice (i.e. *de facto*), leading to implicit rationing. This can compromise the government’s trustworthiness and credibility in public health service provision, and is leveraged by private providers trying to attract patients by delivering, for instance, services unavailable in the public sector or reducing waiting times [62, 71]. Seeking care in the private sector may pose financial barriers and increase levels of OOPE, especially for IWs in lower-income quintiles, as shown in Cambodia [139]. This emphasizes that extending UHC to IWs is not possible by merely expanding population coverage, but that successful UHC reform requires concurrent development and improvement of the health system to address supply-side constraints [51]. An example of this is Thailand’s successful transition, where nationwide coverage of PHC with high infrastructure and service readiness provided a sound basis for health financing and system reform before the extension of UCS to uncovered IWs and their families [95].

While the focus of the UHC agenda is on extending prepayment mechanisms to protect against health risks, this review identified data showing that income losses threaten living standards in settings without formal earnings insurance [131]. While this is particularly true for longer, more serious illnesses, even the opportunity costs of spending long hours waiting at health facilities constitute a financial access barrier for IWs who often depend on their daily wages [72]. Jointly considering both risks is therefore essential for countries aiming to strengthen social (health) protection for IWs and their families.

Publications reported overall positive results with increases in utilization rates since the introduction of the analyzed HF schemes for previously uninsured groups of society—including IWs. However, results in several studies were not significant, evidence was sparse or unavailable for IWs for certain HF schemes (e.g. Cambodia’s HEF extension and the Laotian NHI), and the schemes’ effects on utilization were inherently heterogeneous and sensitive to differences in context. Additionally, the extent to which increased utilization by IWs constitutes improvement in welfare depends largely on the level of existing unmet need which is thus filled [18]. Overall, this highlights the challenges in establishing an unambiguous link between HF schemes and outcomes and in providing implementation guidance beyond the contexts where the HF schemes were initially established. Policymakers planning to extend UHC to IWs should critically evaluate the transferability of study results to their contexts and, where necessary, collect context-specific data before taking any decisions.

Governance arrangements and government stewardship warrant mention given their decisive importance for effective health financing policy making and implementation, particularly in the context of high labor force informality [2]. A 2019 study found that Vietnam and Indonesia, two countries with high population coverage—and high levels of informality—score high in government effectiveness indicators [140]. Strengthening the governance environment at both national and decentralized levels is therefore an essential element of countries’ path toward UHC [2]. As shown by Thailand’s experience, rapid and effective implementation of reforms can be achieved amid a favorable political economy with strong government commitment and leadership with efforts to manage and reduce conflict between stakeholders that translates into elevated and sufficient budget allocation levels [95, 97, 107]. Indonesia’s launch of JKN (2014), the revised HIS law in Vietnam (2014), Lao PDR’s landmark NHI (2016), and the UHC Act in the Philippines (2019), as well as the progressive expansion of these HF schemes in the subsequent years, are also a result of strong political will to extend UHC to the entire population, most notably the “missing middle” of IWs and their families. Importantly, the Association of Southeast Asian Nations (ASEAN) supports increasing multilateral collaboration between its member countries. Health has been designated a priority sector of regionwide cooperation [141], which includes for example initiatives such as the regular ASEAN Health Ministers Meeting and the ASEAN Post-2015 Health Development Agenda 2021–2025. The latter prominently features countries’ joint political commitments toward UHC [142]. Moreover, the ASEAN Plus Three UHC Network has influenced national health policy agendas, including urging member countries to extend coverage to currently uncovered groups. In particular, the ASEAN health cooperation also includes the protection and promotion of healthcare delivery to migrant workers [142]–who are often also informal workers—and who have in previous research been shown to lack adequate access to essential health services and financial protection [143]. A 2015 review by Van Minh et al. presents a synoptic view of progress toward UHC in ASEAN countries [144], which future research could build upon and expand to further assess the role of ASEAN membership in influencing UHC reforms.

This review revealed a lack of effective pooling and strategic purchasing mechanisms in most of the analyzed HF schemes, leaving a potential to use information to create deliberate incentives for equitable resource redistribution and efficiency increases unfulfilled [145]. In addition to more funding, country experiences show that successful UHC reforms in fiscal contexts associated with high informality require increased flexibility in how budget revenues can be used, in particular adapting pooling arrangements and funding flows from passive to strategic purchasing techniques [2, 146].

The evidence included in this systematic review does not offer a “best model” for countries aiming to extend UHC to IWs. Nevertheless, a lot can be learned from sharing global, regional, and country-level experience in this field, which suggests a number of desirable directions for reform [2, 18]. Fuchs established two necessary and sufficient conditions for UHC (known as “Fuchs conditions”): compulsion and subsidization for low-income groups [147]. So far, no country has attained UHC through primarily voluntary contributions [148]. This is also evident in the analyzed HF schemes where the lowest population coverage rates were found in the Cambodian HF scheme, offering voluntary enrolment to IWs [58, 59]. Especially since the HEF extension provides full subsidies to IWs [58, 59], this confirms Fuchs’ statement that “subsidies without compulsion will not work” [147]. Vietnam was facing similar challenges with low enrolment rates of IWs in its VHI scheme and has therefore introduced mandatory SHI coverage for the entire population in 2014. Importantly, Vietnam used its VHI as a steppingstone for mandatory enrolment (e.g. open enrolment and community rating of contributions) [56], which could be instructive for other LMICs seeking to realize UHC with larger shares of the population operating in the informal economy. Generally, most countries have achieved coverage expansion to IWs through a noncontributory basis of entitlement, moving toward predominant reliance on general revenues and full subsidies [2]. These approaches have been chosen by both Thailand and Lao PDR. However, this route requires the fiscal space and political will to prioritize health spending, as well as the technical and managerial capacity to control expenditure growth, which might be challenging for many LMICs who have larger shares of the population in the informal economy [2, 18, 27, 85]. Additionally, this approach might encourage further “informalization” and distort labor market choices [85, 149]. Given the constraints in raising revenues from direct taxes in these contexts, choosing this path to meet the Fuchs conditions will likely require making greater use of indirect and/or excise taxes [2]. On the other hand, Indonesia, the Philippines, and Vietnam have taken a contributory road with subsidies for near-poor and unsubsidized coverage for non-poor IWs. While population coverage is above 80% in all three countries, covering the remaining groups has proven challenging as illustrated, for instance, by the continuously decelerating membership growth of IWs in Indonesia’s JKN [103]. This thus corroborates international experiences showing that extending contributory SHI to IWs is very difficult or nearly impossible [2, 18, 27].

### 4.2 Limitations

Notwithstanding the systematic approach in searching for eligible literature, relevant publications may have been omitted. Conducting searches in other electronic databases and/or on other development partner or governmental websites beyond the ones searched, as well as consulting country experts for relevant literature may have yielded additional relevant publications, and the fact that only English-language publications were eligible for this review may have introduced the risk of missing key data due to language bias. Additionally, many studies were excluded because there was no clear-cut distinction between the population groups for which they analyzed the effects of the respective HF schemes. These might have reported on potentially relevant evidence that we consequently missed, though the extent to which additional publications would affect the main results and conclusions of this systematic review is unclear. Furthermore, quality appraisal for non-randomized studies was complicated due to the heterogeneity inherent in this category of study designs. There is currently no consensus on which tool(s) to use for appraising the methodological quality of non-randomized studies [150]. We therefore selected the Joanna Briggs Institute checklists since these provide a set of coherent and comparable tools [35]. Nevertheless, it cannot be ruled out that the use of a different set of checklists might have yielded different results. Moreover, since this study offers a synoptic perspective on multiple HF schemes, details provided on the included country cases are inevitably limited. Lastly, the conclusions on the impact of the HF schemes on population coverage and financial protection for and health service utilization by IWs may be considered general due to the large variety of study designs and indicators used in the included publications as well as the limited evidence found for several HF schemes and/or countries. Nonetheless, our results provide a good indication of trends in these indicators and of the extent to which efforts and approaches aimed at extending UHC to IWs in countries in SEA are represented in the published literature.

## 5 Conclusions

Based on this systematic review it can be concluded that there is relatively limited evidence on changes in the three UHC indicators of population coverage, financial protection, and access to health services for several HF schemes. The overall quality of the peer-reviewed articles was medium with variations in applied study designs and methods. These methodological and data limitations should be addressed by rigorous studies to better understand the relation between efforts to extend UHC to IWs and improvements in key UHC indicators. As a general trend, IW population coverage as well as rates of their financial protection and service utilization increased since the introduction of the HF schemes, though with effects strongly depending on context and features of the respective scheme. Countries adopting HF schemes consistent with international directions on desirable features of reform, i.e. compulsory enrolment and subsidization for lower-income groups, generally show more favorable results.

## Supporting information

S1 File (DOCX)
