# Supplementary material for: Extending universal health coverage to informal workers: A systematic review of health financing schemes in low- and middle-income countries in Southeast Asia
Source: PLoS One. 2023 Jul 11;18(7):e0288269. doi: 10.1371/journal.pone.0288269 (PMC10335706; doi:10.1371/journal.pone.0288269)
Supplement: S1 File — (DOCX) [file pone.0288269.s001.docx]

# **Supporting information**

**Table 1. PRISMA checklist***.*

| **Section and Topic** | **Item #** | **Checklist item** | **Location where item is reported** |
| --- | --- | --- | --- |
| **TITLE** | | |  |
| Title | 1 | Identify the report as a systematic review. | Title |
| **ABSTRACT** | | |  |
| Abstract | 2 | See the PRISMA 2020 for Abstracts checklist. | Abstract |
| **INTRODUCTION** | | |  |
| Rationale | 3 | Describe the rationale for the review in the context of existing knowledge. | 1 Introduction |
| Objectives | 4 | Provide an explicit statement of the objective(s) or question(s) the review addresses. | 1 Introduction |
| **METHODS** | | |  |
| Eligibility criteria | 5 | Specify the inclusion and exclusion criteria for the review and how studies were grouped for the syntheses. | 2.3 Search strategy, study selection, and eligibility criteria |
| Information sources | 6 | Specify all databases, registers, websites, organisations, reference lists and other sources searched or consulted to identify studies. Specify the date when each source was last searched or consulted. | 2.2 Information sources |
| Search strategy | 7 | Present the full search strategies for all databases, registers and websites, including any filters and limits used. | Supplementary information. Tables 2 - 5 |
| Selection process | 8 | Specify the methods used to decide whether a study met the inclusion criteria of the review, including how many reviewers screened each record and each report retrieved, whether they worked independently, and if applicable, details of automation tools used in the process. | 2.3 Search strategy, study selection, and eligibility criteria |
| Data collection process | 9 | Specify the methods used to collect data from reports, including how many reviewers collected data from each report, whether they worked independently, any processes for obtaining or confirming data from study investigators, and if applicable, details of automation tools used in the process. | 2.4 Data extraction and items |
| Data items | 10a | List and define all outcomes for which data were sought. Specify whether all results that were compatible with each outcome domain in each study were sought (e.g. for all measures, time points, analyses), and if not, the methods used to decide which results to collect. | 2.6 Data analysis and synthesis |
|  | 10b | List and define all other variables for which data were sought (e.g. participant and intervention characteristics, funding sources). Describe any assumptions made about any missing or unclear information. | 2.4 Data extraction and items |
| Study risk of bias assessment | 11 | Specify the methods used to assess risk of bias in the included studies, including details of the tool(s) used, how many reviewers assessed each study and whether they worked independently, and if applicable, details of automation tools used in the process. | 2.5 Quality appraisal |
| Effect measures | 12 | Specify for each outcome the effect measure(s) (e.g. risk ratio, mean difference) used in the synthesis or presentation of results. | (2.6 Data analysis and synthesis) |
| Synthesis methods | 13a | Describe the processes used to decide which studies were eligible for each synthesis (e.g. tabulating the study intervention characteristics and comparing against the planned groups for each synthesis (item #5)). | (2.3 Search strategy, study selection, and eligibility criteria) |
|  | 13b | Describe any methods required to prepare the data for presentation or synthesis, such as handling of missing summary statistics, or data conversions. | 2.6 Data analysis and synthesis |
|  | 13c | Describe any methods used to tabulate or visually display results of individual studies and syntheses. | 2.6 Data analysis and synthesis |
|  | 13d | Describe any methods used to synthesize results and provide a rationale for the choice(s). If meta-analysis was performed, describe the model(s), method(s) to identify the presence and extent of statistical heterogeneity, and software package(s) used. | 2.6 Data analysis and synthesis |
|  | 13e | Describe any methods used to explore possible causes of heterogeneity among study results (e.g. subgroup analysis, meta-regression). | Not applicable |
|  | 13f | Describe any sensitivity analyses conducted to assess robustness of the synthesized results. | Not applicable |
| Reporting bias assessment | 14 | Describe any methods used to assess risk of bias due to missing results in a synthesis (arising from reporting biases). | Not applicable |
| Certainty assessment | 15 | Describe any methods used to assess certainty (or confidence) in the body of evidence for an outcome. | Not applicable |
| **RESULTS** | | |  |
| Study selection | 16a | Describe the results of the search and selection process, from the number of records identified in the search to the number of studies included in the review, ideally using a flow diagram. | 3. Results |
|  | 16b | Cite studies that might appear to meet the inclusion criteria, but which were excluded, and explain why they were excluded. | Supplementary information List 2 |
| Study characteristics | 17 | Cite each included study and present its characteristics. | Supplementary information List 1 and Table 7 |
| Risk of bias in studies | 18 | Present assessments of risk of bias for each included study. | Supplementary information Table 7 |
| Results of individual studies | 19 | For all outcomes, present, for each study: (a) summary statistics for each group (where appropriate) and (b) an effect estimate and its precision (e.g. confidence/credible interval), ideally using structured tables or plots. | Not applicable |
| Results of syntheses | 20a | For each synthesis, briefly summarise the characteristics and risk of bias among contributing studies. | Not applicable |
|  | 20b | Present results of all statistical syntheses conducted. If meta-analysis was done, present for each the summary estimate and its precision (e.g. confidence/credible interval) and measures of statistical heterogeneity. If comparing groups, describe the direction of the effect. | Not applicable |
|  | 20c | Present results of all investigations of possible causes of heterogeneity among study results. | Not applicable |
|  | 20d | Present results of all sensitivity analyses conducted to assess the robustness of the synthesized results. | Not applicable |
| Reporting biases | 21 | Present assessments of risk of bias due to missing results (arising from reporting biases) for each synthesis assessed. | Not applicable |
| Certainty of evidence | 22 | Present assessments of certainty (or confidence) in the body of evidence for each outcome assessed. | Not applicable |
| **DISCUSSION** | | |  |
| Discussion | 23a | Provide a general interpretation of the results in the context of other evidence. | 4.1 Relevance of findings |
|  | 23b | Discuss any limitations of the evidence included in the review. | 4.1 Relevance of findings |
|  | 23c | Discuss any limitations of the review processes used. | 4.2 Limitations |
|  | 23d | Discuss implications of the results for practice, policy, and future research. | 4.1 Relevance of findings |
| **OTHER INFORMATION** | | |  |
| Registration and protocol | 24a | Provide registration information for the review, including register name and registration number, or state that the review was not registered. | 2.1 Protocol and registration |
|  | 24b | Indicate where the review protocol can be accessed, or state that a protocol was not prepared. | 2.1 Protocol and registration |
|  | 24c | Describe and explain any amendments to information provided at registration or in the protocol. | Not applicable |
| Support | 25 | Describe sources of financial or non-financial support for the review, and the role of the funders or sponsors in the review. | 5. Acknowledgements |
| Competing interests | 26 | Declare any competing interests of review authors. | 5. Acknowledgements |
| Availability of data, code and other materials | 27 | Report which of the following are publicly available and where they can be found: template data collection forms; data extracted from included studies; data used for all analyses; analytic code; any other materials used in the review. | Available upon request to the authors |

From*:*  Page MJ, McKenzie JE, Bossuyt PM, Boutron I, Hoffmann TC, Mulrow CD, et al. The PRISMA 2020 statement: an updated guideline for reporting systematic reviews. BMJ 2021;372:n71. doi: 10.1136/bmj.n71

**Search strategies**

**Date of searches (all databases)**: April 29, 2021

**Date of coverage (all databases)**: January 01, 2010 to April 29, 2021

**Table 2. Search strategy for MEDLINE (PubMed user interface).**

| **No** | **Search strategy** | **Hits** | **Search strategy translation** |
| --- | --- | --- | --- |
| 1 | ("Universal health coverage" OR UHC OR "health coverage" OR "universal coverage" OR "national health insurance" OR NHI OR "social health insurance" OR SHI OR "private health insurance" OR "community-based health insurance" OR CBHI OR "community insurance” OR “voluntary health insurance" OR “voluntary insurance” OR "tax-based financing" OR "tax based financing" OR "tax financing" OR "health financing" OR "healthcare financing”) | 202762 | "Universal health coverage"[All Fields] OR "UHC"[All Fields] OR "health coverage"[All Fields] OR "universal coverage"[All Fields] OR "national health insurance"[All Fields] OR "NHI"[All Fields] OR "social health insurance"[All Fields] OR "SHI"[All Fields] OR "private health insurance"[All Fields] OR "community-based health insurance"[All Fields] OR "CBHI"[All Fields] OR "community insurance"[All Fields] OR "voluntary health insurance"[All Fields] OR "voluntary insurance"[All Fields] OR "tax-based financing"[All Fields] OR "tax-based financing"[All Fields] OR "tax financing"[All Fields] OR "health financing"[All Fields] OR "healthcare financing"[All Fields] |
| 2 | ("population coverage" OR "health coverage" OR "health insurance coverage" OR "financial protection" OR "financial risk protection" OR "catastrophic expenditure" OR "catastrophic health expenditure" OR "catastrophic effects" OR "impoverishing effects" OR "out-of-pocket expenditure" OR "out-of-pocket expenses" OR "out-of-pocket payments" OR "service access" OR "impoverishing health expenditure" OR "impoverishing expenditure" OR "access to services" OR "access to health services" OR "access to care" OR "access to healthcare" OR "access to health care" OR "health service utilization" OR "service utilization" OR "service coverage" OR "universal access") | 43300 | "population coverage"[All Fields] OR "health coverage"[All Fields] OR "health insurance coverage"[All Fields] OR "financial protection"[All Fields] OR "financial risk protection"[All Fields] OR "catastrophic expenditure"[All Fields] OR "catastrophic health expenditure"[All Fields] OR "catastrophic effects"[All Fields] OR "impoverishing effects"[All Fields] OR "out-of-pocket expenditure"[All Fields] OR "out-of-pocket expenses"[All Fields] OR "out-of-pocket payments"[All Fields] OR "service access"[All Fields] OR "impoverishing health expenditure"[All Fields] OR "impoverishing expenditure"[All Fields] OR "access to services"[All Fields] OR "access to health services"[All Fields] OR "access to care"[All Fields] OR "access to healthcare"[All Fields] OR "access to health care"[All Fields] OR "health service utilization"[All Fields] OR "service utilization"[All Fields] OR "service coverage"[All Fields] OR "universal access"[All Fields] |
| 3 | (universal health insurance[MeSH Terms]) OR (insurance, health[MeSH Terms]) | 153434 | universal health insurance[MeSH Terms]: "universal health insurance"[MeSH Terms] insurance, health[MeSH Terms]: "insurance, health"[MeSH Terms] |
| 4 | #1 OR #2 OR #3 | 381072 |  |
| 5 | “asia, southeastern"[MeSH Terms] | 101711 | asia, southeastern[MeSH Terms]: "asia, southeastern"[MeSH Terms] |
| 6 | "Southeast Asia" OR "South-east Asia" OR "South east Asia" OR Myanmar OR Burma OR Cambodia OR Indonesia OR Laos OR "Lao PDR" OR Malaysia OR Philippines OR Thailand OR Vietnam OR “Viet Nam” | 253304 | Brunei: "brunei"[MeSH Terms] OR "brunei"[All Fields] Myanmar: "myanmar"[MeSH Terms] OR "myanmar"[All Fields] OR "myanmar's"[All Fields] OR "myanmars"[All Fields] Burma: "burma's"[All Fields] OR "myanmar"[MeSH Terms] OR "myanmar"[All Fields] OR "burma"[All Fields] Cambodia: "cambodia"[MeSH Terms] OR "cambodia"[All Fields] OR "cambodia's"[All Fields] Indonesia: "indonesia"[MeSH Terms] OR "indonesia"[All Fields] OR "indonesia's"[All Fields] OR "indonesias"[All Fields] Laos: "laos"[MeSH Terms] OR "laos"[All Fields] Malaysia: "malaysia"[MeSH Terms] OR "malaysia"[All Fields] OR "malaysia's"[All Fields] Philippines: "philippine"[All Fields] OR "philippines"[MeSH Terms] OR "philippines"[All Fields] Singapore: "singapore"[MeSH Terms] OR "singapore"[All Fields] OR "singapore's"[All Fields] Thailand: "thailand"[MeSH Terms] OR "thailand"[All Fields] OR "thailand's"[All Fields] Vietnam: "vietnam"[MeSH Terms] OR "vietnam"[All Fields] OR "vietnam's"[All Fields] |
| 7 | #5 OR #6 | 269560 | - |
| 8 | #3 AND #7 | 3476 | - |
| 9 | Filter 2010 - present | 2935 | - |

**Table 3. Search strategy for Cochrane Library.**

| **No** | **Search strategy** | **Hits** |
| --- | --- | --- |
| 1 | Universal Health Coverage OR universal coverage OR universal health | 2466 |
| 2 | MeSH descriptor: [Universal Health Insurance] explode all trees | 5 |
| 3 | #1 OR #2 | 2466 |
| 4 | "Southeast Asia" OR "South-east Asia" OR "South east Asia" OR Myanmar OR Burma OR Cambodia OR Indonesia OR Laos OR "Lao PDR" OR Malaysia OR Philippines OR Singapore OR Thailand OR Vietnam | 19252 |
| 5 | MeSH descriptor: [Asia, Southeastern] explode all trees | 2657 |
| 6 | #4 OR #5 | 19271 |
| 7 | #3 AND #6 | 177 |
| 8 | Filter 2010 - present | 171 |

**Table 4. Search strategy for JSTOR.**

| **No** | **Search strategy** | **Hits** |
| --- | --- | --- |
| 1 | ((("Universal health coverage")) | NA |
| 2 | AND (Brunei OR Myanmar OR Burma OR Cambodia OR Indonesia OR Laos OR "Lao PDR" OR Malaysia OR Philippines OR Thailand OR "Timor-Leste" OR Vietnam)) | NA |
| 3 | AND la:(eng OR en) | 575 |

**Table 5. Search strategy for Web of Science.**

| **No** | **Search strategy** | **Hits** |
| --- | --- | --- |
| 1 | ("Universal health coverage" OR UHC OR "health coverage" OR "universal coverage" OR "national health insurance" OR NHI OR "social health insurance" OR SHI OR "private health insurance" OR "community-based health insurance" OR CBHI OR "voluntary health insurance" OR "tax-based financing" OR "tax based financing" OR "tax financing" OR "health financing" OR "healthcare financing") | NA |
| 2 | "Southeast Asia" OR "South-east Asia" OR "South east Asia" OR Myanmar OR Burma OR Cambodia OR Indonesia OR Laos OR "Lao PDR" OR Malaysia OR Philippines OR Thailand OR Vietnam | NA |
| 3 | #1 OR #2 | 452 |

**Table 6. Exclusion criteria.**

| **Component** | **Exclusion criteria** |
| --- | --- |
| P - Population | Studies analyzing populations other than near-poor and non-poor informal workers, or studies in which the reported data is not clearly attributable to near-poor or non-poor informal workers. |
| I - Intervention | HF schemes not aimed at UHC (e.g. vertical, disease-specific schemes, voucher schemes, or conditional cash transfers). Proposed HF schemes (i.e. the scheme was not yet implemented in one of the study countries) |
| C - Control | Not applicable. |
| O - Outcome | Outcomes other than the UHC indicators population coverage, financial protection, and access to essential health services (utilization). |
| S - Study design | Commentaries, editorials, opinion pieces, policy briefs, perspectives, conference abstracts, letters to the editor, or proposals. |
| Other - Language | Publications reported in languages other than English. |
| Other - Timeframe | Publications published before 2010 (coincident with the publication of the World Health Report ‘Health systems financing: the path to universal coverage’). |
| Other – Setting | Publications reporting on data for countries other than the included low- and middle-income countries in Southeast Asia. |
| Other - Data access | Publications for which the full text could not be accessed. |

**List 1. Included peer-reviewed articles and grey literature publications (in alphabetical order according to country).**

**A Peer-reviewed articles**

**Cambodia**

1. Annear PL, Ahmed S, Ros CE, Ir P. Strengthening institutional and organizational capacity for social health protection of the informal sector in lesser-developed countries: a study of policy barriers and opportunities in Cambodia. Soc Sci Med. 2013;96: 223–231. doi:10.1016/j.socscimed.2013.02.015
2. Ozawa S, Grewal S, Bridges JFP. Household Size and the Decision to Purchase Health Insurance in Cambodia: Results of a Discrete-Choice Experiment with Scale Adjustment. Appl Health Econ Health Policy. 2016;14: 195–204. doi:10.1007/s40258-016-0222-9

**Indonesia**

1. Agustina R, Dartanto T, Sitompul R, Susiloretni KA, Suparmi, Achadi EL, et al. Universal health coverage in Indonesia: concept, progress, and challenges. Lancet (London, England). 2019;393: 75–102. doi:10.1016/S0140-6736(18)31647-7
2. Dartanto T, Halimatussadiah A, Rezki JF, Nurhasana R, Siregar CH, Bintara H, et al. Why Do Informal Sector Workers Not Pay the Premium Regularly? Evidence from the National Health Insurance System in Indonesia. Appl Health Econ Health Policy. 2020;18: 81–96. doi:10.1007/s40258-019-00518-y
3. Dartanto T, Rezki JF, Pramono W, Siregar CH, Bintara U, Bintara H. Participation of Informal Sector Workers in Indonesia’s National Health Insurance System. J Southeast Asian Econ. 2016;33: 317–342. Available: http://www.jstor.org/stable/44132409
4. Dartanto T, Pramono W, Lumbanraja AU, Siregar CH, Bintara H, Sholihah NK, et al. Enrolment of informal sector workers in the National Health Insurance System in Indonesia: A qualitative study. Heliyon. 2020;6: e05316. doi:10.1016/j.heliyon.2020.e05316
5. Erlangga D, Ali S, Bloor K. The impact of public health insurance on healthcare utilisation in Indonesia: evidence from panel data. Int J Public Health. 2019;64: 603–613. doi:10.1007/s00038-019-01215-2
6. Rahmadani S, Marhani, Abadi MY, Marzuki DS, Sudirman, Fajrin M Al. Analysis of independent National Health Insurance ownership of informal workers: Study of market traders in Gowa District, Indonesia. Enferm Clin. 2020;30 Suppl 6: 295–299. doi:10.1016/j.enfcli.2020.06.076
7. Sparrow R, Budiyati S, Yumna A, Warda N, Suryahadi A, Bedi AS. Sub-national health care financing reforms in Indonesia. Health Policy Plan. 2017;32: 91–101. doi:10.1093/heapol/czw101
8. Sparrow R, Suryahadi A, Widyanti W. Social health insurance for the poor: targeting and impact of Indonesia’s Askeskin programme. Soc Sci Med. 2013;96: 264–271. doi:10.1016/j.socscimed.2012.09.043

**Lao PDR**

1. Alkenbrack S, Jacobs B, Lindelow M. Achieving universal health coverage through voluntary insurance: what can we learn from the experience of Lao PDR? BMC Health Serv Res. 2013;13: 521. doi:10.1186/1472-6963-13-521
2. Alkenbrack S, Lindelow M. The impact of community-based health insurance on utilization and out-of-pocket expenditures in Lao People’s Democratic Republic. Health Econ. 2013;24: 379–399. doi:10.1002/hec.3023
3. Bodhisane S, Pongpanich S. The impact of National Health Insurance upon accessibility of health services and financial protection from catastrophic health expenditure: a case study of Savannakhet province, the Lao People’s Democratic Republic. Heal Res policy Syst. 2019;17: 99. doi:10.1186/s12961-019-0493-3
4. Bodhisane S, Pongpanich S. The Impact of Community Based Health Insurance in Enhancing Better Accessibility and Lowering the Chance of Having Financial Catastrophe Due to Health Service Utilization: A Case Study of Savannakhet Province, Laos. Int J Health Serv. 2017;47: 504–518. doi:10.1177/0020731415595609
5. Bodhisane S, Pongpanich S. Factors affecting the willingness to join community-based health insurance (CBHI) scheme: A case study survey from Savannakhet Province, Lao P.D.R. Int J Health Plann Manage. 2019;34: 604–618. doi:10.1002/hpm.2721
6. Bodhisane S, Pongpanich S. The accessibility and probability of encountering catastrophic health expenditure by Lao patients in Thai hospitals. J Public Health (Oxf). 2021. doi:10.1093/pubmed/fdab043
7. Chaleunvong K, Phoummalaysith B, Phonvixay B, Sychareun V, Durham J, Essink DR. Factors affecting knowledge of National Health Insurance Policy among out-patients in Lao PDR: an exit interview study. Glob Health Action. 2020;13: 1791414. doi:10.1080/16549716.2020.1791414
8. Chaleunvong K, Phoummalaysith B, Phonvixay B, Vonglokham M, Sychareun V, Durham J, et al. Factors associated with patient payments exceeding National Health Insurance fees and out-of-pocket payments in Lao PDR. Glob Health Action. 2020;13: 1791411. doi:10.1080/16549716.2020.1791411
9. Sydavong T, Goto D, Kawata K, Kaneko S, Ichihashi M. Potential demand for voluntary community-based health insurance improvement in rural Lao People’s Democratic Republic: A randomized conjoint experiment. PLoS One. 2019;14: e0210355. doi:10.1371/journal.pone.0210355

**Malaysia**

1. Balqis-Ali NZ, Anis-Syakira J, Fun WH, Sararaks S. Private Health Insurance in Malaysia: Who Is Left Behind? Asia-Pacific J public Heal. 2021; 10105395211000912. doi:10.1177/10105395211000913

**Myanmar**

1. Myint C-Y, Pavlova M, Groot W. Health insurance in Myanmar: Knowledge, perceptions, and preferences of Social Security Scheme members and general adult population. Int J Health Plann Manage. 2019;34: 346–369. doi:10.1002/hpm.2643
2. Myint C-Y, Pavlova M, Groot W. Patterns of health care use and out-of-pocket payments among general population and social security beneficiaries in Myanmar. BMC Health Serv Res. 2019;19: 258. doi:10.1186/s12913-019-4071-8

**The Philippines**

1. Capuno JJ, Kraft AD, Quimbo S, Tan CRJ, Wagstaff A. Effects of Price, Information, and Transactions Cost Interventions to Raise Voluntary Enrollment in a Social Health Insurance Scheme: A Randomized Experiment in the Philippines. Health Econ. 2016;25: 650–662. doi:10.1002/hec.3291
2. Obermann K, Jowett M, Kwon S. The role of national health insurance for achieving UHC in the Philippines: a mixed methods analysis. Glob Health Action. 2018;11: 1483638. doi:10.1080/16549716.2018.1483638
3. Querri A, Ohkado A, Kawatsu L, Remonte MA, Medina A, Garfin AMC. The challenges of the Philippines’ social health insurance programme in the era of Universal Health Coverage. Public Heal action. 2018;8: 175–180. doi:10.5588/pha.18.0046
4. Tobe M, Stickley A, del Rosario RBJ, Shibuya K. Out-of-pocket medical expenses for inpatient care among beneficiaries of the National Health Insurance Program in the Philippines. Health Policy Plan. 2013;28: 536–548. doi:10.1093/heapol/czs092

**Thailand**

1. Kirdruang P, Glewwe P. The Impact of Universal Health Coverage on Households’ Consumption and Savings in Thailand. J Asia Pacific Econ. 2018;23: 78–98. doi:10.1080/13547860.2017.1359893
2. Limwattananon S, Neelsen S, O’Donnell O, Prakongsai P, Tangcharoensathien V, van Doorslaer E, et al. Universal coverage with supply-side reform: The impact on medical expenditure risk and utilization in Thailand. J Public Econ. 2015;121.
3. Limwattananon S, Tangcharoensathien V, Tisayaticom K, Boonyapaisarncharoen T, Prakongsai P. Why has the Universal Coverage Scheme in Thailand achieved a pro-poor public subsidy for health care? BMC public health. 2012. p. S6. doi:10.1186/1471-2458-12-S1-S6
4. Meemon N, Paek SC. Analysis of Composition Change of Public Facility Care Users After the Universal Coverage Scheme in Thailand. SAGE Open. 2020;10: 2158244020947423. doi:10.1177/2158244020947423
5. Neelsen S, Limwattananon S, O’Donnell O, van Doorslaer E. Universal health coverage: A (social insurance) job half done? World Dev. 2019;113: 246–258. doi:https://doi.org/10.1016/j.worlddev.2018.09.004
6. Patcharanarumol W, Panichkriangkrai W, Sommanuttaweechai A, Hanson K, Wanwong Y, Tangcharoensathien V. Strategic purchasing and health system efficiency: A comparison of two financing schemes in Thailand. PLoS One. 2018;13: e0195179. doi:10.1371/journal.pone.0195179
7. Suriyawongpaisal P, Aekplakorn W, Tansirisithikul R. Does harmonization of payment mechanisms enhance equitable health outcomes in delivery of emergency medical services in Thailand? Health Policy Plan. 2015;30: 1342–1349. doi:10.1093/heapol/czv005
8. Tangcharoensathien V, Limwattananon S, Patcharanarumol W, Thammatacharee J, Jongudomsuk P, Sirilak S. Achieving universal health coverage goals in Thailand: the vital role of strategic purchasing. Health Policy Plan. 2015;30: 1152–1161. doi:10.1093/heapol/czu120
9. Tangcharoensathien V, Patcharanarumol W, Kulthanmanusorn A, Saengruang N, Kosiyaporn H. The Political Economy of UHC Reform in Thailand: Lessons for Low- and Middle-Income Countries. Heal Syst reform. 2019;5: 195–208. doi:10.1080/23288604.2019.1630595
10. Tangcharoensathien V, Pitayarangsarit S, Patcharanarumol W, Prakongsai P, Sumalee H, Tosanguan J, et al. Promoting universal financial protection: how the Thai universal coverage scheme was designed to ensure equity. Heal Res policy Syst. 2013;11: 25. doi:10.1186/1478-4505-11-25
11. Tangcharoensathien V, Thammatach-Aree J, Witthayapipopsakul W, Viriyathorn S, Kulthanmanusorn A, Patcharanarumol W. Political economy of Thailand’s tax-financed universal coverage scheme. Bull World Health Organ. 2020;98: 140–145. doi:10.2471/BLT.19.239343
12. Tangcharoensathien V, Witthayapipopsakul W, Panichkriangkrai W, Patcharanarumol W, Mills A. Health systems development in Thailand: a solid platform for successful implementation of universal health coverage. Lancet (London, England). 2018;391: 1205–1223. doi:10.1016/S0140-6736(18)30198-3
13. Weraphong J, Pannarunothai S, Luxananun T, Junsri N, Deesawatsripetch S. Catastrophic health expenditure in an urban city: seven years after universal coverage policy in Thailand. Southeast Asian J Trop Med Public Health. 2013;44: 124–136.
14. Yiengprugsawan V, Carmichael G, Lim L-Y, Seubsman S, Sleigh A. Explanation of inequality in utilization of ambulatory care before and after universal health insurance in Thailand. Health Policy Plan. 2011;26: 105–114. doi:10.1093/heapol/czq028

**Vietnam**

1. Dao A. What it means to say “I Don’t have any money to buy health insurance” in rural Vietnam: How anticipatory activities shape health insurance enrollment. Soc Sci Med. 2020;266: 113335. doi:10.1016/j.socscimed.2020.113335
2. Giang NH, Oanh TTM, Anh Tuan K, Hong Van P, Jayasuriya R. Is Health Insurance Associated with Health Service Utilization and Economic Burden of Non-Communicable Diseases on Households in Vietnam? Heal Syst reform. 2020;6: 1–15. doi:10.1080/23288604.2019.1619065
3. Ho HT, Santin O, Ta HQ, Nga Thuy Thi N, Do UT. Understanding family-based health insurance enrolment among informal sector workers in a rural district of Vietnam: Adverse selection and key determinants. Glob Public Health. 2020; 1–12. doi:10.1080/17441692.2020.1864434
4. Le QN, Blizzard L, Si L, Giang LT, Neil AL. The evolution of social health insurance in Vietnam and its role towards achieving universal health coverage. Heal Policy OPEN. 2020;1: 100011. doi:https://doi.org/10.1016/j.hpopen.2020.100011
5. Liu X, Tang S, Yu B, Phuong NK, Yan F, Thien DD, et al. Can rural health insurance improve equity in health care utilization? a comparison between China and Vietnam. Int J Equity Health. 2012;11: 10. doi:10.1186/1475-9276-11-10
6. Mao W, Tang Y, Tran T, Pender M, Khanh PN, Tang S. Advancing universal health coverage in China and Vietnam: lessons for other countries. BMC Public Health. 2020;20: 1791. doi:10.1186/s12889-020-09925-6
7. Thi Thuy Nga N, FitzGerald G, Dunne MP. Family-Based Social Health Insurance for Informal Workers in Vietnam: Willingness to Pay and Its Determinants. Asia-Pacific J public Heal. 2018;30: 512–520. doi:10.1177/1010539518799785
8. Thi Thuy Nga N, FitzGerald G, Dunne M. Family-Based Health Insurance for Informal Sector Workers in Vietnam: Why Does Enrolment Remain Low? Asia-Pacific J public Heal. 2018;30: 699–707. doi:10.1177/1010539518807601
9. Nguyen CV. The impact of voluntary health insurance on health care utilization and out-of-pocket payments: new evidence for Vietnam. Health Econ. 2012;21: 946–966. doi:10.1002/hec.1768
10. Nguyen HTH, Bales S, Wagstaff A, Dao H. Getting Incentives Right? The Impact of Hospital Capitation Payment in Vietnam. Health Econ. 2017;26: 263–272. doi:10.1002/hec.3294
11. Nguyen HT, Luu TV, Leppert G, De Allegri M. Community preferences for a social health insurance benefit package: an exploratory study among the uninsured in Vietnam. BMJ Glob Heal. 2017;2: e000277. doi:10.1136/bmjgh-2016-000277
12. Nguyen KT, Khuat OTH, Ma S, Pham DC, Khuat GTH, Ruger JP. Coping with health care expenses among poor households: evidence from a rural commune in Vietnam. Soc Sci Med. 2012;74: 724–733. doi:10.1016/j.socscimed.2011.10.027
13. Nguyen KT, Khuat OTH, Ma S, Pham DC, Khuat GTH, Ruger JP. Impact of health insurance on health care treatment and cost in Vietnam: a health capability approach to financial protection. Am J Public Health. 2012;102: 1450–1461. doi:10.2105/AJPH.2011.300618
14. Nguyen LH, Hoang ATD. Willingness to Pay for Social Health Insurance in Central Vietnam. Front public Heal. 2017;5: 89. doi:10.3389/fpubh.2017.00089
15. Nguyen TD, Wilson A. Coverage of health insurance among the near-poor in rural Vietnam and associated factors. Int J Public Health. 2017;62: 63–73. doi:10.1007/s00038-016-0911-z
16. Nguyen TH, Leung S. Dynamics of health insurance enrollment in Vietnam, 2004–2006. J Asia Pacific Econ. 2013;18: 594–614. doi:10.1080/13547860.2013.803842
17. Palmer M, Mitra S, Mont D, Groce N. The impact of health insurance for children under age 6 in Vietnam: A regression discontinuity approach. Soc Sci Med. 2015;145: 217–226. doi:10.1016/j.socscimed.2014.08.012
18. Phuong NK, Oanh TTM, Phuong HT, Tien T Van, Cashin C. Assessment of systems for paying health care providers in Vietnam: implications for equity, efficiency and expanding effective health coverage. Glob Public Health. 2015;10 Supppl: S80-94. doi:10.1080/17441692.2014.986154
19. Sepehri A, Sarma S, Oguzoglu U. Does the financial protection of health insurance vary across providers? Vietnam’s experience. Soc Sci Med. 2011;73: 559–567. doi:10.1016/j.socscimed.2011.06.009
20. Thuong NTT. Impact of health insurance on healthcare utilisation patterns in Vietnam: a survey-based analysis with propensity score matching method. BMJ Open. 2020;10: e040062. doi:10.1136/bmjopen-2020-040062
21. Thuong NTT, Huy TQ, Tai DA, Kien TN. Impact of Health Insurance on Health Care Utilisation and Out-of-Pocket Health Expenditure in Vietnam. Biomed Res Int. 2020;2020: 9065287. doi:10.1155/2020/9065287
22. Thu Thuong NT, Van Den Berg Y, Huy TQ, Tai DA, Anh BNH. Determinants of catastrophic health expenditure in Vietnam. Int J Health Plann Manage. 2021;36: 316–333. doi:10.1002/hpm.3076
23. Vuong DA, Flessa S, Marschall P, Ha ST, Luong KN, Busse R. Determining the impacts of hospital cost-sharing on the uninsured near-poor households in Vietnam. Int J Equity Health. 2014;13: 40. doi:10.1186/1475-9276-13-40
24. Wagstaff A, Nguyen HTH, Dao H, Bales S. Encouraging Health Insurance for the Informal Sector: A Cluster Randomized Experiment in Vietnam. Health Econ. 2016;25: 663–674. doi:10.1002/hec.3293

**Multiple countries**

1. Chu A, Kwon S, Cowley P. Health Financing Reforms for Moving towards Universal Health Coverage in the Western Pacific Region. Heal Syst reform. 2019;5: 32–47. doi:10.1080/23288604.2018.1544029
2. Tangcharoensathien V, Patcharanarumol W, Ir P, Aljunid SM, Mukti AG, Akkhavong K, et al. Health-financing reforms in southeast Asia: challenges in achieving universal coverage. Lancet (London, England). 2011;377: 863–873. doi:10.1016/S0140-6736(10)61890-9
3. Vilcu I, Probst L, Dorjsuren B, Mathauer I. Subsidized health insurance coverage of people in the informal sector and vulnerable population groups: trends in institutional design in Asia. Int J Equity Health. 2016;15: 165. doi:10.1186/s12939-016-0436-3

**B Grey literature publications**

**Cambodia**

1. Annear P, Grundy J, Ir P, Jacobs B, Men C, Nachtnebel M, et al. The Kingdom of Cambodia Health System Review. Health System in Transition. 2015.
2. Deutsche Gesellschaft für International Zusammenarbeit (GIZ) GmbH and World Health Organization (WHO). Toward strategic purchasing. Management of multiple schemes and purchasing decisions by the Cambodian National Social Security Fund. Bonn; 2020.
3. Kolesar RJ. Comparing Social Health Protection Schemes in Cambodia. Challenges and Opportunities Related to Coverage Expansion. Washington DC; 2019.
4. Levine D, Polimeni R, Ramage I. Insuring health or insuring wealth? An experimental evaluation of health insurance in rural Cambodia. Impact Analysis Series, No. 8. Paris; 2012.
5. Ministry of Health Cambodia. Health Equity Fund Operation Manual. Phnom Penh; 2017.
6. Ministry of Health Cambodia. Financial Manual For Health Equity Fund. Phnom Penh; 2017.
7. Ministry of Health Cambodia. Guidelines for the Benefit package and Provider Payment of the Health Equity Fund for the Poor. Phnom Penh; 2018.
8. Polimeni R, Levine D. Adverse selection based on observable and unobservable factors in health insurance. Impact Analysis Series, No. 10. Paris; 2012.

**Indonesia**

1. Australia Indonesia Partnership for Health Systems Strengthening (AIPHSS). Health Financing and Universal Health Coverage. Compilation of Policy Notes. Jakarta; 2015.
2. Banerjee A, Finkelstein A, Hanna R, Olken B, Ornaghi A, Sumarto S. Subsidies and the Dynamics of Selection: Experimental Evidence From Indonesia’s National Health Insurance. Cambridge; 2020.
3. Dartanto T. Universal Health Coverage in Indonesia: Informality, Fiscal Risks and Fiscal Space for Financing UHC. Tokyo; 2017.
4. Dartanto T, Rezki J, Usman, Siregar C, Bintara H, Pramono W. Expanding Universal Health Coverage in The Presence of Informality in Indonesia: Challenges and Policy Implications. 2015.
5. Deloitte Deloitte Asia Pacific Limited. Ensuring the Sustainability of JKN-KIS for the Indonesian People. Intended to cover the health costs of all Indonesians, the JKN-KIS national insurance program is now on the brink. How can this program survive? Jakarta; 2019.
6. Dwicaksono A, Nurman A, Prasetya P. JAMKESMAS and District Health Care Insurance Schemes. Assessment Reports from 8 Districts/Municipalities and 2 Provinces. Bandung; 2012.
7. Harimurti P, Pambudi E, Pigazzini A, Tandon A. The Nuts & Bolts of Jamkesmas Indonesia’s Government Financed Health Coverage Program. Washington D.C.; 2013.
8. International Labour Organization. Easing Access to the National Health Insurance through a Mobile Application. Geneva; 2019.
9. Kartika D. Does Indonesian National Health Insurance serve a potential for improving health equity in favour of workers in informal economy? London; 2015.
10. Ly C. Essays on Universal Health Coverage in Indonesia. University of Pennsylvania. Publicly Accessible Penn Dissertations. 2018.
11. Mahendradhata Y, Trisnantoro L, Listyadewi S, Soewondo P, Marthias T, Harimurti P, et al. The Republic of Indonesia Health System Review. Health Systems in Transition. Vol 7 No. 1. Geneva; 2017.
12. Marzoeki P, Tandon A, Bi X, Pambudi E. Universal Health Coverage for Inclusive and Sustainable Development: Country Summary Report for Indonesia. Washington D.C.; 2014.
13. President of the Republic of Indonesia. Regulation of President of the Republic of Indonesia. No 12/2013. 111 Indonesia; 2013.
14. President of the Republic of Indonesia. Regulation of the Minister of Health of the Republic of Indonesia. No 51/2018. Indonesia; 2019.
15. Putri A. Strategic purchasing. Governance of a purchasing market & the role of government. What can we learn from Indonesia? Jakarta; 2017.
16. Trisnantoro L, Hendrartini J, Susilowati T, Miranti PAD, Aristianti V. A critical analysis of selected healthcare purchasing mechanisms in Indonesia. Strateg Purch China, Indones Philipp. World Health Organization; 2016 Jun.
17. World Bank. Indonesia Health Financing System Assessment. Washington D.C.; 2016.

**Lao PDR**

1. Akkhavong, K Paphassarang, C Phoxay C, Vonglokham M, Phommavong, C Pholsena S. Lao People’s Democratic Republic Health System Review. Health System in Transition. 2014.
2. International Labour Organization. Moving towards universal social health protection. Lao People’s Democratic Republic (PDR). Geneva; 2019.
3. Lao People’s Democratic Republic. Decree On National Health Insurance Fund. Lao PDR: Vientiane; 2012.
4. Lao People’s Democratic Republic. National Social Protection Strategy. Vision 2030. Goal 2025. Vientiane; 2020. Phoummalaysith B, Senchanthixay M, Phonvisay B, Sengdara L, Manivong D, Yu S, et al. National Health Insurance in Lao PDR: Accelerating Progress towards UHC. Vientiane; 2020.
5. Phoummalaysith B, Senchanthixay M, Phonvisay B, Sengdara L, Manivong D, Yu S, et al. National Health Insurance in Lao PDR: Accelerating Progress towards UHC. Vientiane; 2020.
6. Sorensen B, Masaki E, Panyanouvong T, Vongsonephet T, Thitsy S, Chamleunsab M, et al. Managing transitions: Reaching the Vulnerable while Pursuing Universal Health Coverage (Vol 2). Health financing assessment in Lao PDR (English). Washington D.C.; 2017.
7. United Nations ESCAP. Roles of Social Health Protection in achieving UHC in Lao PDR. Inclusive Social Protection Systems in Asia and the Pacific: An expert group meeting. Bangkok; 2019.
8. World Health Organization Regional Office for the Western Pacific. Overview of Lao Health System Development 2009–2017. Manila; 2018.

**Malaysia**

1. Harvard T.H. Chan School of Public Health. Malaysia Health Systems Research Volume I. Contextual Analysis of the Malaysian Health System. Boston; 2016.
2. Jaafar S, Noh K, Muttalib K, Othman N, Healy J, Maskon K, et al. Malaysia Health System Review. Health System in Transition. 2012.
3. Ng C-W. Universal Health Coverage Assessment. Malaysia. 2015.
4. Quek DK. The Malaysian Health Care System: A Review. Kuala Lumpur; 2014.
5. Yap W, Razif I, Nagpal S. Universal Health Coverage Study Series No. 42. Malaysia: A new public clinic built every four days. Washington D.C.; 2019.

**Myanmar**

1. Sein T, Myint P, Tin N, Win H, Aye S, Sein T. The Republic of the Union of Myanmar Health System Review. Health System in Transition. 2014.
2. Teo H, Cain J. Myanmar Health Financing System Assessment. Discussion paper. Washington D.C.; 2018.
3. Tessier L, Thidar M. Evaluation of the operations of the Social Security Board, Ministry of Labour, Employment and Social Security of Myanmar. ILO-MDRI technical report. Geneva; 2014.
4. Tessier L, Guillebert J. Extending the network of health care facilities of the Social Security Board. Technical report on the feasibility of a Purchaser Provider Split. Geneva; 2015.
5. World Bank. Moving toward UHC: Myanmar - national initiatives, key challenges, and the role of collaborative activities (English). Washington D.C.; 2017.
6. World Health Organization. How can financial risk protection be expanded in Myanmar? Geneva; 2015.

**The Philippines**

1. Bredenkamp C, Buisman L. Universal Health Coverage in the Philippines: Progress on Financial Protection Goals. Policy Research Working Paper 7258. Washington D.C.; 2015. Report No.: Policy Research Working Paper 7258.
2. Dayrit M, Lagrada L, Picazo O, Pons M, Villaverde M. The Philippines Health System Review. Health System in Transition. Vol. 8 No. 2. New Delhi; 2018.
3. Kaiser K, Bredenkamp C, Iglesias R. Sin Tax Reform in the Philippines. Transforming Public Finance, Health, and Governance for More Inclusive Development. Washington D.C.; 2016.
4. Manasan R. Expanding Social Health Insurance Coverage: New Issues and Challenges, PIDS Discussion Paper Series, No. 2011-21. Makati City; 2011.
5. Philippine Health Insurance Corporation. Stats and Charts. 2020 (1st Semester). Pasig City; 2020.
6. Philippine Health Insurance Corporation. Universal Health Care Act. Frequently Asked Questions. Master Guide. Pasig City; 2020.
7. Philippine Institute for Development Studies. Who benefits from the government health insurance subsidy for the poor? Policy notes, No. 2017-05. 2017.
8. Phily C, Rajkotia Y, Matul M. Extending Universal Health Coverage for the Informal Sector in Philippines. Quezon City; 2014.
9. Republic of the Philippines. Congress of the Philippines. Republic Act No. 10606. Philippines; 2013.
10. Republic of the Philippines. Congress of the Philippines. Republic Act No. 11223. Universal Health Care Act. Philippines; 2019.
11. Sigua J, Ong M, Nuevo C, Boxshall M. The Philippine UHC Law Series: Brief 1. An introduction to the Philippine Universal Health Care Law. Washington DC; 2020.
12. Nuevo C, Sigua J, Boxshall M. The Philippine UHC Law Series: Brief 3. Health Financing in the Philippines. Washington DC; 2020.
13. Villaverde M, Gepte IV A, Baquiran R. Performance Assessment of the National Objectives for Health Philippines. 2011-2016. Quezon City; 2016.
14. World Bank. Philippine Health Sector Review. Transforming the Philippine Health Sector: Challenges and Future Directions. Washington D.C.; 2011.

**Thailand**

1. Ghislandi S, Manachotphong W, Perego V. The impact of Universal Health Coverage on healthcare consumption and risky behaviours: evidence from Thailand. Discussion paper 2013/3. London; 2013.
2. Gruber J, Hendren N, Townsend R. Demand and reimbursement effects of healthcare reform: Healthcare utilization and infant mortality in Thailand. Cambridge; Report No.: Working Paper 17739.
3. Hanvoravongchai P. Health Financing Reform in Thailand: Toward Universal Coverage under Fiscal Constraints. Washington D.C.; 2013.
4. Health Insurance System Research Office. Thailand’s Universal Coverage Scheme: Achievements and Challenges. An independent assessment of the first 10 years (2001-2010). Nonthaburi; 2012.
5. International Labour Organization. Universal Health-care Coverage Scheme. Thailand. Geneva; 2016.
6. Jongudomsuk P, Srithamrongsawat S, Patcharanarumol W, Limwattananon S, Pannarunothai S, Vapatanavong P, et al. The Kingdom of Thailand health system review. 2015.
7. Limwattananon S, Vongmongkol V, Prakongsai P, Patcharanarumol W, Hanson K, Tangcharoensathien V, et al. The equity impact of Universal Coverage: health care finance, catastrophic health expenditure, utilization and government subsidies in Thailand. Bangkok; 2011.
8. National Health Security Office. The management of provider payments in the universal coverage scheme (UCS) in Thailand. Bangkok; 2020.
9. National Health Security Office. Thailand UHC & overview of the universal coverage scheme of the National Health Security Office. Bangkok; 2020.
10. World Health Organization. UHC Law in Practice. Legal access rights to health care country profile: Thailand. Geneva; 2019.
11. Wagstaff A, Manachotphong W. Universal Health Care and Informal Labor Markets. The Case of Thailand. Policy Research Working Paper 6116. Washington D.C.; 2012. Report No.: Policy Research Working Paper 6116.

**Vietnam**

1. Barroy H, Jarawan E, Bales S. Universal Health Coverage for Inclusive and Sustainable Development : Country Summary Report for Vietnam. Washington D.C.; 2014.
2. Barroy H, Jarawan E, Bales S. Vietnam: Learning from Smart Reforms on the Road to Universal Health Coverage. Discussion Paper. Geneva; 2014.
3. Castel P, Tran O, Tran N, Tam T, Dat V. Health Insurance in Viet Nam towards Universal Coverage: The Case of the Workers of the Informal Sector. Policy Research Stury. Hanoi; 2011.
4. Huong N, Tuan L, Meissner M, Tuan B, Quyen D, Yen N. Social Protection for the Informal Sector and the Informally Employed in Vietnam. Literature and Data Review. Bochum; 2013. Report No.: IEE Working Paper, Volume 199.
5. International Labour Organization. Expanding Social Health Protection: Towards Equitable Coverage in Viet Nam. Geneva; 2019.
6. Ministry of Health Vietnam. Joint Annual Health Review. Strengthening grassroots health care towards universal health coverage. Hanoi; 2015.
7. Oan T, Phuong H. Strategic purchasing for universal health coverage: A critical assessment. Social Insurance Fund, Vietnam. Hanoi; 2016.
8. Somanathan A, Tandon A, Dao H, Hurt K, Fuenzalida-Puelma H. Moving toward Universal Coverage of Social Health Insurance in Vietnam. Assessment and Options. Washington D.C.; 2014.
9. Teo H, Bales S, Bredenkamp C, Cain J. The future of health financing in Vietnam: Ensuring sufficiency, efficiency, and sustainability. Washington D.C.; 2019.
10. Tien T, Phuong H, Mathauer I, Phuong N. A health financing review of Vietnam with a focus on social health insurance. Geneva; 2011.
11. UN Viet Nam. One UN results report 2019. Hanoi; 2019.
12. World Bank. Moving toward UHC: Vietnam - national initiatives, key challenges, and the role of collaborative activities (English). Washington D.C.; 2017.

**Multiple countries**

1. Acharya A, Vellakkal S, Taylor F, Masset E, Satija A, Burke M, et al. Impact of national health insurance for the poor and non-poor informal workers in low- and middle-income countries: a systematic review. London; 2012.
2. Annear P, Comrie-Thomson L, Dayal P. The challenge of extending universal coverage to non-poor informal workers in low- and middle-income countries in Asia. Impacts and policy options. Geneva; 2015.
3. Bitran R. Universal health coverage and the challenge of informal employment: lessons from developing countries. Washington DC; 2014.
4. Cotlear D, Nagpal S, Smith O, Tandon A, Cortez R. Going Universal. How 24 developing countries are implementing universal health coverage reforms from the bottom up. Washington DC; 2015.
5. International Labour Organization. Integrating Social Health Protection Systems Lessons learned. Geneva; 2019.
6. Bonfert A, Özaltin A, Heymann M, Hussein K, Hennig, J Langenbrunner J. Closing the gap: Health coverage for non-poor informal-sector workers. 2015.
7. Nakhimovsky S, Abiodun O, Koon A, Cico A. Exanding Coverage to Informal Workers: A Study of EPCMD Countries’ Efforts to Date. Washington D.C.; 2017.
8. Nguyen Q, Simoes da Cunha N. Extension of social security to workers in informal employment in the ASEAN region. Geneva; 2019.

**List 2. Excluded peer-reviewed articles and grey literature publications (full-text screening stage).**

1. Ministry of Health Cambodia. Guidelines for the Benefit package and Provider Payment of the Health Equity Fund for the Poor. Phnom Penh; 2018.

2. Ministry of Health Cambodia. Health Equity Fund Operation Manual. Phnom Penh; 2017.

3. Deutsche Gesellschaft für International Zusammenarbeit (GIZ) GmbH and World Health Organization (WHO). Toward strategic purchasing. Management of multiple schemes and purchasing decisions by the Cambodian National Social Security Fund. Bonn; 2020.

4. Annear P, Grundy J, Ir P, Jacobs B, Men C, Nachtnebel M, et al. The Kingdom of Cambodia Health System Review. Health System in Transition. 2015.

5. President of the Republic of Indonesia. Regulation of President of the Republic of Indonesia. No 12/2013. 111 Indonesia; 2013.

6. Mahendradhata Y, Trisnantoro L, Listyadewi S, Soewondo P, Marthias T, Harimurti P, et al. The Republic of Indonesia Health System Review. Health Systems in Transition. Vol 7 No. 1. Geneva; 2017.

7. President of the Republic of Indonesia. Regulation of the Minister of Health of the Republic of Indonesia. No 51/2018. Indonesia; 2019.

8. Deloitte Deloitte Asia Pacific Limited. Ensuring the Sustainability of JKN-KIS for the Indonesian People. Intended to cover the health costs of all Indonesians, the JKN-KIS national insurance program is now on the brink. How can this program survive? Jakarta; 2019.

9. Trisnantoro L, Hendrartini J, Susilowati T, Miranti PAD, Aristianti V. A critical analysis of selected healthcare purchasing mechanisms in Indonesia. Strateg Purch China, Indones Philipp. World Health Organization; 2016 Jun.

10. Agustina R, Dartanto T, Sitompul R, Susiloretni KA, Suparmi, Achadi EL, et al. Universal health coverage in Indonesia: concept, progress, and challenges. Lancet (London, England). 2019;393: 75–102. doi:10.1016/S0140-6736(18)31647-7

11. International Labour Organization. Moving towards universal social health protection. Lao People’s Democratic Republic (PDR). Geneva; 2019.

12. Chaleunvong K, Phoummalaysith B, Phonvixay B, Vonglokham M, Sychareun V, Durham J, et al. Factors associated with patient payments exceeding National Health Insurance fees and out-of-pocket payments in Lao PDR. Glob Health Action. 2020;13: 1791411. doi:10.1080/16549716.2020.1791411

13. United Nations ESCAP. Roles of Social Health Protection in achieving UHC in Lao PDR. Inclusive Social Protection Systems in Asia and the Pacific: An expert group meeting. Bangkok; 2019.

14. Bodhisane S, Pongpanich S. The impact of National Health Insurance upon accessibility of health services and financial protection from catastrophic health expenditure: a case study of Savannakhet province, the Lao People’s Democratic Republic. Heal Res policy Syst. 2019;17: 99. doi:10.1186/s12961-019-0493-3

15. Sorensen B, Masaki E, Panyanouvong T, Vongsonephet T, Thitsy S, Chamleunsab M, et al. Managing transitions: Reaching the Vulnerable while Pursuing Universal Health Coverage (Vol 2). Health financing assessment in Lao PDR (English). Washington D.C.; 2017.

16. Phoummalaysith B, Senchanthixay M, Phonvisay B, Sengdara L, Manivong D, Yu S, et al. National Health Insurance in Lao PDR: Accelerating Progress towards UHC. Vientiane; 2020.

17. Jaafar S, Noh K, Muttalib K, Othman N, Healy J, Maskon K, et al. Malaysia Health System Review. Health System in Transition. 2012.

18. Harvard T.H. Chan School of Public Health. Malaysia Health Systems Research Volume I. Contextual Analysis of the Malaysian Health System. Boston; 2016.

19. Yap W, Razif I, Nagpal S. Universal Health Coverage Study Series No. 42. Malaysia: A new public clinic built every four days. Washington D.C.; 2019.

20. Quek DK. The Malaysian Health Care System: A Review. Kuala Lumpur; 2014.

21. Tessier L, Guillebert J. Extending the network of health care facilities of the Social Security Board. Technical report on the feasibility of a Purchaser Provider Split. Geneva; 2015.

22. Tessier L, Thidar M. Evaluation of the operations of the Social Security Board, Ministry of Labour, Employment and Social Security of Myanmar. ILO-MDRI technical report. Geneva; 2014.

23. De L, Anh Q, Tsuruga I, Ruck M. An assessment of the social protection needs and gaps for workers in informal employment in Myanmar. Geneva; 2019.

24. Sein T, Myint P, Tin N, Win H, Aye S, Sein T. The Republic of the Union of Myanmar Health System Review. Health System in Transition. 2014.

25. Obermann K, Jowett M, Kwon S. The role of national health insurance for achieving UHC in the Philippines: a mixed methods analysis. Glob Health Action. 2018;11: 1483638. doi:10.1080/16549716.2018.1483638

26. Republic of the Philippines. Congress of the Philippines. Republic Act No. 11223. Universal Health Care Act. Philippines; 2019.

27. Republic of the Philippines. Congress of the Philippines. Republic Act No. 10606. Philippines; 2013.

28. Bredenkamp C, Buisman L. Universal Health Coverage in the Philippines: Progress on Financial Protection Goals. Policy Research Working Paper 7258. Washington D.C.; 2015. Report No.: Policy Research Working Paper 7258.

29. Dayrit M, Lagrada L, Picazo O, Pons M, Villaverde M. The Philippines Health System Review. Health System in Transition. Vol. 8 No. 2. New Delhi; 2018.

30. Nuevo C, Sigua J, Boxshall M. The Philippine UHC Law Series: Brief 3. Health Financing in the Philippines. Washington DC; 2020.

31. National Health Security Office. Thailand UHC & overview of the universal coverage scheme of the National Health Security Office. Bangkok; 2020.

32. Jongudomsuk P, Srithamrongsawat S, Patcharanarumol W, Limwattananon S, Pannarunothai S, Vapatanavong P, et al. The Kingdom of Thailand health system review. 2015.

33. National Health Security Office. The management of provider payments in the universal coverage scheme (UCS) in Thailand. Bangkok; 2020.

34. Tangcharoensathien V, Witthayapipopsakul W, Panichkriangkrai W, Patcharanarumol W, Mills A. Health systems development in Thailand: a solid platform for successful implementation of universal health coverage. Lancet (London, England). 2018;391: 1205–1223. doi:10.1016/S0140-6736(18)30198-3

35. Oan T, Phuong H. Strategic purchasing for universal health coverage: A critical assessment. Social Insurance Fund, Vietnam. Hanoi; 2016.

36. Le QN, Blizzard L, Si L, Giang LT, Neil AL. The evolution of social health insurance in Vietnam and its role towards achieving universal health coverage. Heal Policy OPEN. 2020;1: 100011. doi:https://doi.org/10.1016/j.hpopen.2020.100011

37. Teo H, Bales S, Bredenkamp C, Cain J. The future of health financing in Vietnam: Ensuring sufficiency, efficiency, and sustainability. Washington D.C.; 2019.

38. World Bank. Moving toward UHC: Vietnam - national initiatives, key challenges, and the role of collaborative activities (English). Washington D.C.; 2017.

39. Ministry of Health Vietnam. Joint Annual Health Review. Strengthening grassroots health care towards universal health coverage. Hanoi; 2015.

40. Barroy H, Jarawan E, Bales S. Universal Health Coverage for Inclusive and Sustainable Development : Country Summary Report for Vietnam. Washington D.C.; 2014.

41. Phuong NK, Oanh TTM, Phuong HT, Tien T Van, Cashin C. Assessment of systems for paying health care providers in Vietnam: implications for equity, efficiency and expanding effective health coverage. Glob Public Health. 2015;10 Supppl: S80-94. doi:10.1080/17441692.2014.986154

42. Levine D, Polimeni R, Ramage I. Insuring health or insuring wealth? An experimental evaluation of health insurance in rural Cambodia. Impact Analysis Series, No. 8. Paris; 2012.

43. Dartanto T. Universal Health Coverage in Indonesia: Informality, Fiscal Risks and Fiscal Space for Financing UHC. Tokyo; 2017.

44. Ly C. Essays on Universal Health Coverage in Indonesia. University of Pennsylvania. Publicly Accessible Penn Dissertations. 2018.

45. Dartanto T, Halimatussadiah A, Rezki JF, Nurhasana R, Siregar CH, Bintara H, et al. Why Do Informal Sector Workers Not Pay the Premium Regularly? Evidence from the National Health Insurance System in Indonesia. Appl Health Econ Health Policy. 2020;18: 81–96. doi:10.1007/s40258-019-00518-y

46. International Labour Organization. Integrating Social Health Protection Systems Lessons learned. Geneva; 2019.

47. World Health Organization Regional Office for the Western Pacific. Overview of Lao Health System Development 2009–2017. Manila; 2018.

48. Kaiser K, Bredenkamp C, Iglesias R. Sin Tax Reform in the Philippines. Transforming Public Finance, Health, and Governance for More Inclusive Development. Washington D.C.; 2016.

49. Limwattananon S, Neelsen S, O’Donnell O, Prakongsai P, Tangcharoensathien V, van Doorslaer E, et al. Universal coverage with supply-side reform: The impact on medical expenditure risk and utilization in Thailand. J Public Econ. 2015;121.

50. International Labour Organization. Expanding Social Health Protection: Towards Equitable Coverage in Viet Nam. Geneva; 2019.

51. Palmer M, Mitra S, Mont D, Groce N. The impact of health insurance for children under age 6 in Vietnam: A regression discontinuity approach. Soc Sci Med. 2015;145: 217–226. doi:10.1016/j.socscimed.2014.08.012

52. Thuong NTT. Impact of health insurance on healthcare utilisation patterns in Vietnam: a survey-based analysis with propensity score matching method. BMJ Open. 2020;10: e040062. doi:10.1136/bmjopen-2020-040062

53. Thuong NTT, Huy TQ, Tai DA, Kien TN. Impact of Health Insurance on Health Care Utilisation and Out-of-Pocket Health Expenditure in Vietnam. Biomed Res Int. 2020;2020: 9065287. doi:10.1155/2020/9065287

54. Liu X, Tang S, Yu B, Phuong NK, Yan F, Thien DD, et al. Can rural health insurance improve equity in health care utilization? a comparison between China and Vietnam. Int J Equity Health. 2012;11: 10. doi:10.1186/1475-9276-11-10

55. Somanathan A, Tandon A, Dao H, Hurt K, Fuenzalida-Puelma H. Moving toward Universal Coverage of Social Health Insurance in Vietnam. Assessment and Options. Washington D.C.; 2014.

56. Barroy H, Jarawan E, Bales S. Vietnam: Learning from Smart Reforms on the Road to Universal Health Coverage. Discussion Paper. Geneva; 2014.

**Table 7. Key findings and methodological details of the included peer-reviewed articles (numbers correspond to reference list in S1 List).**

| **No., Year** | **Study objectives** | **Country and target population** | **Health financing scheme** | **Study design, sampling technique, and evaluation design** | **Data source(s) and study period(s)** | **Key findings extracted from the included studies** | **Study quality score** |
| --- | --- | --- | --- | --- | --- | --- | --- |
| **Cambodia** | | | | | | | |
| (1), 2013 | To investigate the main policy and operational barriers to, and opportunities for, creating a national social health protection agency for the poor and the informal sector | Cambodia, poor and IWs without social health protection coverage | Governance structures for a social health protection scheme for IWs | Qualitative interview study and policy review  Purposive sampling  Organizational Assessment for Improving and Strengthening Health Financing (OASIS) as conceptual framework for data analysis | Primary data collection through semi-structured key informant interviews, and review of policy documents  September 2011 | The study found a shortfall in institutional, organizational, and health financing capacity, resulting in fragmentation and constraints in the implementation of SHP schemes. Increasing funding and building capacity for implementation of SHP schemes are two major challenges. Lessons from Cambodia’s initial steps taken towards UHC indicate that several critical factors emerge: the need to expand the fiscal space for health; a commitment to equity; measures to protect the poor; building national capacity for SHP and UHC administration; and working within the specific national context. | High |
| (2)**,** 2016 | To assess the impact of household size on decisions to enroll in CBHI and demonstrate how to correct for group disparity in scale (i.e. variance differences) | Cambodia, households eligible for CBHI | CBHI for IWs and their households | Discrete choice experiment  Cluster random sampling  Random utility model, conditional logit regression | Primary data collection using a discrete choice experiment survey  2010 | Significant utility increases were observed for higher coverage of hospital fees, increased coverage of meals and travel costs, as well as more frequent communication with the insurer. Increasing insurance premiums was associated with disutility. Before adjusting for scale, the magnitude of preference for hospital fee coverage appeared larger for the large household group compared to the small household group. After adjustment for the observed differences in scale between large and small household groups, preference differences by household size became negligible. | High |
| **Indonesia** | | | | | | | |
| (3), 2019 | To explore the achievements, gaps, and opportunities for NHIS to expand population coverage and equity, ensure quality of care, and enhance its impact on population health. | Indonesia, citizens of Indonesia and foreigners after at least six months | NHIS for the entire population, including IWs | Review  Narrative synthesis | Review of secondary peer-reviewed and grey literature. | The UHC system introduced in 2014 grew rapidly and is the largest single-payer scheme in the world. Covering 203 million people, it has improved health equity and service access. However, despite these successes, challenges have emerged, and equity gaps remain. Particularly the “missing-middle” problem has materialized and is characterized by people working in the informal sector not being covered under the NHIS due to low self-enrolment; the lower-middle-income group has the highest number of uninsured people. This makes it difficult to achieve UHC by 2019 as required by law. | Medium |
| (4), 2020 | To explore the important factors that affect the compliance behavior of informal sector workers in regularly paying their insurance premium, and to design effective measures for improving the sustainability of premium payments. | Indonesia, informal sector workers enrolled in the NHIS | NHIS for the entire population, including IWs | Descriptive cross-sectional study  Multi-stage sampling including stratified random sampling (last sampling stage)  Logistic regression (logit model) | Survey data collected from three regional offices of the Indonesian Social security Agency for Health  June-September 2015 | About 28% of IW members did not pay their premiums regularly. IWs are more prone to vulnerable conditions due to income uncertainty (e.g. fluctuations due to crop loss, price volatility, and climate factors, even if medium to high incomes on average). The following factors were associated with sustainability of insurance premium payments: age of household head, working sector (agricultural sector more likely sustainable), household income and income stability, savings, comprehensive knowledge of the scheme, availability of professional healthcare services, and having incurred inpatient costs before joining. Factors negatively associated with premium payment sustainability are increased numbers of family members, financial hardship, minimum knowledge of the scheme, member of other social protection schemes, increased distance to a hospital and no utilization of health services. | High |
| (5), 2016 | To measure the WTP for the health insurance program of workers in the informal sector and to shed light on why IWs are reluctant to join the NHIS. | Indonesia, informal sector workers enrolled in the NHIS | NHIS for the entire population, including IWs | Cross-sectional study  Purposive sampling of districts, random sampling of sub-districts and villages, purposive sampling of households  Triple Bounded Dichotomies Choice Contingent Valuation, logistic regression (logit model) | Primary data collection using a household survey  Time frame not stated | The average premium individuals were willing to pay for class 1 was Rp61,740 and at Rp40,685 (90% of the class 2’s premium at the point of the study) for class 2; the second class was the least preferable class. Class 3 was the most favorable class with an average premium of around Rp22,386, indicating that IWs prefer to pay a minimum premium, even though the other classes might be affordable for them. Overall, the survey observed that most of the households working in the informal sector experience some difficulty regarding the premium payment; there was a 10% gap between the lowest premium of JKN that they were willing to pay and the premium that they were required to pay. 30% of respondents were unwilling to join the NHIS at any of the offered premium rates.  Availability of hospitals at the district level was positively correlated with the likelihood of respondents’ willingness to join JKN. Similarly, experience as an inpatient one year before the survey, having insurance in the past, access to internet, and knowledge of health insurance were significantly positively associated with willingness-to-join the program. Presence of a doctor in the community had a significant and negative correlation with the WTP to join JKN (local doctor competing, and cheaper option plus potentially accepts non-monetary payments, making them more accessible). Larger households, male sex ad lower income levels were negatively correlated with willingness-to-join JKN. Main reasons for non-registration were 1) not enough money, 2) no knowledge about JNK, and 3) not knowing where to register. | Medium |
| (6), 2020 | To analyze aspects that influence individual or household decisions to enroll in the NHIS. | Indonesia, IWs eligible for and enrolled in the NHIS | NHIS for the entire population, including IWs | Qualitative interview study  Purposive sampling and snowball sampling  Thematic content analysis using inductive approaches | Primary data collection through qualitative interviews and focus group discussions  2014 and October-November 2017 | Most informants became BPJS-K members to cover their health needs on a rolling basis with the cheapest premiums available. Most informants also realized their future health needs and the health and economic risks they might face, thus deciding to cover themselves against it; they perceived health insurance as a safety net. BPJS-K was generally perceived as the cheapest option for coverage against health risks after a waiting period of only two weeks. Having previous (positive) experience with health insurance was also mentioned as a reason for signing up with NHIS. Family and social community were mentioned to be important influential decision factors. Informants also raised the importance of collaboration between BPJS-K officials and sub-district officers in providing official information about the NHIS to maximize the word-of-mouth effect of local/influential leaders and to avoid misinformation. | High |
| (7), 2019 | To examine whether the implementation of Indonesia’s JKN program improved access to health care, measured in terms of utilization, for its enrollees. | Indonesia, IWs voluntarily enrolled and individuals who are subsidized by government | NHIS for the entire population, including IWs | Impact evaluation  Logit model, propensity score matching combined with difference-in-difference approaches | Analysis of secondary data from the Indonesia Family Life Survey 2007 and 2014  2007 and 2014/15 | Compared to the uninsured, both the contributory and the subsidized group had higher proportion and frequency of utilization of outpatient and inpatient care in both years. Individuals covered by the JKN contributory scheme were younger, more likely to live in an urban area, wealthier, more likely to have completed higher education, and more likely to live in an area with more health facilities compared to the uninsured; subsidized individuals were poorer, less likely to finish higher education, more likely to receive cash transfers, and living in an area with fewer health facilities, confirming the assumption that both groups have fundamentally different characteristics. The JKN program increased the probability of seeking outpatient care (8.2% / --) and inpatient care (7.9% / 1.75%) in the contributory and the subsidized group, respectively, highlighting that inequities in access to both outpatient and inpatient care may remain. Almost no significant effect was observed in the area with low density of health facilities, while the effect was significant in high density areas for both groups, suggesting that the effect of health insurance can only be realized given the availability of nearby facilities. | High |
| (8), 2020 | To determine the factors associated with ownership of independent NHI in market traders, Gowa District, Indonesia. | Indonesia, IWs eligible for NHIS in Gowa district | NHIS for the entire population, including IWs | Cross-sectional survey  Simple random sampling  Univariate and bivariate statistical analysis (Chi-square test) | Primary data collection using household surveys  Time period not stated | In total, 50.6% of the informants had NHIS. Education, perception, age, and access to services were not significantly associated with NHIS ownership of IWs, while knowledge, income, attitude, and social support were highly statistically significant. | Low |
| (9), 2017 | To investigate the effect of these local health care financing schemes on access to health care and financial protection. | Indonesia, IWs enrolled in district health financing schemes | Prepayment scheme – Health insurance for the poor and IWs | Cross-sectional study design  No information on sampling available  Fixed effects regression model | Secondary data analysis using data from a national socioeconomic survey  2004-2010 | Almost all Jamkesda schemes covered services provided by local health centers (87%) and public district hospitals (82%). Referral to province level hospitals and national hospitals is covered by 69 and 14% of Jamkesda schemes, and many schemes also provided coverage for referrals to specific providers in other districts or provinces (76%); 23% had contracted private hospitals. There were variations in the benefit packages. Most of the impact on increased outpatient care utilization was concentrated with the 3^rd^ and 4^th^ quintiles (IWs and largely ineligible for Jamkesmas as these are main target for Jamkesda program). | High |
| (10), 2013 | To investigate targeting and impact of the Askeskin program. | Indonesia, IWs enrolled in the Askeskin program | Prepayment scheme – Health insurance for the poor and IWs | Cross-sectional study design  No information on sampling available  Difference-in-difference estimation in combination with propensity score matching | Secondary data analysis using data from a national socioeconomic survey  2005 and 2006 | Utilization for non-poor IWs is higher than for poor IWs. OOPE are higher in richer quartiles than in poorer quartile, reflecting differences in affordability of care and propensity to spend between poor and rich. The study found leakage of Askeskin coverage to the non-poor. Overall, Askeskin increased monthly outpatient utilization. Askeskin also seems to increase OOPE and budget shares, particularly in urban areas, suggesting that Askeskin members had to bear part of the costs of increased utilization. | High |
| **Lao PDR** | | | | | | | |
| (11), 2013 | To explore the determinants of enrolment at the household level, and to systematically assess how districts with and without CBHI differ in terms of factors that are expected to affect the demand for health care and insurance. | Lao PDR, informal sector households enrolled in the CBHI scheme and comparison households | Prepayment scheme – CBHI | Mixed methods study design (cross-sectional case-comparison study (household survey) and qualitative data collection (focus group discussions)  Two-stage cluster random sampling  Multivariate analysis using a probit model | Primary data collection using a household survey and focus group discussions  February to April 2009 | CBHI households are larger, more likely to be married, more educated than uninsured households, have higher consumption levels, have poorer self-rated health, higher prevalence of disability or chronic illness, increased difficulty performing activities, a higher proportion of households in which a household member has deteriorating health, more elderly household members, more women of reproductive age, more pregnant women, are relatively less risk-averse than uninsured households, report a higher perception of quality of healthcare at the district hospital, are more likely to have attended a CBHI campaign, to have close relatives/friends in the scheme, and to place higher trust in the scheme. | High |
| (12), 2013 | To estimate the CBHI's impact on utilization and OOPE. | Lao PDR, informal sector households enrolled in the CBHI scheme and comparison households | Prepayment scheme – CBHI | Mixed methods study design (cross-sectional case-comparison study (household survey) and qualitative data collection (key informant interviews (*ex ante*), focus group discussions (both *ex ante* and *ex post*))  Two-stage cluster random sampling  Propensity score matching with kernel matching and using bias-corrected matching | Primary data collection using a household survey, key informant interviews and focus group discussions  February to April 2009 | Significant increased utilization of both inpatient and outpatient services; CBHI members almost twice as likely to have an inpatient stay (1-year period). Insurance further found to encourage use of the referral system, and to increase use of public facilities while decreasing the likelihood that members will use private clinics.  Significantly lower OOPE for CBHI members compared to uninsured, though protective effect was limited to those who used services. | High |
| (13), 2019 | To assess the impact of NHI in providing accessibility to public hospitals and in offering financial protection from catastrophic expenditure related to health service utilization, when compared to the proceeding CBHI scheme | Lao PDR, informal sector households enrolled in NHI | Prepayment scheme – NHI | Cross-sectional study design  Purposive selection of two hospitals (1 district, 1 referral); systematic random sampling to select respondents  Binary logistic regression models | Primary data collection through a cross-sectional survey  September to November 2018 | Only existence of a chronic condition in the household significant predictor of hospital admission.  Married respondents, large households, and households’ level of income significantly increased the probability of accessibility to health service utilization under the NHI.  Households with an existing chronic condition had significantly higher chances of suffering CHE compared to households with healthy members. Larger-sized households (>5 members) bear larger portion of CHE. | Medium |
| (14), 2017 | To determine the role of CBHI in making health care services accessible and in preventing financial catastrophe resulting from personal payment for inpatient services. | Lao PDR, informal sector households enrolled in the CBHI scheme and comparison households | Prepayment scheme – CBHI | Cross-sectional study design  Simple random sampling  Logistic regression and chi-square analysis | Primary data collection using a household survey  July to September 2013 | No independent variable statistically significant at the 95% level to determine probability of hospitalization; respondents with a chronic condition considerably more likely to be hospitalized; smaller households marginally more likely to be hospitalized. Insurance status not found to increase the probability of utilization; no difference in health service accessibility between insured and uninsured households.  Educational level and income level found to be significant predictors for CHE (40% level), with lower-level households facing higher risks of CHE; chronic conditions also shown to have a high impact on CHE (not significant). CBHI not found to significantly lower the chance of experiencing CHE. Non-medical expenditure found to increase the chances of suffering CHE (not significant). | Medium |
| (15), 2018 | To find out the factors that significantly affect the CBHI enrollment incentive. | Lao PDR, IWs who are not enrolled in the CBHI scheme | Prepayment scheme – CBHI | Cross-sectional study design  Purposive selection of two districts; systematic random sampling to identify respondents  Logistic regression model | Primary data collection using a household survey  July to August 2017 | At the 95% confidence interval level, occupation (farmer and laborer less likely to enroll), awareness of CBHI scheme (individuals with no information/knowledge about CBHI more likely to enroll), district of residence (residents of Kaysone Phomvihane district more likely to enroll), and health service utilization (individuals using OPD within 3 months and/or IPD within 1 year more likely to enroll). Head of household's gender/marital status/age, size of the household, household's total monthly income no significant effect on enrolment decision. | Medium |
| (16), 2021 | To analyze and compare the probability of using health care services and the financial catastrophe occurring due to health service utilization in both local and Thai hospitals. | Lao PDR / Thailand, NHI beneficiaries seeking health services in Lao PDR and in Thailand | Prepayment scheme - NHI | Cross-sectional study design  Systematic random sampling  Binary logistic regression | Primary data collection using a household survey  Time period not stated | The presence of elderly and chronic conditions in households were associated with a higher probability of health service utilization in both Lao PDR and Thailand. Lower income quintiles had significantly higher levels of hospital admission than high-income quintiles in Lao PDR’s hospitals. On the contrary, higher-income households had an increased probability of using hospitals in Thailand when compared to the first two income quintiles. Lower-income households had higher probabilities of experiencing CHE (not significant) in Lao PDR. Respondents working in the private sector had significantly higher probabilities of experiencing CHE than street vendors. In terms of Thailand’s health service utilization, the two lowest quintiles and individuals of lower education levels had significantly higher levels of experiencing CHE. | Medium |
| (17), 2020 | To assess the knowledge of those enrolled in the NHI scheme in Lao PDR. | Lao PDR, IWs enrolled in the NHI scheme | Prepayment scheme – NHI | Cross-sectional descriptive study  Systematic random sampling  Poisson regression to determine factors associated with knowledge of NHI | Primary cross-sectional data collection  Study period not stated | Overall low understanding of the benefits of the NHI, the limits of coverage and co-payment policies. More innovative demand-side strategies are needed to create awareness of the NHI and its benefits. | Medium |
| (18),  2020 | To assess the percentage of NHI patients who paid above the scheduled amount, based on individual billing payment; and the factors related to overpayment. | Lao PDR, IWs enrolled in the NHI scheme | Prepayment scheme – NHI | Cross-sectional descriptive study  Multi-stage sampling stratified by facility level, cluster sampling to select facilities, and systematic random sampling for selecting outpatients and inpatients  Multiple logistic regression | Primary cross-sectional data collection  Study period not stated | About 20% paid above the defined amount of payment for outpatient and inpatient services. Among others, older age, higher-level facility usage, >5km distance from facility, buying medicines/supplies from outside of the health facility, not bringing documents or not having the right documents were associated with costs above the scheduled fees. | Low |
| (19), 2019 | To provide empirical evidence that the benefit package components of hypothetical CBHI schemes have causal effects on enrollment probabilities, and to examine the distribution of WTP in response to policy changes. | Lao PDR, IWs eligible for but not enrolled in CBHI | Prepayment scheme – CBHI | Randomized conjoint experiment (cross-sectional)  Purposive selection of province, districts, and villages; randomized selection of households  Conjoint analysis applying a full randomization design | Primary cross-sectional data collection  September 2016 | Premium found to be the largest influential factor on respondents’ willingness to join the CBHI; 82% of respondents reported inability to pay as constraint. The composition of the benefit package was further found to have a crucial impact on respondents’ probability of enrolling in the CBHI scheme; respondents were found to value a hypothetical policy alternative over the status quo. Respondents preferred CBHI schemes that include coverage for either traffic accidents or round-trip transportation; inclusion of the latter significantly increased enrolment probabilities and WTP. Authors conclude that low enrolment in CBHI scheme in Lao PDR does not necessarily indicate low demand in general but can be increased through benefits package improvements. | Medium |
| **Malaysia** | | | | | | | |
| (20), 2021 | To explore factors associated with not having private health insurance. | Malaysia, IWs who are not enrolled in private health insurance | Prepayment scheme – private health insurance | Cross-sectional study design  Sampling information not available  Descriptive statistics, binary logistic regression | Secondary data from the National Health and Morbidity Survey  2015 | Overall, 56.6% of the respondents was uninsured. Higher proportions of uninsured individuals were observed among unpaid workers (IWs), poorest 2 quintiles, self-employed, retiree, low education status, single/widowed/divorced, indigenous ethnicity, female, unemployed and individuals aged 50 years and above. | Medium |
| **Myanmar** | | | | | | | |
| (21),  2019 | To explore the knowledge, perceptions, and preferences of potential health insurance beneficiaries about the nature and size of health insurance premiums as well as cost‐sharing mechanisms and the health benefit package. | Myanmar, SSS members and general population (largely IWs) | Prepayment scheme - SSS | Cross-sectional study  Multistage cluster sampling  Mann-Whitney U test (ordinal variables) and independent sample t test (continuous variables); binary regression, linear logistic regression, sample selection regression | Primary cross-sectional data collection  June to August 2015 | Knowledge of general population of health insurance very low. Around 65% reported positive perceptions of health insurance on financial protection, perceived disease risk, trust, and on prepayment for healthcare; positive perceptions regarding a positive return on investment from health insurance and premium payment lower at about 35%. Around 75% of respondents were willing to pay health insurance contributions; healthier and younger individuals and individuals lacking trust in the system were significantly less willing to pay; contribution amounts increased with increases in trust in the scheme. About 40% were willing to pay increased contributions for enrollment of other household members. The majority preferred monthly premiums, while about 10% preferred quarterly payments. About 44% would further be willing to pay annual premiums for reduced prices. As a fund manager, respondents preferred a government body over private or community organizations. | Medium |
| (22),  2019 | To provide evidence on the patterns of health care use and OOPE by the general population and SSS beneficiaries in Myanmar. | Myanmar, SSS members and general population (largely IWs) | Prepayment scheme - SSS | Cross-sectional study  Multistage cluster sampling  Mann-Whitney U test (ordinal variables) and independent sample t test (continuous variables); two-step cluster analysis; binary logistic regression and multinomial logistic regression | Primary cross-sectional data collection  June to August 2015 | No significant difference in utilization between general population and SSS members. Individuals generally chose facilities in proximity and with the best perceived service quality. General population members reported significantly longer waiting times, significantly higher OOPE (eight times higher than in the SSS sample), and greatly increased amounts when borrowing to cover health expenditure (five times higher) and when selling assets. Respondents with higher incomes reported higher expenditures for their last use of health services. Individuals using specialized care or inpatient services reported an increased likelihood to borrow money or sell assets to cover the expenditures. Women were significantly more likely to use specialized or inpatient services than men. | High |
| **The Philippines** | | | | | | | |
| (23), 2016 | To see how far voluntary enrollment in the government’s SHI program responds to an intervention that combines information and an enrollment subsidy. | Philippines, IWs eligible for the voluntary component of the Philippines’ SHI program | Prepayment scheme – NHIP, voluntary component | Cluster randomized experiment  Multi-stage cluster sampling using systematic and random sampling; Random assignment of households to control group or treatment groups  OLS regression, probit model | Primary data collection through a baseline survey (February-April 2011) and an endline survey (March-May 2012) | The combined intervention of information and subsidy raised enrolment by approximately 37% (212 households); overall enrolment in the voluntary scheme was still only at 11%. City dwellers were more likely to enroll due to their closer proximity to the provincial PhilHealth office and their higher education status, rendering the enrolment process less mentally taxing for them. Adverse selection was not present; those reporting an adverse health event in the previous year reported a higher WTP for health insurance, though in the actual experiment, the difference in enrolment between those experiencing and not experiencing an adverse event is statistically insignificant. Combining home assistance with form-completion and the delivery of the completed form to the PhilHealth office plus re-mailing of the membership cards, produced an enrollment rate of 31% among those who previously had been provided with information about IPP and offered a 50% subsidy but had opted not enroll. | High |
| (24), 2018 | To analyze the role of the Philippine NHI scheme in moving towards UHC, identify potential avenues for improvement as well as indicate challenges and areas  for further development. | Philippines | Prepayment scheme – NHIP | Descriptive mixed methods study including a literature review, secondary data analysis, and key informant interviews  Purposive sampling of participants  Analysis techniques not stated | Primary data collection using key informant interviews as well as secondary data analysis  Time period not stated | The Philippines has made major achievements in its aim to reach UHC. The UHC bill passed in 2017 provides an opportunity to automatically enroll all Filipinos under the NHI and to entitle them to the benefits of PhilHealth and constitutes a paradigmatic change in thinking about NHI. The introduction of the ‘Sin Tax’ as an earmarked revenue source, the introduction of the no-balance-billing to prevent co-payments, and the Health Facilities Enhancement Program aimed to improve quality are among the successes. However, OOPE remain high and the share of PhilHealth in total health expenditures is only at 14%, quality management of providers requires improvement, and the health benefits package is not reflective of the country’s disease burden, thus limiting financial protection. Expanding NHI and reaching UHC requires a large increase in budget transfers to cover citizens who are currently unable to contribute. | Low |
| (25), 2018 | To quantitatively and qualitatively describe some of the challenges faced by the Philippines’ health insurance program, PhilHealth, in the era of Universal  Health Coverage. | Philippines, sponsored members who are primarily from the lower income segment of the informal economy | Prepayment scheme – NHIP | Descriptive mixed methods study including key informant interviews and analysis of quantitative secondary data sources  No details on sampling  Qualitative content analysis | Primary data collection using key informant interviews  February to October 2017 | In all local government units, the number of individuals enrolled as indigents and sponsored members was higher than those identified as eligible by the Department of Social Welfare and Development. Reported reasons for this are a lack of a standardized procedure to determine who is and who is not eligible for the categories and certificates are therefore occasionally inappropriately issued. Participants further stated that near-poor IWs prioritize food spending over enrolment in the SHI. Accreditation and reimbursement processes for healthcare providers were reportedly slow, changing without notice, or failed to materialize, leading facilities to decide not to renew accreditation. | Low |
| (26), 2013 | To quantify the extent to which beneficiaries of the NHIP incur out-of-pocket expenses for inpatient care and examine the characteristics of beneficiaries making these payments and the hospitals in which these payments are typically made. | Philippines, IWs enrolled in the individually paying program of the NHIP | Prepayment scheme – NHIP | Secondary data analysis  Probit model, ordinary least squares model | Secondary data extracted from the PhilHealth inpatient claims database between 2007 and 2009 | Only 14% of claims required no OOPE, while 13% of claims involved OOPE equivalent to 10% of the average annual family income in the region. The median level of the support value per claim was 57%, and the mean level of support value was 42% on average. The higher the level of the hospital, the larger the total charge, NHIP benefits and OOPE were, despite the NHIP’s attempt to mitigate this by setting different benefit ceilings based on the level of hospital; this reflects the fact that many patients in the Philippines bypass lower levels of care, while tertiary hospitals continue to admit cases more suited to lower-level hospitals. Patients with moderate severity made higher OOPE than patients with severe or extremely severe diseases. When adjusted for other variables, sponsored indigent members were more likely to make OOPE than other types of members. | Medium |
| **Thailand** | | | | | | | |
| (27), 2018 | To investigate the causal impact of Thailand’s Universal Healthcare Coverage Scheme on households’ consumption and saving behavior. | Thailand, individuals who were part of the VHI or uninsured prior to the implementation of UCS | Prepayment scheme – UCS | Impact evaluation  Descriptive statistics, regression analysis using difference-in-difference analysis | Pseudo-panel dataset using repeated cross-sectional data from Thailand’s Socioeconomic Survey and Health and Welfare Survey  2001, 2004, and 2007 | Results suggest that UCS has both an income effect and a risk reduction effect on savings. These effects are of opposite sign, thus canceling each other out. Regarding consumption, the two effects should lead to increases, which is confirmed in the analysis (in the long run). The UCS resulted in no significant changes in household’s consumption or saving behavior in the short run. In the long run, there is evidence of increased consumption, especially of durables, though there is still no significant change in total saving. | High |
| (28), 2015 | To estimate the impact on OOPE of a major reform in Thailand that greatly extended health insurance coverage to achieve universality while implementing supply-side measures intended to deliver cost-effective care from an increased, but modest, public health budget. | Thailand, IWs and their dependents | Prepayment scheme – UCS | Impact evaluation  Generalized Linear Model, difference-in-difference analysis | Secondary data analysis from the socioeconomic Survey  2000 and 2004 | The major health reform through UCS has greatly reduced exposure to medical expenditure risk (three fifths on average); the welfare gain from improved financial risk protection appears to be sufficient by itself to outweigh the efficiency cost of financing the reform (equivalent to 80-200% of the approximate deadweight loss from financing the reform). The reform reduced OOPE by 28% on average. The reform increased the proportion of Thais who received formal ambulatory care when sick. Increases in inpatient treatment were not achieved by bypassing primary care but was confined to urban locations. | Medium |
| (29), 2012 | To assess the magnitude and trend of government health budget benefiting the poor as compared to the rich UCS members. | Thailand, poor and rich UCS members | Prepayment scheme – UCS | Impact evaluation  Benefit incidence analysis | Secondary data analysis using the Health and Welfare Survey  2003 and 2009 | The minimum level of OOPE is in favor of the poor, which is reflected by the probability and level of payment for both outpatient and inpatient services, which is low among the poorer than the richer quintiles. Poorer quintiles had higher rate of using their entitlements. Government made substantial financial commitments; general government expenditure increased, while OOPE of total health expenditure reduced. The pro-poor government health spending is homogeneously distributed across four geographical regions, resulting from the homogeneity of district health systems development nationwide. | High |
| (30), 2020 | To examine the impact of Thailand’s UCS on health care use. | Thailand, UCS members | Prepayment scheme – UCS | Descriptive statistics, pooled logistic regression analysis, propensity score matching | Secondary data from the Health and Welfare Survey  2003 to 2005 | The uninsured group at all income levels had significantly lower levels of utilization of both inpatient and outpatient services in public facilities than after the UCS. Private facility usage did not change. The change in utilization of public facilities was seen for all genders and in all regions of the country, with larger increases in rural than in urban areas. Similarly, increases were seen for all age groups for outpatient care. Informal care and private care utilization decreased for all genders and regions. | High |
| (31), 2019 | To examine the impact of health shocks of different severity on IWs in Thailand who are entitled to comprehensive public medical care but lack social protection of earnings. | Thailand, IWs enrolled in the UCS | Prepayment scheme – UCS | Panel study  Stratified two-stage random sampling  Least squares regression and propensity score matching | Secondary data from the Thai Socio-Economic Panel Survey  2005 to 2007 | The onset of a health issue is estimated to reduce IWs’ probability of remaining in employment by about 4%. Hours worked per week are reduced by 13%. OOPE increased on average by 71% following the onset of a health problem, though starting at a modest initial level of OOPE (about $5 per month per household). THE of a household was raised by 38% compared to the baseline. Illness raised the percentage incurring CHE by 2.1%. Effects on medical spending increased with the severity of the illness. Occurring illness raised the probability of borrowing by 5.6% and the amount borrowed by a third. The probability of having a non-labor source of income also increased by 4.2% (e.g. family, friends, remittances) and the amount received increased by 31%. This suggests that informal insurance, i.e. financial support from friends and relatives partially fills the gap left by the absence of formal insurance of earnings. The estimated effects are larger for serious health issues. Overall, the combination of UHC and informal insurance does a reasonably good job of protecting living standards from the economic impact of illness. However, substantial economic risks associated with illness remain even after UHC is put in place in Thailand, including both the risk of substantial earnings losses and a residual medical expenditure risk. | High |
| (32), 2018 | To assess and compare the purchasing functions of the UCS and the CSMBS, and to draw lessons for low- and middle-income countries in their quest for progressive realization of UHC, in particular for efficiency improvement. | Thailand | Prepayment scheme – UCS | Cross-sectional mixed methods study  Purposive sampling  Narrative synthesis, qualitative content analysis | Document review of mainly unpublished grey literature (e.g. laws, regulations, governing board meeting minutes, annual reports of CSMBS and UCS) and published papers on the scheme performance; KIIs and small group discussions | The passive purchasing of the CSMBS system has resulted in a rapid cost escalation and overspending on the annual budget. Costs per member of the CSMBS system are substantially higher (about 4-fold) than costs per UCS member. The National Health Security Office which manages UCS purchasing carries out a number of strategic purchasing actions, including the application of closed ended budgets, promoting PHC gatekeeping, exercising collective purchasing power, and engaging views of UCS members in decision-making processes. | High |
| (33), 2015 | To analyze the impact of harmonization of reimbursement rates for medical emergency services on clinical outcomes in private hospitals. | Thailand | Prepayment scheme – UCS | Impact evaluation  Chi-square test, Kruskal-Wallis test, multiple logistic regression | Secondary data from the Emergency Claim Online database  April 2012 – June 2013 | Beneficiaries under the UCS were the worst-off as indicated by the regression analysis for non-trauma and trauma, and in terms of not improved or dead outcomes at discharge compared to beneficiaries of the CSMBS. The outcome rates of not improved including dead were highest in UCS for both trauma and non-trauma patients. Thus, adjusting payment mechanisms alone is inadequate to ensure equitable distribution of health outcomes in provision of emergency medical care by private providers. The analysis further found that patients accessing hospital services directly showed better improvement or lower in-hospital mortality compared with access through formal pre-hospital means. | Medium |
| (34), 2015 | To synthesize strategic purchasing experiences in the National Health Security Office responsible for the UCS in contributing to achieving UHC goals. | Thailand, UCS | Prepayment scheme – UCS | Document review  Content analysis | Analysis of secondary data gathered through a literature review | UCS applied a purchaser-provider split, allowing the National Health Security Office to enforce accountability by public and private providers to the UCS beneficiaries through active purchasing. A high level of financial risk protection was achieved through a comprehensive benefit package (low incidence of CHE and impoverishment). The District Health System network was contracted to provide services for the entire district population, using age-adjusted capitation. Strategically purchasing services from the District Health System network achieved a pro-poor utilization due to geographical proximity with minimal travel time and costs. Reimbursement of inpatient services using diagnostic related groups helped contain costs effectively; unbundling certain specialized services from closed-end payments and reimbursing them according to a fee schedule prevented potential under provision. | Medium |
| (36), 2019 | To review how the political economy and the concept of path dependency influenced the evolution of the UHC reform in Thailand. | Thailand, UCS | Prepayment scheme – UCS | Document review  Content analysis applying the Political Economy of UHC Reform Framework and the concept of path dependency, narrative synthesis | Analysis of secondary data gathered through a literature review | The path towards UHC was not straightforward. The early expansion of a strong PHC system set the foundation for future scale-up of UHC. However, incremental expansion of coverage to different pools with different payment systems has contributed to persistent fragmentation in the HF system. Path dependence from the contextual environment coupled with political economy factors related to competing interests influenced both the design and the implementation of UCS in 2002. This highlights the importance of early decisions given the fact that they can lead to “path inefficiencies”, which may hinder achievement of efficiency, equity, and quality of care. | Low |
| (36), 2013 | Given the centrality of the four inter-related features (general tax finance, universality principle, financial risk protection, and securing adequate funding) to ensuring an equitable outcome and financial risk protection, this study seeks to explain how and why these features came about. | Thailand, UCS | Prepayment scheme – UCS | Policy review  Purposive sampling  Framework analysis, narrative synthesis | Analysis of secondary document gathered through a literature review and primary data collection using KIIs  Q3-4 2011 | Continuous financial and political commitment was key to the decision of a tax-financed UCS and to implementation. Commitment to the party manifesto and Thailand’s fiscal capacity pushed the ruling party to adopt a general tax-financed scheme since collecting premiums from IWs was neither feasible nor politically palatable. The monopsonistic purchasing power exerted from the National Health Security Office helped control prices, increase patient access, and improve systems efficiency. UCS has been gradually owned by its population (75%) and is thus less subjected to political changes. Especially in the context of a rapidly ageing society, long-term financial sustainability must be carefully monitored and researched. | High |
| (37), 2020 | To report how historical precedence and the political situation in Thailand paved the way for taxation as the sole source of financing for the universal coverage scheme. | Thailand, individuals eligible for the UCS | Prepayment scheme – UCS | Review  Narrative synthesis | Analysis of secondary data gathered through a literature review | In Thailand, domestic government health expenditure, institutionalization of a tax-financed system, and strong political commitment were essential in achieving UHC. This, in addition to the introduction of a comprehensive benefit package, has promoted greater equity in utilization, health financing, and health outcomes. Additionally, the shift from supply-side to demand-side budgeting and the use of evidence has helped secure adequate resources, limit discretionary budget allocation, promote transparency, and improve accountability to citizens. | Low |
| (38), 2018 | To analyze the historical evolution of health systems development that culminated in the implementation of UHC in 2002, and to analyze the achievements of UHC and factors contributing to these achievements. | Thailand, individuals eligible for the UCS | Prepayment scheme – UCS | Review  Narrative synthesis | Analysis of secondary data gathered through a literature review | Thailand’s district health system with extensive geographical coverage of functioning PHC has provided a solid platform for implementing UHC and for translating UHC policy into pro-poor utilization and benefit incidence. In the context of its large informal sector, Thailand’s strategy of a tax-financed scheme provided a feasible and progressive route to achieve UHC. The comprehensive benefit package with no co-payment at the point of service has reduced OOPE, CHE, and impoverishment. The stringent HTAs for review of the benefit package and inclusion of new interventions has supported cost containment and efficiency improvements. Additionally, strategic purchasing organizations and mechanisms with well-targeted provider payment methods have supported efficiency, cost containment, and equitable health outcomes. | Low |
| (39), 2013 | To explore the burden of household OOPE on urban inhabitants with different socio-economic status and health insurance schemes in Nakhon Sawan Municipality. | Thailand, households in Nakhon Sawan Municipality | Prepayment scheme – UCS | Cross-sectional study design  Two-stage random sampling  Descriptive statistics; Mantel-Haenszel odds ratio | Primary data collection using a household survey  December 2008 | UCS provided coverage to 91% of the poor, though only 76.8% of the non-poor. The vast majority of the poor were UCS members (131 vs. 13 covered by other schemes or without insurance). In total, 13% of the respondents who were UCS members were poor, while 87% were non-poor. The morbidity rate of the poor was higher than the non-poor. UCS members faced lower risks of CHE than CSMBS and SSS members. | Medium |
| (40), 2011 | To analyze health service use, distribution, and determinants before and after the introduction of the UCS. | Thailand, individuals eligible for the UCS | Prepayment scheme – UCS | Cross-sectional study design  Descriptive statistics; decomposition analysis; generalized linear model | Secondary data from the Thai Health and Welfare survey  2001 and 2005 | Health center use increased between 2001 and 2005, and with the UCS establishing them as PHC gatekeepers, a strong tendency to be used primarily by the poor was intensified. The use of tertiary facilities fell substantially with poor people being mainly responsible for this trend; provincial/general hospitals were increasingly used by those who were economically better-off. Private clinics were increasingly utilized among the better off, and private hospitals continued to be used by a small minority who were markedly better-off. Overall, use of health centers was unequal and biased towards the bottom income quintiles. The use of community hospitals was pro-poor. Use of provincial/general hospitals was pro-rich, especially for individuals in the 4^th^ quintile (likely above-average earning individuals who are not yet enabled to make use of private hospitals). | Medium |
| **Vietnam** | | | | | | | |
| (41), 2020 | To explore people’s experiences, thought processes behind, and social contexts of insurance enrollment. | Vietnam, IWs insured / uninsured under the SHI | Prepayment scheme – SHI | Ethnographic study  Non-probability sampling  Narrative synthesis | Primary data collection through key informant interviews  August to September 2016 | Individuals carried out ‘domestic triaging’ by foregoing insurance coverage in anticipation of care responsibilities in their family (e.g. elderly care, school fees). Individuals also anticipated economic and environmental risks, which influenced their decision to purchase health insurance. Additionally, individuals engaged in community building activities (e.g. community-based rotating credit associations, ritual banquets), which required cash; individuals viewed such relationships as their primary source of economic and social support, outweighing the importance of purchasing health insurance. Individuals also anticipated health vulnerabilities and felt the need to have cash to quickly establish a relationship with the overburdened medical staff and get the care needed, rather than relying on health insurance. Lastly, individuals anticipated inequalities in the health system, in which using cash instead of health insurance opened doors to obtaining better quality care. | High |
| (42), 2020 | To examine the association between health insurance on health-care utilization and the burden of OOPE among people with reported noncommunicable diseases and on their households in Vietnam. | Vietnam, IWs insured under VHI | Prepayment scheme – SHI | Cross-sectional study  Systematic random sampling using stratified, cluster, and simple random sampling  Descriptive statistics, multivariate logistic regression | Primary data collection through cross-sectional household surveys  2015 | Individuals with VHI had a considerably (though not significantly) higher utilization rate of outpatient and inpatient care than uninsured individuals (46.3% vs. 20.1% and 18% vs. 7%). People with a reported noncommunicable diseases with VHI were 2.5 times more likely to use outpatient care and 2.0 times more likely to use inpatient care than those without health insurance. | Medium |
| (43), 2020 | To explore the experiences of and factors influencing enrolment in FHI among informal sector workers in a rural district in Vietnam. | Vietnam, individuals enrolled with or eligible for FHI | Prepayment scheme – FHI under SHI | Mixed methods study using a cross-sectional survey, focus group discussions and in-depth interviews  Random selection of communes; purposive recruitment of participants  Thematic analysis | Primary data collection through key informant interviews and focus group discussions  January to June 2016 | 25.1% of IWs had FHI. Enrolled individuals with FHI tended to be older, experiencing health problems, or perceived their occupational health risks as high; the latter reportedly felt safer and more confident in their lives and jobs through possession of health insurance. Participants perceived it as more economic to purchase FHI only when a family member was ill in order not to waste money. Individuals generally had a good understanding regarding the benefits of health insurance not only for them but for society at large (i.e. cross-subsidization). Participants did not agree with the policy requiring all household members to be enrolled. Participants perceived the quality of medical services from FHI to be low. For ‘normal sickness’, participants preferred self-treatment and self-medication and indicated FHI to be for major/serious health issues only; for the latter, primary health service cards were viewed as useless. Private, upper-level facility services were preferred. Larger families reported increased difficulties in joining the scheme due to high fees; financial support provided by government was not perceived to be sufficient. Procedures for group enrolment and referral and claim procedures were reportedly cumbersome and waiting time at health facilities reported to be high. | High |
| (44), 2020 | To 1) examine the development of SHI in Vietnam during 1992–2016 and its contribution as a financial mechanism towards the goal of achieving UHC; 2) highlight some key lessons in the roadmap to UHC; and 3) provide policy recommendations based on underlying economic principles, socioeconomic conditions, and institutional realities. | Vietnam, individuals eligible for the SHI | Prepayment scheme – SHI | Desk Review  Systematic literature searches  Narrative review of documents | Desk review of the government of Vietnam’s documents pertaining to SHI, data reported by the World Bank, and other publicly available sources for the period 1992 to 2016 | Study reviews the development of the SHI across 5 stages, i.e. 1992-1998, 1998-2005, 2005-2009, 2009-2014, and 2014 onwards. Between 2009-2014, the Health Insurance Law was comprehensively; coverage was expanded to fully subsidized member categories, a roadmap for compulsory enrolment of the entire population was established, the government released a master plan to achieve UHC, revision of co-payments, revision of provider payment mechanisms, and release of a decree withdrawing all government subsidization for public hospitals incurred outside SHI.  Starting in 2014, the Health Insurance Law was amended to reclassify the eligibility categories, eliminate the voluntary scheme, schedule premium increases, change the mechanism of collection of revenue, revised reimbursement prices, and revise the benefit package. | Low |
| (45), 2012 | To evaluate and compare equity in access to health care in rural health insurance system in Vietnam and China. | Vietnam, members and non-members of VHI, and health and health insurance managers | Prepayment scheme - VHI | Mixed methods study using a cross-sectional survey, focus group discussions and in-depth interviews  Multistage sampling for the quantitative strand, purposive samples for the focus group discussions and in-depth interviews  Descriptive statistics for quantitative data; framework approach for qualitative data | Primary data collection using household surveys and structured questionnaires  May to July 2006 | Significantly increased health service usage among members in all income groups as compared to non-members.  Long waiting period between paying money and receiving health insurance cards during which individuals were unable to use health services; incorrect information further delayed the process. Members perceived to receive poorer quality of services than non-members (e.g. waiting times, unavailability of medicines); they therefore tended to only use health insurance for inpatient but not for outpatient services. Individuals with chronic diseases were significantly more likely to be members than non-members. | Low |
| (46), 2020 | To review the different paths taken by China and Vietnam using WHO’s three dimensions, and to identify important lessons and best practices that other countries can adapt as they move towards UHC. | Vietnam, members of VHI and key health system informants | Prepayment scheme – VHI | Mixed methods study using a literature review, secondary data analysis, and in-depth interviews  Purpose and snowball sampling  Evaluation techniques not stated | Review and analysis of secondary data and primary data collection through in-depth interviews  June 2017 to January 2018 | Vietnam expanded population coverage gradually, prioritizing the poor, the near-poor, and other vulnerable populations in a pro-poor progressive universalism approach. The country aimed to approach UHC equitably, providing a comprehensive and universal service package for all enrollees; this promoted accessibility and equity. CHE rates decreased in Vietnam between 2004 to 2014, and the gap between urban and rural populations decreased. | Low |
| (47), 2018 | To assess willingness to pay for family-based social health insurance and its determining factors among informal workers in Vietnam. | Vietnam, uninsured households, and key health system stakeholders | Prepayment scheme – FHI | Cross-sectional study using the contingent valuations method  Systematic sampling  Log-linear regression | Primary data collection using a cross-sectional survey including contingent valuations method  April to October 2016 | The average annual WTP amount per household was lower than the current FHI annual premium; the gap increased with increases in the number of uninsured household members. WTP was significantly influenced by (1) monthly household income (increasing WTP with increasing income); (2) number of uninsured members (decreasing WTP with increasing number of household members); (3) sickness of household member in past 4 weeks (households experiencing sickness reduced WTP). Households with children <6 had an increased WTP (not significant). | Low |
| (48), 2018 | To analyze the underlying causes for the low family-based health insurance enrolment. | Vietnam, uninsured households, and key health system stakeholders | Prepayment scheme – FHI | Mixed methods study using a cross-sectional household survey and in-depth interviews  Systematic sampling for the quantitative strand; purposeful sampling for the qualitative strand  Descriptive statistics for quantitative strand; thematic analysis for qualitative strand | Primary data collection using a household survey and in-depth interviews  April to October 2016 | Main reasons for low enrolment were unaffordable contributions (especially for large households), perceived good health status, perceived poor quality of health services, and ineffective enrolment procedures (delays in reception of card and thus in their ability to seek care). High-risk households preferred to purchase health insurance only for the vulnerable household members, not the entire family. Over a third of respondents were unaware of FHI details (e.g. benefits, co-payments, contributions). Respondents perceived the benefits package to be reasonable. | Low |
| (49), 2012 | To measure the impact of voluntary health insurance on health care utilization and out‐of‐pocket payments. | Vietnam, individuals enrolled in VHI | Prepayment scheme - VHI | Impact evaluation  Logit regression with propensity score matching and difference-in-difference matching | Vietnam Household Living Standards Surveys (2004 and 2006) | VHI had a positive and statistically significant impact on healthcare utilization; the average annual outpatient visits increased by around 45% and average annual inpatient visits increased by around 70%. The effects of VHI on OOPE were not statistically significant. The effect of VHI is similar for groups who had a different health insurance status in 2004, thus implying that the impact of current VHI does not depend on individuals’ health insurance status in the previous period. | High |
| (50), 2015 | To evaluate the impact on cost and utilization of a shift from fee-for-service to capitation payment of district hospitals by Vietnam’s social health insurance agency. | Vietnam, insured and uninsured individuals | Prepayment scheme – SHI | Quasi-experiment  Log linear fixed effect model by ordinary least squares | Panel of annual hospital inventory surveys (2005-2011); Vietnam Household Living Standards Surveys (2006, 2008, 2010) | Adopting capitation in hospitals is estimated to have resulted in a reduction in recurrent expenditure per case (about 5%) and drug expenditure (>8%); this suggests that capitation encouraged hospitals to be more efficient (produce same quantity of outputs at lower cost). For uninsured patients, all coefficients were positive, indicating increased utilization after introducing capitation; inpatient care increased most significantly with a nearly 21% increase in the intensity of inpatient services provided to the uninsured. This is consistent with hospitals increasing provision to patients paying FFS when capitation is introduced. | Medium |
| (51), 2017 | To understand community preferences for a HBP among the uninsured in Vietnam. | Vietnam,  uninsured individuals | Prepayment scheme – SHI | Mixed methods study using a cross-sectional survey, a ranking exercise, and focus group discussions  Two-stage purposive sampling  Descriptive statistics, McNemar test, and paired samples t-test for the quantitative strand; content analysis for the qualitative strand. | Primary data collection using a cross-sectional survey, ranking exercises, and questionnaires for focus group discussions  June to July 2011 | Communities clearly identified high-cost services on rare occasions (e.g. inpatient care) and less costly services used on a frequent basis (e.g. drugs, tests, outpatient care) as services of primary attention. Preventive care, defined as routine check-ups and vaccinations, was valued as much as costly health services. The Vietnamese health system is currently largely centered around higher-level care with first-level providers largely underutilized; provides an indication of the misalignment between public health spending and community preferences and that the Vietnamese health system is not responsive to population preferences. Group dynamics were found to strongly influence and shift individual preferences; individuals and groups generally agreed to the HBP configurations that enhance collective social welfare. Mental health services were attributed very low priority. | High |
| (52), 2012 | To study coping strategies used for paying health care costs, assessing the effects of such costs on economic and health stability. | Vietnam, insured and uninsured individuals | Prepayment scheme – SHI | Cross-sectional study  Sampling method not clearly identifiable  Dichotomization, ANOVA, Chi-squared tests; bivariate associations between coping strategies; multivariate logistic regression | Primary data collection using a cross-sectional survey  July 2008 | Near-poor and non-poor informal sector respondents used five main kinds of coping strategies. To cope with inpatient treatment costs, they used savings, loans, their income, money borrowed from relatives/friends, or reduced their food consumption; near-poor patients pay a greater proportion of inpatient treatments for loans than with income, and a considerable proportion used food reductions. A considerable proportion of the near-poor and the non-poor funded loan repayments with additional loans. In higher cost categories (e.g. inpatient treatment, high-cost outpatient treatment), the proportion of treatments financed by loans was considerably higher. | Medium |
| (53), 2012 | To examine the impact of Vietnamese health insurance schemes on inpatient and outpatient health care access, costs, and health outcomes. | Vietnam, insured and uninsured individuals, and insured individuals who did not use health insurance | Prepayment scheme – SHI | Cross-sectional study  Sampling describe but method not clear  Multivariable analyses (ordinary least squares, logistic regression) | Primary data collection using a cross-sectional survey  July 2008 | For inpatient treatment, the greatest proportion of the uninsured was from non-poor households (60%); these also constituted the greatest proportion of the insured who used insurance. The near-poor constituted a greater proportion of the uninsured (30.7%) than the poor (15%), highlighting the lack of insurance coverage for this group. At 54.3%, non-poor households constituted the largest share of uninsured patients, suggesting that these do not decide to purchase VHI. The majority of insured individuals who did not use insurance reported seeking care at facilities not covered by their insurance (3/4 of treatments taken at private clinics). Treatment costs decreased significantly for the members of the VHI in all income groups; gifts, transportation costs and lost income did not decrease. | High |
| (54), 2017 | To identify WTP for the SHI scheme among persons whose enrollment is voluntary and to examine factors that influence their choice. | Vietnam, uninsured individuals who are eligible for the VHI | Prepayment scheme – SHI | Cross-sectional study  Two stage random sampling  Linear multiple regression | Primary data collection using a cross-sectional survey  August to December 2014 | The main reasons for refusal to join the SHI scheme were lack of money to buy a health insurance card, and the perception that health insurance was not necessary since the respondent was healthy. Most of the respondents lacked detailed knowledge about the SHI and its benefits (87.9%). The number of respondents willing to pay for health insurance decreased with an increase in the co-payment level. WTP increased with increases in the knowledge score, while it decreased for individuals with a chronic disease. | Medium |
| (55), 2017 | To estimate the health insurance coverage of the near-poor in rural Vietnam and identify the individual and household factors associated with health insurance status. | Vietnam, near-poor individuals insured and uninsured under the SHI | Prepayment scheme – SHI | Cross-sectional study  Multi-level random sampling  Descriptive statistics; Chi-squared test; binary logistic regression | Primary data collection using a household questionnaire  Project phase 2012-2015 | Compared to the uninsured, the insured tended to live in urban areas, to be older, to work as farmers or in other jobs, to rate their health as poor (suggesting adverse selection), to have good knowledge of health insurance, to evaluate health insurance premiums as low or medium, and to be interested in health insurance. Insured individuals were also more likely to live in single-parent households, to have one or more elderly at home, to have two or more females >18 in the household, and to live in permanent houses. Differences in gender, education, marital status, household size, and number of children in the household were not significant. Factors associated with enrolment were poor health status, higher knowledge of health insurance, interest in health insurance, one or more elderly in the household, and perception of premium costs to be low/medium; individuals living in temporary / semi-permanent houses were less likely. | Medium |
| (56), 2013 | To model static and dynamic health insurance choices in Vietnam between 2004 and 2006, to discuss empirical results, determinants, and dynamics of health insurance enrolment, and to discuss policy implications. | Vietnam, insured and uninsured individuals under the various SHI categories | Prepayment scheme – SHI | Longitudinal study  Stratified cluster sampling  Descriptive statistics; Multinomial Logit model | Longitudinal data from the Vietnam Household Living Standards Surveys (2004, 2006) | Individuals with poor health were more likely to join any of the categories in the SHI; having any illness in the 12 months prior to the survey significantly increases the probability of being included in the schemes. Individuals with a disability were more likely to enroll in the VHI than those without. Females were more likely to join the VHI because they tend to have a higher risk. Higher age was associated with higher probability of enrolment in all types of SHI. This suggests the presence of significant adverse selection in the VHI. Individuals in better-off households and with better education have a higher probability to have VHI than any compulsory scheme. | Medium |
| (57), 2014 | To evaluate the impact of SHI across population groups in Vietnam. | Vietnam, Uninsured and insured individuals under various SHI categories | Prepayment scheme – SHI | Cross-sectional study design  Stratified cluster sampling  Descriptive statistics; propensity score matching | Data from the Vietnam Household Living Standards Surveys 2006 | Insurance was found to increase the utilization of public healthcare, particularly inpatient care at district hospitals, across all groups. Self-employees enjoyed lower on-site medication expenditures compared to their uninsured counterparts. Self-treatment visits decreased (not significant). There is evidence that insurance reduces the incidence of CHE across target groups at the 10% and 25% thresholds (not significant and no effect at 40% threshold). | Medium |
| (58), 2014 | To describe how different provider payment systems are designed and implemented in practice across a sample of provinces and districts in Vietnam. | Vietnam, policymakers, purchasers, and providers | Prepayment scheme – SHI | Cross-sectional qualitative interview study  Purposive sampling  Analysis using the Provider Payment Diagnostic and Assessment Guide developed by the Joint Learning Network for UHC | Primary data gathered through qualitative interviews; quantitative secondary data gathered from hospitals and provincial social security offices | Global and line-item budgets, FFS, and capitation are the PPMs used in Vietnam. Most hospitals receive payment from two different purchasers through three different methods, creating a high degree of fragmentation and conflicting incentives. Budget norms vary widely across provinces. Hospitals tend to carefully manage their costs to generate a surplus since they are permitted to retain a portion of any surplus. For FFS, there is little guidance and fees are often set in an ad hoc way. Capitation bears little resemblance to internationally known capitation; hospitals can be reimbursed up to 60% of any overruns from their capitation funds calculated on an FFS basis. Capitation and FFS as implemented bear great resemblance. There is also a wide variation in capitated rates across provinces and population groups which is not related to health need, thus raising equity concerns. Respondents viewed none of the PPMs as bringing strongly positive results to the health system. Policymakers and purchasers generally had a more positive views on PPMs’ effects on equity, efficiency, quality. | High |
| (59), 2011 | To compare OOPE on outpatient care at a health facility between insured and uninsured patients as well as across various providers. | Vietnam, individuals insured under VHI | Prepayment scheme – SHI | Panel study with repeated cross-sectional studies  Stratified cluster sampling  Descriptive statistics; random effects modeling | Data from the Vietnam Household Living Standards Surveys 2004 and 2006 | Average OOPE varies greatly across health facilities, with increasing OOPE with increasing facility level. VHI members paid greatly reduced OOPE as compared to uninsured individuals (e.g. average 84 vs. 146 Vietnamese đồng), with greater reductions for those using lower level public health facilities. The use of private health facilities remained common among individuals with VHI with private clinics/hospitals being the single largest providers, accounting for close to 1/3 of all outpatient contacts by VHI members. VHI members had higher utilization rates than compulsory health insurance members. | High |
| (60), 2020 | To evaluate the impact of the Revised Health Insurance Law 2014 on the utilization of outpatient and inpatient care services, healthcare services utilization at different levels of providers, types of providers and types of visits across different entitlement groups | Vietnam, Uninsured and insured individuals under various SHI categories | Prepayment scheme – SHI | Cross-sectional study design  Stratified cluster sampling  Descriptive statistics; Propensity score matching | Secondary data from two waves of the Vietnam Household Living Standard Survey (2014 and 2016) | There was a statistically significant positive impact of the revised Health Insurance Law (2014) on the frequency of using health services across all subpopulations for inpatient, outpatient, and commune health station services, including VHI members. The effect was strongest or outpatient services at the district level. The policy had a positive impact on using public health facilities while reducing the frequency of private health facility utilization. | High |
| (61), 2020 | To examine the impact of SHI programs on the utilization of health care services OOPE in general and across different health care providers in particular. | Vietnam, Uninsured and insured individuals under the VHI and the heavily subsidized SHI categories | Prepayment scheme – SHI | Cross-sectional study design  Stratified cluster sampling  Descriptive statistics; Difference-in-difference analysis; propensity score matching | Secondary data from two waves of the Vietnam Household Living Standard Survey (2014 and 2016) | The use of health services at the district hospital increased significantly among the VHI group for both outpatient and inpatient services; members visited provincial hospitals less frequently. The probability of having outpatient and inpatient OOPE increased for the VHI members while it remained stable for the uninsured. VHI members had higher OOPE than members of the heavily subsidized SHI category. Compared to the uninsured, those enrolled in VHI were generally older, less likely to be males, unskilled workers, more likely to live in households with a high proportion of people above 60 years, have access to clean water and toilet, live in households with higher expenditure, and possess more assets (e.g. motorcycles, telephones, radios, televisions, and computers, and more likely to be ill a greater number of times for a higher number of days in the past 12 months). | High |
| (62), 2021 | To examine the determinants of CHE, especially health insurance participation status. | Vietnam, uninsured individuals and SHI members | Prepayment scheme – SHI | Cross-sectional study design  Stratified cluster sampling  Descriptive statistics, logistic regression | Secondary data from the Vietnam Household Living Standard Survey 2016 | CHE is more prevalent among households having members with severe illness or injury and in households with heads aged over 60 years. There is great inequality in the frequency of CHE across income quintiles with poorer households facing increased CHE risks. Households in rural areas and with unemployed household heads also face greater risks. Those enrolled in the VHI had the second highest incidence of CHE, after those enrolling in the heavily subsidized health insurance program, though still significantly lower odds of suffering CHE than non-insured households. Households participating in HIS, belonging to higher income quintiles, living in a province with more hospitals, having employed household heads, more members, and living in urban areas have lower odds of suffering CHE. Age of household head, ethnicity, and education level also have a statistically significant effect. | Medium |
| (63), 2014 | To identify the size of different hospital financing sources for different hospital services and their impact on the uninsured. | Vietnam, near-poor uninsured households | Prepayment scheme – SHI | Panel study  Linear regression; Hausman Fixed Random test | Aggregate statistics from hospitals extracted from the annual hospital statistical reports (2005-2008) | Up to 51% of outpatient visits and 45% of inpatient bed day costs are directly supported by the government either through the state budget or through ownership and thus being responsible for depreciation. This indicates a higher proportion of hospital unit costs are covered by the government, compared to 30% of total health expenditure covered by public expenditure on health. In the estimation of the impact of sharing the unit costs of hospital services, one inpatient treatment episode of surgical treatment at either central or provincial hospital levels; and of a non-surgical treatment at central hospital level immediately made the near-poor households who are uninsured and had to pay OOPE for treatment at risk of CHE. Just one surgical inpatient treatment at central hospital level exceeded the 15% threshold of the household’s average annual income. A shift from direct support to hospitals to the prepaid regime with free health insurance would provide a larger proportion of households of the vulnerable group with increased access to health services. | Medium |
| (64), 2016 | To shed light on the effects of information and premium subsidies on enrolment in a SHI scheme. | Vietnam, IWs and their families | Prepayment scheme – Voluntary SHI | Randomized experiment  Random assignment of households to control group or treatment groups  Regression analyses | Primary data collection through a baseline survey (April-May 2012) and an endline survey (June-July 2013) | Small effects which are not statistically significant; for instance, the combined intervention providing information and a subsidy raised enrolment by less than 1% and not significantly. Over 90% of the eligible individuals were still uninsured after receiving both interventions. Effects are smaller than expected by government officials surveyed. Concerns over adverse selection may be justified as higher enrolment rates were observed among the less healthy. Subsidies and information may exacerbate this problem by encouraging enrolment by ‘bad’ but not ‘good’ risks. | High |
| **Multiple countries** | | | | | | | |
| (65), 2019 | To provide an overview of the health financing challenges and reforms in the Western Pacific Region over the past decade. It then outlines the lessons learned and HF policy implications in Asia and Pacific island countries, and the ongoing and future directions for countries at different stages of their health financing system development. | Countries in the Western Pacific Region (Cambodia, Lao PDR, Malaysia, Philippines, Vietnam) | HF reforms towards UHC | Review  Narrative synthesis | Secondary data review | Countries in the region with large informal sector populations have been facing challenges in the expansion of population coverage to IWs. These have generally started expanding coverage within the formal sector, subsequently directed government funds to poor and vulnerable populations, and only then provided subsidies to the informal sector. Implementation issues that emerged include compliance with mandatory participation, income assessment and contribution collection, maintenance of registration systems, and challenges associated with cross-subsidization from formal to informal sector. For countries with NHI, their contribution rates have been slowly increasing over time. Government subsidies have played a crucial role in the expansion of the health financing reforms. | Low |
| (66), 2011 | To review health financing reforms in seven countries in southeast Asia that have sought to reduce dependence on out-of-pocket payments, increase pooled health finance, and expand service use as steps towards universal coverage. | Countries in Southeast Asia (Cambodia, Indonesia, Lao PDR, Malaysia, Philippines, Vietnam) | Health financing schemes in SEA that are aimed at reducing OOPE, increasing pooled health finance and expand utilization of services | Review  Narrative synthesis of selected secondary sources | Review of published literature and other government unpublished documents | Coverage of the non-poor and not-so-poor informal sector workers has remained a challenge in the region, despite that they make up large portions of the population in most countries. Several countries (e.g. the Philippines, Vietnam) seek to expand coverage through contributory schemes, i.e. “squeezed top-down”, while others use tax funding (e.g. Thailand), i.e. “squeezed bottom-up”. The choice between contributory and tax-financing schemes depends on political and health system contexts. In Malaysia, social solidarity mechanisms seem insufficient to overcome opposition, leaving the voices of informal sector workers unheard; collection of premiums from the informal sector was also reported to be difficult. | Low |
| (67), 2016 | To specifically assess health insurance subsidization via government budget transfers as a potential approach to expand UHC with a regional focus on Asia. | Countries in Southeast Asia and the Western Pacific region (Cambodia, Indonesia, Philippines, Thailand, Vietnam), poor and vulnerable groups, including IWs | Health insurance systems with government subsidization of contribution payments through the transfer of general government revenues | Review  Narrative synthesis along the WHO health financing framework | Review of published literature and (un-)published grey literature | The review identified eight countries in the regions utilizing a total of 14 different subsidization schemes. Countries most frequently covered children, the poor, and older persons, with mostly mandatory memberships at full subsidization rates. Several countries, among which China, India, Indonesia, the Philippines, Thailand, and Vietnam also offered subsidization schemes to IWs. Utilization rates for insured people were generally higher than for uninsured individuals, but still lower compared to insured formal sector employees. Population coverage rates are related with using broader eligibility criteria for the schemes; universalist approaches were shown to yield the highest total and group-specific enrolment rates. The authors conclude that targeted schemes may represent a starting point from which gradual extension of eligibility may eventually result in a subsidized scheme based on a universalist approach; such a path requires constant political commitment and resource mobilization. | High |

Abbreviations: BPJS-K = Badan Penyelenggara Jaminan Sosial Kesehatan; CBHI = community-based health insurance; CSMBS = Civil Servant Medical Benefit Scheme; CHE = catastrophic health expenditure; FFS = fee-for-service; FHI = family health insurance; HBP = health benefits package; HF = health financing; HIS = health insurance scheme; IW = informal worker; JKN = Jaminan Kesehatan Nasional; KII = key informant interview; NHI = National Health Insurance; NHIS = National Health Insurance Scheme; NHS = National Health Service; OOPE = out-of-pocket expenditure; PHC = primary healthcare; PPM = provider payment mechanism; Rp = Indonesian rupiah; SEA = Southeast Asia; SHI = Social Health Insurance; SHP = social health protection; SSS = Social Security Scheme; UCS = Universal Coverage Scheme; UHC = universal health coverage; VHI = Voluntary Health Insurance; WTP = willingness-to-pay.

**Table 8. Health benefit package design, co-payment requirements, and purchasing arrangements.**

| **Country, HF scheme** | **Health service coverage** | **Co-payments and comparison to other schemes** | **Strategic purchasing including provider payment mechanism(s)** |
| --- | --- | --- | --- |
| **Cambodia**  HEF extension | The benefits package is defined in the MOH Guidelines for the Benefit package and Provider Payment of the HEF for the Poor, 2018. It includes the full range of services defined in the MOH Clinical Practice Guidelines for each level of care.  Several high-cost interventions are excluded, including select treatments for cancer, organ transplants, cosmetic surgery, infertility treatments, and medications not included in the MOH essential medicines list. In practice, delivery of the benefit package is limited to what is available; most notably service provision for secondary prevention of noncommunicable diseases, and geriatric as well as palliative health services are missing.  The health benefit package further includes cash allowances for reimbursements of transportation costs, food allowance during admission, and funeral benefits [1–3]. | No co-payments are required at the point of service delivery; HEF extension beneficiaries can access all services provided by health facilities for free [1,2].  No co-payments at the point of use for civil servants and formal sector workers who are part of the contributory system, and similarly no co-payments for HEF beneficiaries [3]. | Strategic purchasing of health services is very limited. Rates for HEF (extension) members are paid based on a retrospective payment system using a fixed case-based payment and associated costs, which is reimbursed to facilities on a quarterly basis by the MOH. HEF income is then pooled at facilities with official user-fee revenue from non-cardholding members, of which 60% is distributed to healthcare workers as incentives; this thus hampers reimbursing actual costs, since these need to be inflated by 60%. The information collected in the PMRS, the system utilized for information management of the HEF (extension), does not allow for the quality of care to be assessed. Standard treatment guidelines are in place, but no mechanisms to verify their application are in place (e.g. patient dossiers), and hence it is not possible to purchase services pursuant to pre-defined quality of care standards [3,4]. |
| **Cambodia**  CBHI | The different CBHI schemes provide varying health benefit packages. In addition to medical benefits, some schemes offer additional support to patients for transportation, food, funeral, and other patient expenses. No waiting periods of preconditions are in place for insured beneficiaries [4]. | CBHI schemes generally do not require co-payments for members seeking health services delivered at contracted government health facilities; schemes reimburse facilities for health services delivered to enrolled patients [4].  No co-payments at the point of use for civil servants and formal sector workers who are part of the contributory system, and no co-payments for HEF (extension) members [1–3] | CBHIs act as third-party purchaser of government health services based on service-delivery contracts with performance standards. Employed provider-payment mechanisms are FFS and monthly capitation payments at hospitals (retrospective), and capitation and FFS at health centers (prospective). Levels of reimbursement are renegotiated between insurer (CBHI manager) and health providers on a regular basis. Risks are born largely by enrolled members / patients; providers generally face no risk in case of inadequate or delayed budget disbursement and service curtailment [4]. |
| **Indonesia**  JKN | The comprehensive basic health benefit package includes medical promotive, preventive, curative, and rehabilitative services at primary and secondary care levels, including essential medicines. Additionally, non-medical benefits (accommodation and ambulance services) are included with plans depending on membership category.  BPJS-K also has a negative list of services not covered, including services rendered at non-BJPS-K facilities, esthetic services, infertility services, orthodontic services, drug- and alcohol-related services, conditions that are self-inflicted or due to extreme hobbies, alternative and traditional treatment, contraceptives, and services during disasters and emergency situations [5–7]. | While the initial JKN regulation included a “no-cap, no-co-payment” rule for its benefit package provided patients followed the referral system put in pace, co-payments of Rp20,000 (class A and B hospitals) and Rp10,000 (class D hospitals) have been introduced in 2018 to pay the costs of minor illness services amid ongoing budget deficits of JKN. Additional payments are also required if JKN beneficiaries want a higher class of hospital accommodation.  Sponsored members are exempted from these co-payments [6–8]. | BPJS-K has the mandate to serve as strategic purchaser and has a very strong bargaining position since it is the only purchaser at national level, though it still faces challenges in living up to this role. For instance, the formulae for setting payment models and tariffs are stipulated with insufficient consultation with provider and professional associations, and strategies to control cost and quality are insufficient, particularly since all government providers must participate in JKN whatever their quality status.  PHC: A capitation payment system is used as provider payment to all PHC facilities registered with BJPS-K under which health facilities receive monthly prospective grants based on the number of registered enrollees in the health facility (includes consultation, simple laboratory tests, drugs for acute care); obstetric and neonatal services for family planning programs are reimbursed based on preset payments. No risk adjustment mechanisms are in place, though special base rates are paid to geographically depressed areas and special compensation mechanisms are available for districts/areas that are lacking supply-side resources. Commitment-based capitation based on three performance indicators payments are further disbursed quarterly. JKN has a tiered referral system, which requires members to seek services at the PHC facility they registered with to seek referral to higher-level facilities; a cap of 5% is placed on referrals to discourage misuse.  Secondary care: Hospitals are reimbursed using DRGs based on approved Indonesian case mix-based groups (INA-CBGs) reviewed annually. Private facilities are reimbursed on an FFS-basis based on written cooperation agreements. Total claims for hospitals have no upper limit as no global budget was applied to inpatient services, making cost containment difficult [6,9,10]. |
| **Lao PDR**  NHI | Health services at health center, district hospital, and provincial hospital level for acute, long term, and palliative care based on the Essential Services Package and with no limitation on cost.  Excludes preventive and promotive health services; cosmetic and transgender surgery; spectacles; and medicines not available at facility level due to supply shortages or because they are not covered by the scheme.  Does not duplicate benefits covered by other disease programs (e.g. malaria, tuberculosis, HIV/AIDS, employment injuries, traffic accidents) [11,12]. | Low co-payments for IWs in public health facilities:  Outpatient**:** Health centers: 5000 LAK ($0.56); District hospitals: 10,000 LAK ($1.13); Provincial hospitals: 15,000 LAK (1.7$)  Inpatient**:** Health centers: 5000 LAK ($0.56); Free for pregnant women; District and provincial hospitals: 30,000 LAK ($3.5)  Referrals: 30,000 LAK (USS$3.5) [12–14]  No co-payments at the point of use for formal sector workers part of the contributory system. Maternal and child health services are also exempted from co-payments [13,15]. | The strategic purchasing function has remained largely passive. NHI payment is in principle output-based but need, performance, and quality factors have yet to be incorporated into the formula. Additionally, there is a lack of contractual agreements between purchasers and service providers, and claim management is weak. Outpatient care is financed based on capitation payments, while case-based payments are applied for inpatient care (including food and transport); Maternal and Child Health services are similarly reimbursed on a per-case basis. For chronic diseases, risk-adjusted capitation payments are in place, though with cost-sharing requirements for high costs [15,16]. |
| **Malaysia**  NHS | As Malaysia’s public health system resembles an NHS system, it does not have an explicitly defined health benefits package and patients do not have any legally enforceable right to access specific services. The scope of services offered thus reflects historical decisions on in/exclusion of certain services, decisions taken at central level on the inclusion of new technologies, the local level of supply, and decisions made by providers.  The scope of health services offered is comprehensive, including health promotion and prevention, as well as curative and rehabilitative care. This includes maternal, neonatal, and reproductive health services, general outpatient care, STI and HIV/AIDS services, TB care, NCD services (diabetes, hypertension), dental services, lab services, and pharmaceuticals. Specific adolescent and elderly, environmental, school, prison, and occupational health, as well as rehabilitation programs. The introduction of new health technologies has been progressively informed by recommendations on the basis of HTA (though not determined) [17,18]. | Services are highly subsidized, and patients pay minimal nominal user fees according to a fee schedule set by MOH; three classes are available. For PHC visits, patients pay MYR1 (USD$0.24) and MYR5 ($1.21) for a specialist consultation; for inpatient admission, patients pay a maximum of MYR500 ($122) in the 3^rd^ treatment class. Drugs formally listed on the “blue book” are generally accessible (if available locally) without copayment [19].  The poor and other special groups (e.g. people living with disabilities, elderly) are exempt from paying user fees based on means-testing. | A variety of payment mechanisms are found in the health system, but little strategic purchasing. Purchaser and provider are not separated but both functions are assumed by MOH. The Treasury allocates annual operational and developmental line-item budgets to MOH, based on historical expenditure and new programs or policies approved under five-year Malaysia plans. Budgets cascade downwards from the MOH to responsible entities, including district health offices (manage public health and PHC facilities), hospitals, district dental offices, and pharmacies using global line-item-based budgets based on historical spending; allocations are based on health needs and historical spending patterns. Most input/expenditure categories in the line-item budgets are locked in and cannot be reallocated (e.g. staff salaries, hospital support services, pharmaceuticals, utility). Budget allocations are reviewed mid-year, which allows some flexibility to react to arising needs articulated by lower-level budget entities; unspent funds must be returned to the Treasury. This system does not link resources to results, hence disincentivizing efficiency improvements while incentivizing facilities to fully execute their budget yearly. The public health system thus depends largely on the work ethics of its workforce, combined with management tools (e.g. quality assurance programs).  Due to migration of trained staff to the private sector, especially medical specialists, the public sector is required to occasionally buy services in critically short-staffed areas (e.g. neurosurgery) at hefty prices, particularly in case of emergencies [20]. Moreover, MOH has purchaser-provider arrangements to a small number of outsourced non-medical support services (e.g. catering, laundry).  Malaysia has a national referral system in which PHC is the thrust of the healthcare system; secondary and tertiary care services have been growing in scope, and patients are mandated to seek referral to access these [17–19]. |
| **Myanmar**  SSS | Comprehensive benefit package at SSB facilities including medical treatment and delivery (outpatient care, inpatient care, medicines, laboratory, transportation in case of referral) for a maximum of 26 weeks. Members are further entitled to a sickness cash benefit of 60% of the average salary of the past four months paid for up to 26 weeks. Not designed on the basis of evidence about health needs and cost-effectiveness, nor on findings from Health Technology Assessments (HTAs) [21].  Does not duplicate benefits provided for free under the Social Security Law and Rules of 2012 (e.g. essential medicines, emergency care, institutional child delivery, free care for children <5) [22]. | No co-payments for workers. Retired workers pay a co-payment of 50% of the cost of treatment. No minimum qualifying period to obtain health benefits [22].  Not applicable as there are no other prepayment schemes in Myanmar [21–23]. | Purchasing arrangement model where SSB purchases services from facilities owned by them. Facilities receive a biannual budget allocation based on their own estimate of past consumption of medical supplies. The lack of purchaser-provider split compromises strategic purchasing and SSB’s leverage to properly negotiate provider-payment mechanisms and rates. The SSB’s capacity for provider selection based on performance and quality, and for enforcement of accountability of providers is further limited. Detailed analysis of the current reimbursement processes are lacking [21,24]. |
| **Philippines**  NHIP | Broadly defined benefits package including preventive, promotive, curative, rehabilitative, and palliative care for medical, dental, mental and emergency health services; this includes inpatient care, room and board, health services, diagnostic and laboratory services, prescription drugs and biologicals, and emergency transfer services.  Non-prescription drugs and medical devices, dependency and alcohol abuse treatment, cosmetic surgery, fourth and subsequent normal obstetrical deliveries, outpatient psychotherapy and counselling for mental disorders, cost-ineffective procedures as defined by PhilHealth, and optometric services are excluded.  The so-called Z-Benefits package is oriented to illnesses that are considered economically and medically catastrophic to patients and families. It covers major forms of cancer (breast, prostate, cervical, colon, leukemia), peritoneal dialysis, kidney transplantation, selected heart surgeries and orthopedic implants, benefits for children with disabilities, and care for premature and small newborns.  There has not been any systematic process to update the PhilHealth health benefits package with added benefits included on an ad hoc basis; political influences and lobbying have been reported as important drivers. The overall package is reportedly not geared towards addressing the disease burden. Processes resembling HTA are limited to medicines and the Z-Benefits package [25–29].  Under the UHC Act, HTA shall be established and institutionalized as a priority-setting mechanism to provide recommendations to PhilHealth and the Department of Health about the range of entitlements (e.g. medicines, devices, procedures, and services) that will be provided under the UHC scheme [26]. | A no-balance billing policy is in place, exempting indigent and sponsored members from co-payments when admitted in government health facilities. No such policy is in place for other members, including non-poor IWs, leaving room for co-payments to persist. Patients are required to advance costs and PhilHealth reimburses case-based rates up to pre-determined ceilings (based on disease severity and hospital level). Facilities can stipulate their own health service charges and NHIP members are required to pay the balance between total health service cost and NHIP benefits as OOPE (if any). This shifts the financial risk onto patients while not incentivizing technically efficient service delivery. This is exacerbated by the fact that PhilHealth case-based rates are based on average FFS claims rather than actual service costs [29]. | PhilHealth serves as a single purchaser though can technically not yet be considered as strategic purchaser of services; it accounts for a small portion of THE while OOPE is still the dominant source of health financing [29]. Further, the HF system involves complex layers of funding agents and governance mechanisms, leading to inefficiencies (e.g. lack of clarity in purchasing roles, deficiency in fund flows, purchasing overlap and double payment) [30].  PHC: PhilHealth purchases select PHC services from public sector facilities via a capitation arrangement. The management of PHC benefits includes a performance-based element and verification mechanisms before the municipality receives the financing for each household. However, many local government units did not comply and establish the necessary capitation trust fund and PHC capitation payments have therefore gone down considerably [25].  Secondary care: PhilHealth purchases services via case-based payments (DRGs) from public and private facilities. PhilHealth faces challenges in controlling price and volume since services are less standardized and include greater discretionary power from suppliers, which reduces allocative and technical efficiency. DRG rates are based on historical spending data and have not been updated since 2010 and therefore reflect health service costs inadequately. Utilization reviews are not yet conducted and fraudulent claims rarely detected and penalized [25].  To reduce inefficiencies, purchasing roles have been clearly specified in the UHC act, requiring adjustments of several HF elements. The Department of Health will be responsible for purchasing population-based services, while PhilHealth shall finance individual-based services (85% of THE), hence positioning PhilHealth as the national purchaser of health services. Providers will be contracted through prospective, performance-based payments with strong cost containment measures; case-based payments shall be upgraded to DRG-based global budgets [26]. |
| **Thailand**  UCS | Comprehensive benefits package including outpatient and inpatient care for health promotive, preventive, diagnostic, curative, rehabilitative (physical and mental), and palliative health services. Ambulance or transportation for patients and boarding expenses are also covered.  Some particular services from the negative list are excluded (e.g. cosmetic surgeries, medicines excluded from the essential medicines list, and services of unproven effectiveness such as stem-cell treatment). No publicly subsidized coverage for illness-related income losses is included [31,32].  The design of the benefit package is based on a transparent process involving a multitude of stakeholders from all sectors and based on an economic evaluation and assessment of the value, necessity, and feasibility of proposed services; the benefit package is reviewed regularly to achieve continuous improvements and expand benefits where possible [31,32]. | Services which are provided by contracted providers are delivered for free without any co-payment for UCS members. Additionally, no deductibles or maximum ceiling of benefit coverage are in place, and providers are not allowed any extra-billing, making services literally free under the scheme [32]. | NHSO serves as strategic purchaser for the UCS scheme to realize a purchaser-provider split, exerting monopsonistic purchasing power through price negotiations for medicines and medical products with assured quality; however, given that the majority of health services are provided by the public sector, NHSO’s role in health service delivery resembles more a finance facilitator enhancing coverage and efficiency, than a purchaser. Budgeting is close-ended for cost containment and reduction of supplier-induced demand, and to ensure fiscal sustainability. A mix of prospective, retrospective, and project-based provider payment methods are employed.  Outpatient care, prevention, and health promotion are financed through prospective capitation based on the number of people in the catchment areas (contracting unit); per capita payments are adjusted in line with the age composition. A referral system is in place, requiring patients to seek care at their registered service unit first to obtain a potential referral (excluding emergencies.  Inpatient care is compensated based on DRGs under a global budget; reporting data from health service providers are used as a basis for budget calculation. Several specialized, high-cost services for specific conditions are covered through a fee schedule (e.g. chronic kidney disease, HIV/AIDS). For long-term care and noncommunicable diseases, providers receive a fixed fee per patient. To receive reimbursement, providers submit data on provided health services as per DRG rates and the fee schedule [31,33,34].  Project-based payments are installment or block-grant payments for health programs aimed at addressing health challenges in specific areas; these are given to health facilities, local governments, or civil society organizations [31,33]. |
| **Vietnam**  SHI | SHI benefit package covers a broad range of basic preventative, curative and rehabilitative outpatient and inpatient services, and advanced diagnostic and therapeutic services (based on an inclusive list). The HBP is not designed on the basis of evidence about health needs and cost-effectiveness, nor on findings from HTAs, but rather based on historical provision [35,36]. Transport support is provided for vulnerable [36].  Coinciding with the decline of external financing for health, treatment for HIV and TB have been subsumed under the ambit of SHI reimbursement since 2019 [37].  Certain important health interventions (e.g. disease screening among asymptomatic individuals, substance abuse treatments) are neglected and neither covered by SHI nor state budget [38]. | Non-poor IWs are required to make co-payments of 20%. Near-poor IWs’ co-payments are at 5%; increased co-payments are payable for patients seeking care at facilities other than their registered facilities. A co-payment ceiling for people with 5-year continuous enrolment is in place. Reimbursement of expensive services is restricted to individuals with a minimum 6-months enrolment [36].  Individuals are required to register with and first attend their allocated PHC facility before referral to higher technical level facilities (e.g. provincial hospital) to receive the maximum reimbursement level. Higher co-payments apply for individuals who bypass lower-level referral facilities [36].  No co-payments for all fully subsidized members (the poor and veterans) [36]. | VSS has not yet effectively implemented its role as strategic purchaser. VSS purchases services from public and private sector health facilities that meet criteria of MOH and VSS, though contracts are based on a standard format, inhibiting VSS to impose additional conditions (e.g. improved quality, reduced fraud) [39].  Capitation rates were introduced for PHC providers at provincial and district levels; these are, however, set by SHI membership category rather than health risks or actual costs of care [40].  Reimbursements for curative care at secondary care level are largely paid on an FFS basis with few controls on the conditions and rules for payment; creates a perverse incentive for provider-induced demand, including overuse of (unnecessary) procedures, particularly expensive and high-tech procedures, and overprescribing medicines [35,37]. VSS placed a global cap on hospital payments as a cost containment measure, though this has been found to further erode financial protection; overspending in one year leads to a higher cap the next year, so hospitals have strong incentives to spend beyond the cap [40].  Global and line-item budgets are constructed based on the number of staff / beds and is supposed to cover staff salaries and operations of commune health stations. Norms vary widely across provinces [41]. |

Abbreviations: BPJS-K = Badan Penyelenggara Jaminan Sosial Kesehatan; DRG = diagnosis-related group; FFS = fee-for-service; HF = health financing; HTA = Health Technology Assessment; JKN = Jaminan Kesehatan Nasional; LAK = Lao Kip; MOH = Ministry of Health; MYR = Malaysian Ringgit; NHI = National health Insurance; NHIP = National Health Insurance Program; NHS = National Health Service; PHC = primary healthcare; PMRS = Patient Medical Record System; SSB = Social Security Scheme; THE = total health expenditure; UCS = Universal Coverage Scheme; VSS = Vietnam Social Security.

**Table 9. Utilization of health services under the HF schemes and related equity considerations.**

| **Country, HF scheme** | **Change in utilization of health services since introduction of the HF scheme** | **Equity considerations** |
| --- | --- | --- |
| **Cambodia**  HEF extension | No data available.^#^ | No data available. ^#^ |
| **Cambodia**  CBHI | CBHI increased the use of (covered) public health facilities by members (not significant) while simultaneously decreasing the use of uncovered and unregulated care. At the household level, insured households were 1% less likely to forgo care [42]*. | No data available. ^#^ |
| **Indonesia**  JKN | Utilization increased considerably since the introduction of JKN for both subsidized near-poor and self-enrolled IWs at both public and private facilities [10,43,44]*. | Despite the improvements, equity of access to healthcare remains low. Distance to hospitals is greatly influencing utilization with people in the poorest and remote areas being considerably less likely to use hospital care due to lower geographical access and disparities in health worker availability [6]*. Additionally, both the utilization rate and the average claim ratio of the IW group is considerably higher than those of all other groups, despite that their average premiums are lowest; this indicates risks of adverse selection and cross-subsidization from poor and near-poor to IWs (e.g. utilization rate poor/near-poor 4.11% vs. 32.49% IWs and claim ratios of 85% vs. 312% in 2019); about 23% of self-enrolled members register while they are ill [6,9,10,43,45]*. |
| **Lao PDR**  NHI | Increased overall utilization of public health services by 11-33% within 2 years since the introduction of the subsidized NHI for IWs, particularly with regards to outpatient services [13,16]*.  Married respondents, large households, and lower levels of household income significantly increased the probability of accessibility to health service utilization under the NHI. NHI is effectively easier to access for the general population compared to its predecessor (CBHI) [14]. | Significantly higher utilization rates for civil servants and formal sector workers than for IWs suggest that root causes of inequities in utilization have not been addressed and that public subsidies benefit the better off. Disadvantaged groups seem to lack awareness of their rights, including IWs [13,16,46,47]*. |
| **Malaysia**  NHS | No data available on health service utilization by IWs.^#^ | No data available for IWs.^#^ |
| **Myanmar**  SSS | No data available on health service utilization by IWs.^#^ | Public sector health services are pro-poor. Poorer quintiles, including IWs, are considerably more likely to utilize public health facilities for outpatient services; the income gradient in utilization is even more pronounced for inpatient services [18,19]*. |
| **Philippines**  NHIP | While utilization has increased with coverage expansion under the NHIP, overall PhilHealth benefit utilization has remained low, particularly among lower socioeconomic quintiles that comprise of large shares of IWs [29]*. | Non-poor IWs have increased utilization rates compared to other member categories. While this may indicate adverse selection, this also embodies risk-sharing and solidarity and is favorable from a UHC perspective [25,48]*. In contrast, the benefit payment shares of near-poor IWs whose contributions are sponsored by government is substantially lower than their membership share, indicating that benefits accrue disproportionately to richer members [48]*. |
| **Thailand**  UCS | (Per-capita) Utilization of curative health services increased for both outpatient and inpatient care after the introduction of UCS for previously uninsured women and men, including increases in preventive activities (check-ups). Additionally, a shift from private to public sources of care was observed for the previously uninsured [34,49]. | Equity of access increased with the UCS reform with larger increases in total outpatient and inpatient curative visits as compared to the increases in the number of new members. Additionally, a benefit incidence analysis confirmed the pro-poor government health subsidy under the UCS scheme [32]*. |
| **Vietnam**  SHI | Increases in population coverage under the SHI have led to an uptick in both utilization of inpatient and outpatient health services and SHI claims in all of the various membership categories [37,50–53]*. Regarding IWs in particular, health service usage among members of the VHI category of the SHI (now mandatory) significantly increased in all income groups as compared to non-members [52–54]. | Utilization rates among the near-poor continue to lag behind and result in large inequalities in utilization, hampered by both financial and nonfinancial barriers such as poor knowledge and lack of confidence in district hospital services [50,55]*. Moreover, members perceived to receive poorer quality of services than non-members (e.g. waiting times, unavailability of medicines); they therefore tended to only use health insurance for inpatient but not for outpatient services [54]. Additionally, direct subsidies to facilities are pro-rich since the richest quintile accounts for almost half of societal consumption [56]*. Access to essential health services among disadvantaged groups (e.g. those living in remote and mountainous provinces or ethnic minorities) is also far less and these groups have substantially worse health outcomes than the national average [37]*. |

Abbreviations: CBHI = community-based health insurance; IW = informal worker; JKN = Jaminan Kesehatan Nasional; NHI = national health insurance; NHIP = National Health Insurance Program; NHI = National Health Insurance; NHS = National Health Service; SHI = Social Health Insurance; UCS = Universal Coverage Scheme; VHI = Voluntary Health Insurance.

***** Data was partly taken from grey literature publications, the quality of which was not appraised.

**^#^** No such data was found in the literature searches. However, such data might exist in unpublished form or published in languages other than English.

**References**

1. Ministry of Health Cambodia. Guidelines for the Benefit package and Provider Payment of the Health Equity Fund for the Poor. Phnom Penh; 2018.

2. Ministry of Health Cambodia. Health Equity Fund Operation Manual. Phnom Penh; 2017.

3. Deutsche Gesellschaft für International Zusammenarbeit (GIZ) GmbH and World Health Organization (WHO). Toward strategic purchasing. Management of multiple schemes and purchasing decisions by the Cambodian National Social Security Fund. Bonn; 2020.

4. Annear P, Grundy J, Ir P, Jacobs B, Men C, Nachtnebel M, et al. The Kingdom of Cambodia Health System Review. Health System in Transition. 2015.

5. President of the Republic of Indonesia. Regulation of President of the Republic of Indonesia. No 12/2013. 111 Indonesia; 2013.

6. Mahendradhata Y, Trisnantoro L, Listyadewi S, Soewondo P, Marthias T, Harimurti P, et al. The Republic of Indonesia Health System Review. Health Systems in Transition. Vol 7 No. 1. Geneva; 2017.

7. President of the Republic of Indonesia. Regulation of the Minister of Health of the Republic of Indonesia. No 51/2018. Indonesia; 2019.

8. Deloitte Deloitte Asia Pacific Limited. Ensuring the Sustainability of JKN-KIS for the Indonesian People. Intended to cover the health costs of all Indonesians, the JKN-KIS national insurance program is now on the brink. How can this program survive? Jakarta; 2019.

9. Trisnantoro L, Hendrartini J, Susilowati T, Miranti PAD, Aristianti V. A critical analysis of selected healthcare purchasing mechanisms in Indonesia. Strateg Purch China, Indones Philipp. World Health Organization; 2016 Jun.

10. Agustina R, Dartanto T, Sitompul R, Susiloretni KA, Suparmi, Achadi EL, et al. Universal health coverage in Indonesia: concept, progress, and challenges. Lancet (London, England). 2019;393: 75–102. doi:10.1016/S0140-6736(18)31647-7

11. International Labour Organization. Moving towards universal social health protection. Lao People’s Democratic Republic (PDR). Geneva; 2019.

12. Chaleunvong K, Phoummalaysith B, Phonvixay B, Vonglokham M, Sychareun V, Durham J, et al. Factors associated with patient payments exceeding National Health Insurance fees and out-of-pocket payments in Lao PDR. Glob Health Action. 2020;13: 1791411. doi:10.1080/16549716.2020.1791411

13. United Nations ESCAP. Roles of Social Health Protection in achieving UHC in Lao PDR. Inclusive Social Protection Systems in Asia and the Pacific: An expert group meeting. Bangkok; 2019.

14. Bodhisane S, Pongpanich S. The impact of National Health Insurance upon accessibility of health services and financial protection from catastrophic health expenditure: a case study of Savannakhet province, the Lao People’s Democratic Republic. Heal Res policy Syst. 2019;17: 99. doi:10.1186/s12961-019-0493-3

15. Sorensen B, Masaki E, Panyanouvong T, Vongsonephet T, Thitsy S, Chamleunsab M, et al. Managing transitions: Reaching the Vulnerable while Pursuing Universal Health Coverage (Vol 2). Health financing assessment in Lao PDR (English). Washington D.C.; 2017.

16. Phoummalaysith B, Senchanthixay M, Phonvisay B, Sengdara L, Manivong D, Yu S, et al. National Health Insurance in Lao PDR: Accelerating Progress towards UHC. Vientiane; 2020.

17. Jaafar S, Noh K, Muttalib K, Othman N, Healy J, Maskon K, et al. Malaysia Health System Review. Health System in Transition. 2012.

18. Harvard T.H. Chan School of Public Health. Malaysia Health Systems Research Volume I. Contextual Analysis of the Malaysian Health System. Boston; 2016.

19. Yap W, Razif I, Nagpal S. Universal Health Coverage Study Series No. 42. Malaysia: A new public clinic built every four days. Washington D.C.; 2019.

20. Quek DK. The Malaysian Health Care System: A Review. Kuala Lumpur; 2014.

21. Tessier L, Guillebert J. Extending the network of health care facilities of the Social Security Board. Technical report on the feasibility of a Purchaser Provider Split. Geneva; 2015.

22. Tessier L, Thidar M. Evaluation of the operations of the Social Security Board, Ministry of Labour, Employment and Social Security of Myanmar. ILO-MDRI technical report. Geneva; 2014.

23. De L, Anh Q, Tsuruga I, Ruck M. An assessment of the social protection needs and gaps for workers in informal employment in Myanmar. Geneva; 2019.

24. Sein T, Myint P, Tin N, Win H, Aye S, Sein T. The Republic of the Union of Myanmar Health System Review. Health System in Transition. 2014.

25. Obermann K, Jowett M, Kwon S. The role of national health insurance for achieving UHC in the Philippines: a mixed methods analysis. Glob Health Action. 2018;11: 1483638. doi:10.1080/16549716.2018.1483638

26. Republic of the Philippines. Congress of the Philippines. Republic Act No. 11223. Universal Health Care Act. Philippines; 2019.

27. Republic of the Philippines. Congress of the Philippines. Republic Act No. 10606. Philippines; 2013.

28. Bredenkamp C, Buisman L. Universal Health Coverage in the Philippines: Progress on Financial Protection Goals. Policy Research Working Paper 7258. Washington D.C.; 2015. Report No.: Policy Research Working Paper 7258.

29. Dayrit M, Lagrada L, Picazo O, Pons M, Villaverde M. The Philippines Health System Review. Health System in Transition. Vol. 8 No. 2. New Delhi; 2018.

30. Nuevo C, Sigua J, Boxshall M. The Philippine UHC Law Series: Brief 3. Health Financing in the Philippines. Washington DC; 2020.

31. National Health Security Office. Thailand UHC & overview of the universal coverage scheme of the National Health Security Office. Bangkok; 2020.

32. Jongudomsuk P, Srithamrongsawat S, Patcharanarumol W, Limwattananon S, Pannarunothai S, Vapatanavong P, et al. The Kingdom of Thailand health system review. 2015.

33. National Health Security Office. The management of provider payments in the universal coverage scheme (UCS) in Thailand. Bangkok; 2020.

34. Tangcharoensathien V, Witthayapipopsakul W, Panichkriangkrai W, Patcharanarumol W, Mills A. Health systems development in Thailand: a solid platform for successful implementation of universal health coverage. Lancet (London, England). 2018;391: 1205–1223. doi:10.1016/S0140-6736(18)30198-3

35. Oan T, Phuong H. Strategic purchasing for universal health coverage: A critical assessment. Social Insurance Fund, Vietnam. Hanoi; 2016.

36. Le QN, Blizzard L, Si L, Giang LT, Neil AL. The evolution of social health insurance in Vietnam and its role towards achieving universal health coverage. Heal Policy OPEN. 2020;1: 100011. doi:https://doi.org/10.1016/j.hpopen.2020.100011

37. Teo H, Bales S, Bredenkamp C, Cain J. The future of health financing in Vietnam: Ensuring sufficiency, efficiency, and sustainability. Washington D.C.; 2019.

38. World Bank. Moving toward UHC: Vietnam - national initiatives, key challenges, and the role of collaborative activities (English). Washington D.C.; 2017.

39. Ministry of Health Vietnam. Joint Annual Health Review. Strengthening grassroots health care towards universal health coverage. Hanoi; 2015.

40. Barroy H, Jarawan E, Bales S. Universal Health Coverage for Inclusive and Sustainable Development : Country Summary Report for Vietnam. Washington D.C.; 2014.

41. Phuong NK, Oanh TTM, Phuong HT, Tien T Van, Cashin C. Assessment of systems for paying health care providers in Vietnam: implications for equity, efficiency and expanding effective health coverage. Glob Public Health. 2015;10 Supppl: S80-94. doi:10.1080/17441692.2014.986154

42. Levine D, Polimeni R, Ramage I. Insuring health or insuring wealth? An experimental evaluation of health insurance in rural Cambodia. Impact Analysis Series, No. 8. Paris; 2012.

43. Dartanto T. Universal Health Coverage in Indonesia: Informality, Fiscal Risks and Fiscal Space for Financing UHC. Tokyo; 2017.

44. Ly C. Essays on Universal Health Coverage in Indonesia. University of Pennsylvania. Publicly Accessible Penn Dissertations. 2018.

45. Dartanto T, Halimatussadiah A, Rezki JF, Nurhasana R, Siregar CH, Bintara H, et al. Why Do Informal Sector Workers Not Pay the Premium Regularly? Evidence from the National Health Insurance System in Indonesia. Appl Health Econ Health Policy. 2020;18: 81–96. doi:10.1007/s40258-019-00518-y

46. International Labour Organization. Integrating Social Health Protection Systems Lessons learned. Geneva; 2019.

47. World Health Organization Regional Office for the Western Pacific. Overview of Lao Health System Development 2009–2017. Manila; 2018.

48. Kaiser K, Bredenkamp C, Iglesias R. Sin Tax Reform in the Philippines. Transforming Public Finance, Health, and Governance for More Inclusive Development. Washington D.C.; 2016.

49. Limwattananon S, Neelsen S, O’Donnell O, Prakongsai P, Tangcharoensathien V, van Doorslaer E, et al. Universal coverage with supply-side reform: The impact on medical expenditure risk and utilization in Thailand. J Public Econ. 2015;121.

50. International Labour Organization. Expanding Social Health Protection: Towards Equitable Coverage in Viet Nam. Geneva; 2019.

51. Palmer M, Mitra S, Mont D, Groce N. The impact of health insurance for children under age 6 in Vietnam: A regression discontinuity approach. Soc Sci Med. 2015;145: 217–226. doi:10.1016/j.socscimed.2014.08.012

52. Thuong NTT. Impact of health insurance on healthcare utilisation patterns in Vietnam: a survey-based analysis with propensity score matching method. BMJ Open. 2020;10: e040062. doi:10.1136/bmjopen-2020-040062

53. Thuong NTT, Huy TQ, Tai DA, Kien TN. Impact of Health Insurance on Health Care Utilisation and Out-of-Pocket Health Expenditure in Vietnam. Biomed Res Int. 2020;2020: 9065287. doi:10.1155/2020/9065287

54. Liu X, Tang S, Yu B, Phuong NK, Yan F, Thien DD, et al. Can rural health insurance improve equity in health care utilization? a comparison between China and Vietnam. Int J Equity Health. 2012;11: 10. doi:10.1186/1475-9276-11-10

55. Somanathan A, Tandon A, Dao H, Hurt K, Fuenzalida-Puelma H. Moving toward Universal Coverage of Social Health Insurance in Vietnam. Assessment and Options. Washington D.C.; 2014.

56. Barroy H, Jarawan E, Bales S. Vietnam: Learning from Smart Reforms on the Road to Universal Health Coverage. Discussion Paper. Geneva; 2014.
